# Rapid antigen-based and rapid molecular tests for the detection of SARS-CoV-2: a rapid review with network meta-analysis of diagnostic test accuracy studies

**DOI:** 10.1186/s12916-023-02810-0

**Published:** 2023-03-29

**Authors:** Areti Angeliki Veroniki, Andrea C. Tricco, Jennifer Watt, Sofia Tsokani, Paul A. Khan, Charlene Soobiah, Ahmed Negm, Amanda Doherty-Kirby, Paul Taylor, Carole Lunny, Jessie McGowan, Julian Little, Patrick Mallon, David Moher, Sabrina Wong, Jacqueline Dinnes, Yemisi Takwoingi, Lynora Saxinger, Adrienne Chan, Wanrudee Isaranuwatchai, Bryn Lander, Adrienne Meyers, Guillaume Poliquin, Sharon E. Straus

**Affiliations:** 1 Knowledge Translation Program, Li Ka Shing Knowledge Institute, St. Michael’s Hospital, Unity Health Toronto, 209 Victoria Street, East Building, Toronto, ON M5B 1T8 Canada; 2 Institute for Health Policy, Management, and Evaluation, University of Toronto, Toronto, ON Canada; 3 Epidemiology Division & Institute of Health Policy, Management, and Evaluation, Dalla Lana School of Public Health, University of Toronto, Toronto, ON Canada; 4 Queen’s Collaboration for Health Care Quality: A JBI Centre of Excellence, Kingston, Canada; 5 School of Education, University of Ioannina, Ioannina, Greece; 6 University of Alberta, Edmonton, AB Canada; 7 Patient Partner, Strategy for Patient Oriented-Research Evidence Alliance (SPOR EA), Toronto, Canada; 8 University of Ottawa/Université d’Ottawa, Ottawa, ON Canada; 9 University College Dublin, Dublin, Ireland; 10 Ottawa Hospital Research Institute/Institut de Recherche de L’Hôpital d’Ottawa, Ottawa, ON Canada; 11 University of British Columbia, Vancouver, BC Canada; 12 University of Birmingham, Birmingham, UK; 13 Sunnybrook Research Institute, Toronto, ON Canada; 14 Ministry of Public Health, Nonthaburi, Thailand; 15 Health Canada (Ottawa)/Santé Canada (Ottawa), Ottawa, ON Canada; 16 Public Health Agency of Canada/Agence de La Santé Publique du Canada, Ottawa, ON Canada; 17 Department of Geriatric Medicine, University of Toronto, Toronto, ON Canada

**Keywords:** Diagnostic test accuracy, Rapid tests, COVID-19, SARS-CoV-2, Network meta-analysis, Rapid review

## Abstract

**Background:**

The global spread of COVID-19 created an explosion in rapid tests with results in < 1 hour, but their relative performance characteristics are not fully understood yet. Our aim was to determine the most sensitive and specific rapid test for the diagnosis of SARS-CoV-2.

**Methods:**

Design: Rapid review and diagnostic test accuracy network meta-analysis (DTA-NMA).

Eligibility criteria: Randomized controlled trials (RCTs) and observational studies assessing rapid antigen and/or rapid molecular test(s) to detect SARS-CoV-2 in participants of any age, suspected or not with SARS-CoV-2 infection.

Information sources: Embase, MEDLINE, and Cochrane Central Register of Controlled Trials, up to September 12, 2021.

Outcome measures: Sensitivity and specificity of rapid antigen and molecular tests suitable for detecting SARS-CoV-2.

Data extraction and risk of bias assessment: Screening of literature search results was conducted by one reviewer; data abstraction was completed by one reviewer and independently verified by a second reviewer. Risk of bias was not assessed in the included studies.

Data synthesis: Random-effects meta-analysis and DTA-NMA.

**Results:**

We included 93 studies (reported in 88 articles) relating to 36 rapid antigen tests in 104,961 participants and 23 rapid molecular tests in 10,449 participants. Overall, rapid antigen tests had a sensitivity of 0.75 (95% confidence interval 0.70–0.79) and specificity of 0.99 (0.98–0.99). Rapid antigen test sensitivity was higher when nasal or combined samples (e.g., combinations of nose, throat, mouth, or saliva samples) were used, but lower when nasopharyngeal samples were used, and in those classified as asymptomatic at the time of testing. Rapid molecular tests may result in fewer false negatives than rapid antigen tests (sensitivity: 0.93, 0.88–0.96; specificity: 0.98, 0.97–0.99). The tests with the highest sensitivity and specificity estimates were the Xpert Xpress rapid molecular test by Cepheid (sensitivity: 0.99, 0.83–1.00; specificity: 0.97, 0.69–1.00) among the 23 commercial rapid molecular tests and the COVID-VIRO test by AAZ-LMB (sensitivity: 0.93, 0.48–0.99; specificity: 0.98, 0.44–1.00) among the 36 rapid antigen tests we examined.

**Conclusions:**

Rapid molecular tests were associated with both high sensitivity and specificity, while rapid antigen tests were mainly associated with high specificity, according to the minimum performance requirements by WHO and Health Canada. Our rapid review was limited to English, peer-reviewed published results of commercial tests, and study risk of bias was not assessed. A full systematic review is required.

**Review registration:**

PROSPERO CRD42021289712

**Supplementary Information:**

The online version contains supplementary material available at 10.1186/s12916-023-02810-0.

## Summary box

What is already known on this topic

- Incorrect identification of SARS-CoV-2 may lead to unnecessary further testing, isolation, undue stress, loss of productivity, and school absences, so it is essential to have accurate testing.
- A polymerase chain reaction (PCR) test, completed in a lab, is considered a reference standard of testing, but it is costly to do and, in some settings with capacity challenges, it can take a day or more to get an answer.
- There are two broad categories of rapid tests that can deliver results quickly, often in less than an hour—rapid molecular tests and rapid antigen tests; but it is unclear which rapid tests are most accurate and what circumstances might make rapid tests more or less accurate.

What this study adds

- Our findings showed that rapid molecular tests are more accurate than rapid antigen tests and that rapid antigen test sensitivity might increase with the use of nasal samples or mixed samples (with a combination of samples from the nose, throat, and saliva) and decrease with the use of nasopharyngeal (from the area behind the nose and about the back of the throat) sample.
- Our results must be interpreted with caution because they are based on a rapid review, which used a simplified evidence synthesis process and included a limited number of studies; a full systematic review is required to confirm these preliminary results.

## Background

Countries around the world use rapid tests to help reduce the spread of the severe acute respiratory syndrome coronavirus 2 (SARSCoV-2). Rapid tests are intended to quickly (< 1 hour) identify a current infectiousness with SARS-CoV-2, the coronavirus that causes the COVID-19 disease. Rapid tests can help in part breaking unidentified chains of transmission. There are several types of rapid tests (e.g., antigen rapid tests and molecular rapid tests). Antigen tests identify proteins on the virus; they may come in disposable plastic cassettes, similar to pregnancy tests, or may require a portable analyzer (e.g., Quidel Sofia 2). Rapid molecular tests detect the virus’ genetic material in a similar way to laboratory methods but use smaller devices that are easy to transport or to set up outside of a laboratory. Both tests use nose, saliva, tongue, buccal, or throat samples. Since antigen tests detect viral proteins, they need more virus to be present in the sample. On the contrary, molecular tests can detect tiny amounts of genetic material in the sample. Many point-of-care tests are self-tests simple for people to use and interpret, without complicated equipment or lab analysis. Rapid tests are less expensive and deliver a result in an hour rather than a day or more. But are their results accurate enough to guide management and infection prevention and control?

The reference standard test for SARS-CoV-2 infection is a result from a laboratory-based polymerase chain reaction (PCR) test (or reverse transcription [RT]-qPCR). However, logistical challenges in accessing timely diagnostic PCR testing in times of high community transmission because of capacity issues highlights the importance of having access to additional diagnostically accurate rapid testing modalities (e.g., see Priority Strategies [1] from Government of Canada). Many studies and reviews have evaluated the accuracy of rapid tests, and we know that rapid tests are not always as accurate as PCR tests [2–7]. What we do not know is which rapid tests are the most accurate, and what circumstances might make rapid tests more or less accurate. Diagnostic test accuracy network meta-analysis (DTA-NMA) allows us (a) to draw inferences about differences in accuracy between tests that previously have not been compared, (b) rank all the available tests according to their diagnostic accuracy using all available evidence and borrowing strength from the network of studies, and (c) increase precision by considering all available evidence in a single model. Even in the case of a small number of studies informing a test (e.g., fewer than four), in contrast to DTA meta-analysis (i.e., the bivariate model), where estimation problems may occur (e.g., obtaining unreliable parameter estimates or lack of convergence) [8], DTA-NMA produces estimates for the included tests.

Obtaining quick and accurate results within one clinical encounter allows faster decisions about isolation and healthcare interventions (including early therapies) for those with positive test results, and contact tracing can begin immediately. The aim of this rapid review and DTA-NMA was to determine sensitivity and specificity of rapid antigen and rapid molecular tests for the diagnosis of the SARS-CoV-2 infection (COVID-19 disease) in adults and children according to the reference standard PCR test.

## Methods

We conducted a rapid review and NMA of DTA studies to synthesize different sources of evidence from a network of studies that compare different COVID-19 rapid tests. We followed the Preferred Items for Systematic Reviews and Meta-analysis (PRISMA) extension for DTA [9] (see Additional files 1 and 2) and PRISMA-NMA [10] (see Additional file 3) for reporting our rapid review DTA-NMA findings.

### Protocol

We developed our protocol using the Cochrane Handbook for Systematic Reviews of Diagnostic Test Accuracy and revised it with feedback from decision-makers within Health Canada, Public Health Agency of Canada, Ministry of Public Health in Thailand, and Irish Department of Health, as well as content experts, research methodologists, and statisticians. We used the PRISMA-P [11] guidelines and followed the World Health Organization (WHO) guide to rapid reviews [12] and registered our protocol with PROSPERO (CRD42021289712).

### Electronic searches

We searched Embase, MEDLINE, and EBM Reviews—Cochrane Central Register of Controlled Trials up to September 2021 for RCTs and observational studies (both retrospective and prospective studies) assessing any rapid antigen and any rapid molecular test to detect SARS-CoV-2. The literature search was developed by an experienced librarian (Ms. Skidmore; see Appendix 1—see Additional file 4), peer-reviewed by another librarian (Ms. Campbell) using the Peer Review of Electronic Search Strategies Checklist prior to the search execution [13], and completed on September 12, 2021.

### Eligibility criteria

RCTs and cohort studies published in English, with available 2 × 2 data (i.e., true positives, true negatives, false negatives, false positives) for analysis were eligible. We included studies from December 2019 up to September 2021, as shown below:

- *Population*: Both adults and children screened for SARS-CoV-2 infection or suspected of having a current SARS-CoV-2 infection, vaccinated or not.
- *Target condition*: SARS-CoV-2 infection.
- *Index tests*: We included studies evaluating one or more COVID-19 rapid antigen test or rapid molecular test used for screening of asymptomatic individuals or the diagnosis of SARS-CoV-2 infection in symptomatic individuals. We considered point of care or lab-based tests with turnaround times producing a result in less than an hour, without special sampling procedures (e.g., bronchoscopy or deep sputum), and limited to commercially available tests in any country, as determined in the publication or in the 360Dx regularly updated tracker [14].
- *Reference Standard*: We considered assays using PCR as a reference standard.
- *Study design*: We included RCTs and observational studies (i.e., single group diagnostic accuracy studies; prospective and retrospective). We included studies that provided the 2 × 2 table or joint classification-tables of index tests. We excluded small studies with fewer than ten cases and ten non-cases to minimize unreliable or biased estimates of sensitivity and specificity [15].
- *Outcome*: Sensitivity and specificity of rapid antigen and molecular tests suitable for screening and diagnosing SARS-CoV-2.

We excluded case–control studies, studies not using PCR as the reference standard, and studies not providing the 2 × 2 table in their publication.

### Search and study selection

We used our proprietary online tool, Synthesi.SR [16], to perform all levels of screening. To ensure reliability, all reviewers conducted a training exercise of 50 citations at level one and 15 articles at level two before screening, independently. When high agreement (> 75%) was observed, one team member (AAV, CS, ST, DN) screened each title and abstract for inclusion. After pilot-testing the screening criteria, one team member (AAV, CS, PK, AN, ST) reviewed the full-text of potentially relevant articles to determine inclusion.

### Data extraction

One review author (AAV, PK, CS, AN) performed data extraction using a predefined data extraction form, which was independently verified by a second reviewer. We pilot tested the form before proceeding to data abstraction using five full-text articles. We extracted the 2 × 2 (or joint classification tables, where available) and data characteristics of the participants, tests, and the study, as shown in Table 1. Conflicts were resolved by team discussion.

**Table 1:** Characteristics for data abstraction

Group	Data abstracted
Participant characteristics	Symptom status (symptomatic, asymptomatic); Duration of symptoms (in days); Age of test recipient; General public vs. Healthcare workers; Proportion of females/women (i.e., sex and gender of person being tested); Ethnicity; COVID-19 Vaccination status; Vaccine type; Number of vaccinations for COVID-19; Time of last vaccination
Test characteristics	Test name; Manufacturer; Sample type (e.g., saliva, nasopharyngeal swab, buccal/tongue, nasal); Sample condition; Time required for a test; Sample storage; Reference Standard; Test operator (e.g., nurse, self-testing); Who interpreted the results (e.g., nurse, self-testing); 2 × 2 table; Sensitivity, specificity, positive predicted value, negative predictive value with corresponding uncertainty measures (e.g., 95% CI)
Study characteristics	Year of publication; Journal name; Country where study completed; Study design; Study setting (school, community, health facilities [e.g., hospital]); Setting of participant recruitment; Study duration; Recruitment dates

### Risk of bias and applicability appraisal

We did not perform a risk of bias appraisal of the included studies due to time constraints.

### Data analysis methods

For the diagnostic accuracy of each index test separately, we used the 2 × 2 table of the individual studies as defined by the results of the index test against the reference standard. For each index test, a random-effects DTA meta-analysis was performed using the bivariate model to estimate a summary sensitivity and specificity with associated 95% CIs [8]. For completeness, we also performed the bivariate model in a Bayesian framework, using the model presented in the Cochrane Handbook for Systematic Reviews of Diagnostic Test Accuracy with vague priors for all model parameters [17]. For fewer than four studies informing an index test or evident heterogeneity between studies, only a descriptive analysis was performed, and results were presented in forest plots [8].

When the eligible studies formed a network of index tests, we performed a random-effects DTA-NMA to use the totality of evidence in a single model. A reference standard defines the presence or absence of SARS-CoV-2, and a network comprises index tests only [18]. We performed a Bayesian DTA-NMA model requiring the 2 × 2 table of the results of each index test against the reference standard, accounting for correlation between tests from the same study (Appendix 2—see Additional file 4) [19]. We estimated sensitivity and specificity for each test along with their 95% credible intervals (CrIs) and between-test and between-study heterogeneity. The minimum performance requirement for a test was sensitivity of ≥ 0.80 and specificity of ≥ 0.97, in accordance with guidance from WHO and Health Canada [20, 21]. We also calculated the Diagnostic Odds Ratio (DOR) within the DTA-NMA, to rank their performance; the higher the DOR, the better the test.

To explore heterogeneity, we visually inspected forest plots (variability in sensitivity and specificity, and confidence intervals overlapping between studies). We investigated potential sources of heterogeneity that may influence diagnostic accuracy using subgroup analysis (i.e., splitting included studies into subsets according to a specific characteristic) or meta-regression (i.e., adjusting for a covariate in the meta-analysis model) according to the following potential effect modifiers: symptom status (asymptomatic vs symptomatic), sample type (e.g., saliva, nasal swab), participant type (e.g., general public, healthcare worker), and rapid molecular test category (i.e., rRT-PCR, PT-Isothermal, RT-Lamp). When there were more than ten studies of antigen or molecular tests, separately, we explored heterogeneity by adding age as a covariate term in a meta-regression analysis to assess differences in accuracy.

A key assumption in DTA-NMA is that data are missing at random (MAR), which is inter-related with the transitivity assumption, assuming that potential clinical and methodological effect modifiers (e.g., participant age, test sample type) are on average similar across the different comparisons [22]. Violation of transitivity can cause statistical inconsistency between direct and indirect evidence. Several methods have been proposed to assess consistency in NMA of interventions [23–25], but these methods have not yet been applied to DTA networks. We compared the distribution of effect modifiers across the different test comparisons visually. We performed DTA meta-analyses and meta-regression using the *mada* package [26] in R version 2.21.7. We also conducted DTA meta-analyses within a Bayesian framework through the *rjags* package in R [27, 28]. Finally, we performed DTA-NMAs using *rstan* package in R [29] and the code provided by Nyaga et al. [19].

## Results

### Literature search and study characteristics

**Fig. 1:**
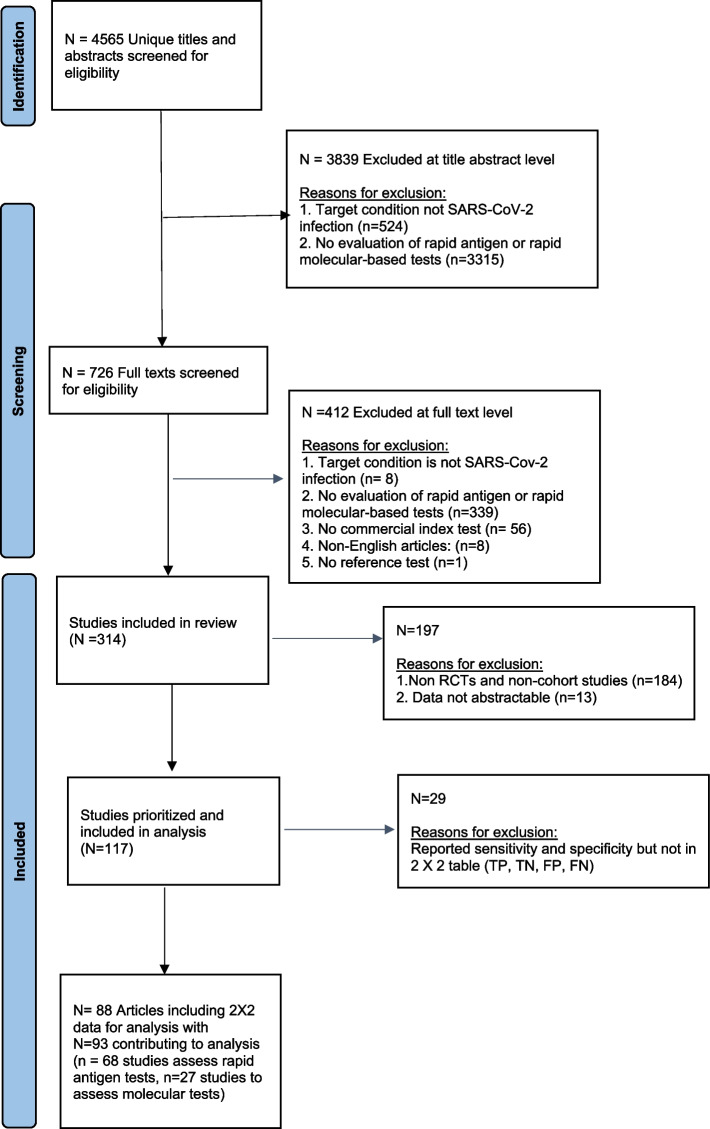
Flowchart of included studies in the rapid review

Our preliminary literature search yielded 4,565 titles and abstracts; 314 articles were eligible (Fig. 1). The identified 314 citations evaluated rapid antigen and rapid molecular tests to detect SARS-CoV-2. Of these, we included RCTs and cohort studies (prospective and retrospective) with available 2 × 2 data for analysis. Our analysis included 93 studies (reported in 88 articles; see Appendix 3—see Additional file 4): 68 studies assessed rapid antigen tests (composed of 55 single-test studies, eight paired-test studies, and five multi-test studies), and 27 studies assessed rapid molecular tests (comprised of 23 single-test studies, three paired-test studies, and one multi-test study). Two studies assessed both types of rapid tests.

We included evaluations of 36 different rapid antigen tests and 23 rapid molecular tests, with a total of 115,410 participants (104,961 participants in antigen tests; 10,449 participants in molecular tests; Appendix 4—see Additional file 4). The included studies were conducted in 28 countries (16 [17%] North America; 56 [60%] Europe; 18 [19%] Asia). The rapid antigen test most frequently (16 [16.3%]) assessed in the included studies was the PanBio COVID-19 Ag Rapid test (Abbott), and the most frequent rapid molecular test (5 [14.7%]) was the Xpert Xpress (Cepheid, Appendix 5—see Additional file 4).

### Statistical analysis

#### Meta-analysis

We conducted a DTA meta-analysis for the rapid tests that were informed by at least four studies, regardless of symptom status. Among the rapid molecular tests, we were able to meta-analyze data for the Xpert Xpress (Cepheid) test, including five studies and 763 participants (sensitivity 0.98 with 95% confidence interval [0.94, 1.00], specificity 0.98 [0.94, 0.99]). Similarly, we performed a DTA-meta-analysis for four rapid antigen tests (Table 2):

- PanBio COVID-19 Ag Rapid test (Abbott), including 16 studies and 32,151 participants (sensitivity 0.72 [0.61, 0.81], specificity 0.99 [0.99, 1.00])
- SARS-CoV-2 Rapid Antigen Test (Roche), including seven studies and 6065 participants (sensitivity 0.77 [0.55, 0.90], specificity 0.99 [0.96, 1.00])
- Standard F COVID-19 Ag test (SD Biosensor), including five studies and 6428 participants (sensitivity 0.65 [0.50, 0.78], specificity 0.98 [0.97, 0.99])
- Standard Q COVID-19 Ag test (SD Biosensor), including 13 studies and 8740 participants (sensitivity 0.72 [0.53, 0.86], specificity 0.99 [0.98, 1.00])

**Table 2:** Summarized results from the bivariate DTA meta-analysis model

			**Summary estimates**		**Heterogeneity standard deviation**	
**Type**	**Test**	**# Studies (# patients)**	**Sensitivity (95% CI)**	**Specificity (95% CI)**	**Sensitivity**	**Specificity**
Rapid molecular test	Xpert Xpress	5 (763)	0.98 (0.94, 1.00)	0.98 (0.94, 0.99)	0.79	0.53
Rapid antigen test	Standard Q COVID-19 Ag	13 (8740)	0.72 (0.53, 0.86)	0.99 (0.98, 1.00)	1.49	1.82
	PanBio COVID-19 Ag Rapid test (Abbott)	16 (32,151)	0.72 (0.61, 0.81)	0.99 (0.99, 1.00)	0.98	1.72
	SARS-CoV-2 Rapid Antigen Test (Roche)	7 (6065)	0.77 (0.55, 0.90)	0.99 (0.96, 1.00)	1.33	1.52
	Standard F COVID-19 Ag	5 (6428)	0.65 (0.50, 0.78)	0.98 (0.97, 0.99)	0.67	0.41

The results obtained from the bivariate meta-analysis in a Bayesian framework were in agreement (Appendix 6—see Additional file 4). We summarized the individual study results per test in the forest plots presented in Appendices 7 and 8.

Overall, the accuracy of rapid molecular and rapid antigen tests in identifying true positive and true negative participants was as follows: sensitivity 0.75 [0.70, 0.79] and specificity 0.99 [0.98, 0.99] for antigen tests and sensitivity 0.93 [0.88, 0.96] and specificity 0.98 [0.97, 0.99] for molecular tests (Appendix 9—see Additional file 4).

#### Network meta-analysis

**Fig. 2:**
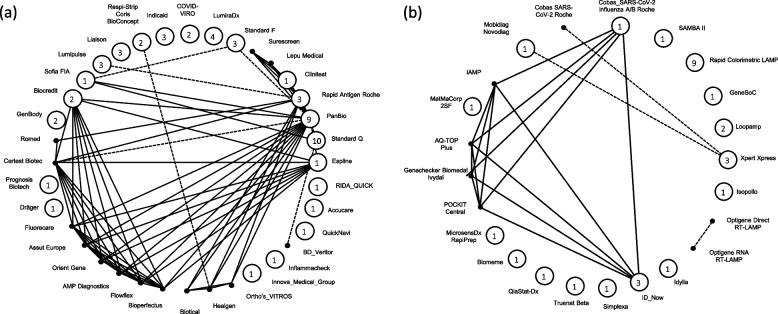
Network geometry of the included studies for (**a)** rapid antigen tests and (**b)** rapid molecular tests. Each vertex represents a different test, and each edge corresponds to at least one study comparing the two tests. Closed circles represent tests compared in single-test studies, and numbers in the circles show the number of single-test studies informing the underlying test. Solid black dots (non-closed circles without an enclosed number) represent tests compared in paired- or multi-test studies only. Dashed lines and solid lines represent paired-test and multiple-test studies, respectively

We conducted one DTA-NMA including all identified rapid antigen tests and one DTA-NMA focused on all identified rapid molecular tests. The network of rapid molecular tests included 26 studies, 10,449 participants and 23 tests, and the network of rapid antigen tests included 68 studies, 104,961 participants and 36 tests, regardless of symptom status or age of participants (Fig. 2). Based on the available data, there was no evidence that the transitivity assumption was challenged in either of the two DTA-NMAs (Appendix 10—see Additional file 4). However, several effect modifiers (e.g., percent of symptomatic and asymptomatic individuals) were not reported in the publications informing the different test comparisons, suggesting that the transitivity assumption could not be assessed appropriately.

Our NMA suggested that the Xpert Xpress rapid molecular test by Cepheid was associated with the highest sensitivity (0.99 [0.83, 1.00]), highest specificity (0.97 [0.69, 1.00]), and highest DOR (3152.16). According to the DOR, this test was followed by the GeneSoC test by Kyorin Pharmaceutical Co. Ltd. (sensitivity: 0.89 [0.33, 1.00]; specificity 0.88 [0.33, 1.00]; DOR: 308.54) and the Truenat Beta CoV test by Molbio Diagnostics (sensitivity: 0.90 [0.31, 1.00]; specificity: 0.86 [0.30, 1.00]; DOR: 170.14). Overall, 15 rapid molecular tests were associated with a sensitivity of  ≥ 0.80 and three rapid molecular tests with a specificity of  ≥ 0.97. Two rapid molecular tests met the minimum performance criteria by WHO and Health Canada: the Xpert Xpress test by Cepheid and the Novodiag COVID-19 test by Mobidiag (sensitivity: 0.80 [0.34, 0.97]; specificity: 0.97 [0.47, 1.00]; DOR: 127.98).

**Fig. 3:**
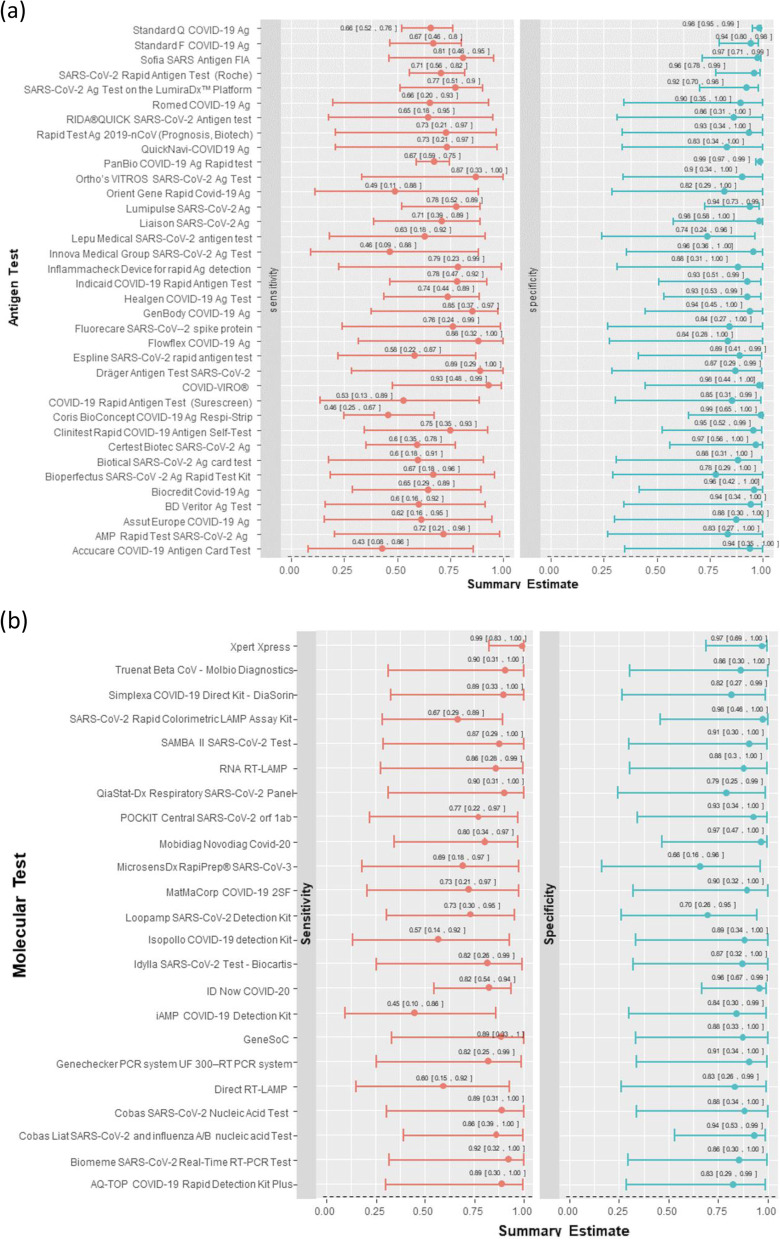
Network meta-analysis interval plots for sensitivity and specificity of **a** rapid antigen tests and **b** rapid molecular tests

Among the rapid antigen tests, our NMA suggested that the COVID-VIRO test by AAZ-LMB was associated with the highest sensitivity (0.93 [0.48, 0.99]), among the highest specificities (0.98 [0.44, 1.00]), and the highest DOR (719.79). According to the DOR, this test was followed by Ortho’s VITROS SARS-CoV-2 Ag Test by Ortho Clinical Diagnostics (sensitivity: 0.87 [0.33, 1.00]; specificity: 0.90 [0.34, 1.00]; DOR: 166.79) and PanBio COVID-19 Ag Rapid test by Abbott (sensitivity: 0.67 [0.59, 0.75]; specificity: 0.99 [0.97, 0.99]; DOR 166.07) (see Fig. 3, Appendix 11—see Additional file 4). Overall, six rapid antigen tests were associated with a sensitivity of  ≥ 0.80 and seven rapid antigen tests with a specificity of  ≥ 0.97. Two rapid antigen tests met the minimum performance criteria by WHO and Health Canada: the COVID-VIRO test by AAZ-LMB and Sofia SARS Antigen FIA test by Quidel (sensitivity: 0.81 [0.46, 0.95]; specificity: 0.97 [0.71, 0.99]; DOR: 143.8).

Heterogeneity estimates are presented in Appendices 12, 13, and 14, for each rapid molecular, rapid antigen, and overall tests in DTA-NMA, separately. Our results show that the overall between-study heterogeneity variance is 0.78 (0.00, 7.25) for sensitivity and 1.58 (0.00, 23.54) for specificity in rapid molecular tests and 1.02 (0.42, 1.91) and 2.17 (0.80, 4.54), respectively, in rapid antigen tests. This suggests that results across studies assessing the underlying tests vary, which can be attributed to the differences across clinical and methodological study characteristics.

#### Subgroup and meta-regression analysis

Our subgroup analyses using the DTA meta-analysis model of rapid antigen tests showed that sensitivity may decrease when:

- Nasopharyngeal is used as a sample type (nasopharyngeal 0.71 [0.66, 0.76] with 71 studies and 34,993 participants)
- Asymptomatic individuals are tested (symptomatic 0.77 [0.58, 0.88] with 17 studies and 27,335 participants; asymptomatic 0.55 [0.32, 0.76] with 10 studies and 8621 participants)

Data on symptomatic vs asymptomatic individuals were not available for rapid molecular tests, but sensitivity in nasopharyngeal samples was 0.91 (0.84, 0.96) with 21 studies and 7916 participants and in combined samples was 0.94 (0.87, 0.98) with 12 studies and 2324 participants (Appendix 15—see Additional file 4). One study (209 participants) assessed saliva sample type in rapid molecular tests and estimated sensitivity 0.96 (0.92, 0.99). Our meta-regression suggested that the relative change in sensitivity increased by 0.50 (0.49, 0.50) with a unit increase in age (Appendix 16—see Additional file 4). In Appendix 17 (see Additional file 4), we present results per age group (< 18 years old, between 18 and 40 years old, and > 65 years old), where two studies including 1262 children suggest that rapid tests are associated with lower sensitivity, irrespective of sample type. However, more evidence is needed to infer on the differences in sensitivity and specificity across age groups. RT-LAMP tests (0.84 [0.67, 0.93]) were less sensitive than rRT-PCR tests (0.97 [0.95, 0.99]) (Appendix 15 and 18—see Additional file 4). These findings are based on a rapid review, where parts of the systematic review process were omitted (e.g., risk of bias assessment) to expedite the time to completion and provide timely advice to decision-makers.

## Discussion

This rapid review and DTA-NMA was conducted to determine the comparative accuracy, in terms of sensitivity and specificity, of rapid antigen and rapid molecular tests separately. Overall, rapid molecular tests were associated with high sensitivity and specificity, while rapid antigen tests were mainly associated with high specificity, as compared with the minimum performance requirements by WHO and Health Canada. Disentangling evidence based on the different tests, only two rapid antigen tests (COVID-VIRO, Sofia SARS Antigen FIA) and two rapid molecular tests (Mobidiag Novodiag COVID-19, Xpert Xpress) met the minimum performance requirements by WHO and Health Canada. However, our DTA-NMA of rapid molecular tests was based on a substantially smaller volume of evidence compared to rapid antigen tests (rapid molecular tests: 27 studies, 10,449 participants, 23 tests vs rapid antigen tests: 68 studies, 104,961participants, 36 tests). Rapid antigen test sensitivity appeared to be higher when nasal or combined samples (more than one of the following samples: nose, throat, mouth, saliva samples) were used, but lower when nasopharyngeal samples were used and when asymptomatic individuals were tested. This may in part be attributed to a greater ability to detect low viral loads in test samples. However, our results should be interpreted with caution since they are based on a rapid review.

Several systematic reviews have been conducted to assess rapid tests for SARS CoV-2 [2–7]. However, our rapid review is the only study combining all available evidence in a single model using DTA-NMA methods. Since it is rarely the case that a single study can sufficiently assess the accuracy of all available tests, an extensive DTA-NMA can best inform practice and policy. Our differences with previously published reviews rest primarily on the more comprehensive analyses regarding the performance of rapid SARS CoV-2 tests. In particular, we conducted a comprehensive literature search in major databases, which was developed, peer-reviewed, and run by experienced librarians [3, 5, 6], used the bivariate meta-analytical modeling to account for correlation between sensitivity and specificity across studies [3], analyzed test estimates coming from fewer than four studies [4, 7] while accounting for sensitivity and specificity correlation, included rapid molecular tests [2–7] in the rapid review, applied the ANOVA DTA-NMA modeling to comparatively evaluate all available tests [2–7] for rapid antigen and rapid molecular tests separately, and ranked tests according to their diagnostic accuracy.

The present findings have not been assessed regarding the study risk of bias, which is likely to impact on the present results. Also, it is not clear what variants of SARS-CoV-2 the participants had during these studies or what the source of specimen (e.g., saliva, nasopharyngeal swab, buccal/ tongue, nasal) was in children. The transitivity assumption assessing the distribution of potential effect modifiers (e.g., percent of symptomatic and asymptomatic individuals) across test comparisons could not be assessed appropriately, since reporting was inadequate in the original study publications. Our literature searches were conducted 7 months ago (up to the time this manuscript was submitted for publication), and in this rapidly moving area, it is possible that we might have missed studies to contribute to the evidence-base. For example, in the included rapid tests, the BTNX test approved for use in Canada in March 2022, which was well after our literature search in September 2021, was not included in our review. Thus, it is likely that there were no published articles of this commercial product at that time. Similar to all systematic reviews, the evidence should be updated regularly.

In the update of this review, a full systematic review should be conducted, where the methodological quality using the QUADAS-2 and QUADAS-C tools [30, 31] should be used, the inclusion of both preprints and publications should be considered, and authors for potentially missing or unclear data should be contacted. Also, the impact of circulating variants, vaccination status, test operator (e.g., nurse, self-testing), who interpreted the results (e.g., nurse, self-testing), and participant age on the accuracy of the individual rapid tests should be evaluated.

## Conclusions

Rapid molecular tests were associated with both high sensitivity and specificity, while rapid antigen tests were mainly associated with high specificity, according to the minimum performance requirements by WHO and Health Canada. The Novodiag COVID-19 by Mobidiag and the Xpert Xpress by Cepheid rapid molecular tests, as well as the COVID-VIRO test by AAZ-LMB and Sofia SARS Antigen FIA by Quidel rapid antigen tests, met the required performance criteria regarding sensitivity and specificity across all identified tests. Overall, rapid antigen test sensitivity was higher when nasal or combined samples (e.g., combinations of nose, throat, mouth, or saliva samples) were used, but lower when nasopharyngeal samples were used, and in those classified as asymptomatic at the time of testing. However, our results should be interpreted with caution, since they are based on a rapid review, and a full systematic review is required to confirm these preliminary results.

## Supplementary Information

**Additional file 1.** PRISMA-DTA Checklist. PRISMA-DTA checklist for COVID-19 DTA-NMA.

**Additional file 2.** PRISMA-DTA for Abstracts Checklist. PRISMA-DTA for Abstracts checklist for COVID-19 DTA-NMA.

**Additional file 3.** PRISMA NMA Checklist. PRISMA NMA checklist for COVID-19 DTA-NMA.

**Additional file 4:** **Appendices.** This document contains supplementary information (Appendices 1–16).

## Data Availability

The data that support the findings of this study are available from the corresponding author upon reasonable request.

## Abbreviations

CIHR: Canadian Institutes of Health Research
DTA-NMA: Diagnostic Test Accuracy Network Meta-Analysis
MAR: Missing at random
NMA: Network meta-analysis
PRISMA: Preferred Reporting Items for Systematic Reviews and Meta-Analyses
PCR: Polymerase chain reaction
RCT: Randomized controlled trial
SARSCoV-2: Severe acute respiratory syndrome coronavirus 2
SPOR: Strategy for Patient Oriented-Research
SPOR EA: Strategy for Patient Oriented-Research Evidence Alliance
WHO: World Health Organization

## References

[1] Priority strategies to optimize testing and screening for COVID-19 in Canada: Report January. 2021. [https://www.canada.ca/en/health-canada/services/drugs-health-products/covid19-industry/medical-devices/testing-screeningadvisory-panel/reports-summaries/priority-strategies.html].

[2] Khalid MF, Selvam K, Jeffry AJN, Salmi MF, Najib MA, Norhayati MN, Aziah I (2022). Performance of rapid antigen tests for COVID-19 diagnosis: a systematic review and meta-analysis. Diagnostics (Basel).

[3] Khandker SS, Nik Hashim NHH, Deris ZZ, Shueb RH, Islam MA (2021). Diagnostic accuracy of rapid antigen test kits for detecting SARS-CoV-2: a systematic review and meta-analysis of 17,171 suspected COVID-19 patients. J Clin Med.

[4] Brummer LE, Katzenschlager S, Gaeddert M, Erdmann C, Schmitz S, Bota M, Grilli M, Larmann J, Weigand MA, Pollock NR (2021). Accuracy of novel antigen rapid diagnostics for SARS-CoV-2: a living systematic review and meta-analysis. PLoS Med.

[5] Tapari A, Braliou GG, Papaefthimiou M, Mavriki H, Kontou PI, Nikolopoulos GK, Bagos PG (2022). Performance of antigen detection tests for SARS-CoV-2: a systematic review and meta-analysis. Diagnostics (Basel).

[6] Xie JW, He Y, Zheng YW, Wang M, Lin Y, Lin LR (2022). Diagnostic accuracy of rapid antigen test for SARS-CoV-2: a systematic review and meta-analysis of 166,943 suspected COVID-19 patients. Microbiol Res.

[7] Dinnes J, Sharma P, Berhane S, van Wyk SS, Nyaaba N, Domen J, Taylor M, Cunningham J, Davenport C, Dittrich S (2022). Rapid, point-of-care antigen tests for diagnosis of SARS-CoV-2 infection. Cochrane Database Syst Rev.

[8] Reitsma JB, Glas AS, Rutjes AW, Scholten RJ, Bossuyt PM, Zwinderman AH (2005). Bivariate analysis of sensitivity and specificity produces informative summary measures in diagnostic reviews. J Clin Epidemiol.

[9] McInnes MDF, Moher D, Thombs BD, McGrath TA, Bossuyt PM, Clifford T, Cohen JF, Deeks JJ, Gatsonis C, the P-DTAG (2018). Preferred Reporting Items for a Systematic Review and Meta-analysis of Diagnostic Test Accuracy Studies: The PRISMA-DTA Statement. JAMA.

[10] Hutton B, Salanti G, Caldwell DM, Chaimani A, Schmid CH, Cameron C, Ioannidis JP, Straus S, Thorlund K, Jansen JP (2015). The PRISMA extension statement for reporting of systematic reviews incorporating network meta-analyses of health care interventions: checklist and explanations. Ann Intern Med.

[11] Moher D, Shamseer L, Clarke M, Ghersi D, Liberati A, Petticrew M, Shekelle P, Stewart LA, Group P-P (2015). Preferred reporting items for systematic review and meta-analysis protocols (PRISMA-P) 2015 statement. Syst Rev.

[12] Tricco AC, Langlois EV, Straus SE, editors. Rapid reviews to strengthen health policy and systems: a practical guide. Geneva: World Health Organization; 2017. Licence: CCBY-NC-SA 3.0 IGO.

[13] McGowan J, Sampson M, Salzwedel DM, Cogo E, Foerster V, Lefebvre C (2016). PRESS Peer Review of Electronic Search Strategies: 2015 Guideline Statement. J Clin Epidemiol.

[14] Coronavirus Test Tracker: Commercially available COVID-19 diagnostics tests [https://www.360dx.com/coronavirus-test-tracker-launched-covid-19-tests].

[15] Dinnes J, Deeks JJ, Berhane S, Taylor M, Adriano A, Davenport C, Dittrich S, Emperador D, Takwoingi Y, Cunningham J (2021). Rapid, point-of-care antigen and molecular-based tests for diagnosis of SARS-CoV-2 infection. Cochrane Database Syst Rev.

[16] SR Systematic Review Tool: St. Michael’s Hospital; 2006 [The Joint Program in Knowledge Translation] [http://knowledgetranslation.ca/sysrev/login.php].

[17] Cochrane (2022). Cochrane Handbook for Systematic Reviews of Diagnostic Test Accuracy.

[18] Veroniki AA, Tsokani S, Agarwal R (2022). Diagnostic test accuracy network meta-analysis methods: a scoping review and empirical assessment. J Clin Epidemiol.

[19] Nyaga VN, Aerts M, Arbyn M (2018). ANOVA model for network meta-analysis of diagnostic test accuracy data. Stat Methods Med Res.

[20] COVID-19 antigen testing devices: Notice on minimum value for sensitivity [https://www.canada.ca/en/health-canada/services/drugs-health-products/covid19-industry/medical-devices/testing/antigen.html].

[21] Antigen-detection in the diagnosis of SARS-CoV-2 infection [https://apps.who.int].

[22] Mavridis D, White IR (2020). Dealing with missing outcome data in meta-analysis. Res Synth Methods.

[23] Veroniki AA, Vasiliadis HS, Higgins JP, Salanti G (2013). Evaluation of inconsistency in networks of interventions. Int J Epidemiol.

[24] Lu G, Ades AE (2006). Assessing evidence inconsistency in mixed treatment comparisons. JASA.

[25] Higgins JP, Jackson D, Barrett JK, Lu G, Ades AE, White IR (2012). Consistency and inconsistency in network meta-analysis: concepts and models for multi-arm studies. Res Synth Methods.

[26] Comprehensive R Archive Network mada: meta-analysis of diagnostic accuracy [https://cranr-projectorg/web/packages/mada/indexhtml].

[27] Lunn D, Spiegelhalter D, Thomas A, Best N (2009). The BUGS project: evolution, critique and future directions. Stat Med.

[28] Plummer M, Best N, Cowles K, Vines K (2006). CODA: convergence diagnosis and output analysis for MCMC. R News.

[29] RStan: the R interface to Stan. R package version 2.21.2 2020 [http://mc-stan.org/].

[30] Whiting PF, Rutjes AW, Westwood ME, Mallett S, Deeks JJ, Reitsma JB, Leeflang MM, Sterne JA, Bossuyt PM (2011). Group Q-: QUADAS-2: a revised tool for the quality assessment of diagnostic accuracy studies. Ann Intern Med.

[31] Yang B, Mallett S, Takwoingi Y, Davenport CF, Hyde CJ, Whiting PF, Deeks JJ, Leeflang MMG, Groupdagger QC, Bossuyt PMM (2021). QUADAS-C: a tool for assessing risk of bias in comparative diagnostic accuracy studies. Ann Intern Med.

