# Supplementary material for: Rapid antigen-based and rapid molecular tests for the detection of SARS-CoV-2: a rapid review with network meta-analysis of diagnostic test accuracy studies
Source: BMC Med. 2023 Mar 29;21:110. doi: 10.1186/s12916-023-02810-0 (PMC10049780; doi:10.1186/s12916-023-02810-0)

**Appendices**

[Appendix 1: Literature search of the rapid review 2](#_Toc106368755)

[Appendix 2. List of Included Studies (n=88) 8](#_Toc106368756)

[Appendix 3. Tests identified in this review of the included studies 15](#_Toc106368757)

[Appendix 4. Proportion of included studies using different types of rapid test 17](#_Toc106368758)

[Appendix 5. Forest plots of studies assessing each rapid antigen test separately 18](#_Toc106368759)

[Appendix 6. Forest plots of studies assessing each rapid molecular test separately 31](#_Toc106368760)

[Appendix 7. ROC space and summary estimate for a) any rapid antigen test, and b) any rapid molecular test included in the DTA bivariate meta-analysis model 39](#_Toc106368761)

[Appendix 8: Transitivity assessment by index test comparison 40](#_Toc106368762)

[Appendix 9. Network meta-analysis results for rapid antigen tests, and rapid molecular tests. 45](#_Toc106368763)

[Appendix 10. Within-test heterogeneity variance for rapid molecular tests in DTA-NMA 47](#_Toc106368764)

[Appendix 11. Within-test heterogeneity variance for rapid antigen tests in DTA-NMA 48](#_Toc106368765)

[Appendix 12. Total between-study heterogeneity variance in DTA-NMA 49](#_Toc106368766)

[Appendix 13. Subgroup analysis results using DTA meta-analysis model by participant type, test sample type, molecular test type, and symptoms 50](#_Toc106368767)

[Appendix 14. Meta-regression analysis results accounting for participant age 51](#_Toc106368768)

[Appendix 15. Study sensitivity and specificity per participant age category 52](#_Toc106368769)

[Appendix 16. Study sensitivity and specificity per category of molecular tests 53](#_Toc106368770)

# Appendix 1: Literature search of the rapid review

Database: Embase Classic+Embase <1947 to 2021 September 10>, Ovid MEDLINE(R) ALL <1946 to September 10, 2021>, EBM Reviews - Cochrane Central Register of Controlled Trials <August 2021>

Search Strategy:

--------------------------------------------------------------------------------

1 COVID-19/

2 SARS-CoV-2/

3 Coronavirus/

4 Betacoronavirus/

5 Coronavirus Infections/

6 (COVID-19 or COVID19).tw,kf.

7 ((coronavirus* or corona virus*) and (hubei or wuhan or beijing or shanghai)).tw,kf.

8 (wuhan adj5 virus*).tw,kf.

9 (2019-nCoV or 19nCoV or 2019nCoV).tw,kf.

10 (nCoV or n-CoV or "CoV 2" or CoV2).tw,kf.

11 (SARS-CoV-2 or SARS-CoV2 or SARSCoV-2 or SARSCoV2 or SARS2 or SARS-2 or severe acute respiratory syndrome coronavirus 2).tw,kf.

12 (2019-novel CoV or Sars-coronavirus2 or Sars-coronavirus-2 or SARS-like coronavirus* or ((novel or new or nouveau) adj2 (CoV or nCoV or covid or coronavirus* or corona virus or Pandemi*2)) or (coronavirus* and pneumonia)).tw,kf.

13 (novel coronavirus* or novel corona virus* or novel CoV).tw,kf.

14 ((coronavirus* or corona virus*) adj2 "2019").tw,kf.

15 ((coronavirus* or corona virus*) adj2 "19").tw,kf.

16 ("coronavirus 2" or "corona virus 2").tw,kf.

17 (OC43 or NL63 or 229E or HKU1 or HCoV* or Sars-coronavirus*).tw,kf.

18 COVID-19.rx,px,ox. or severe acute respiratory syndrome coronavirus 2.os.

19 (coronavirus* or corona virus*).ti.

20 COVID.ti.

21 ("B.1.1.7" or "B.1.351" or "B.1.617" or "B.1.427" or "B.1.429" or "B.1.621").tw,kf,rx,px,ox.

22 ("P.1" and (Brazil* or variant?)).tw,kf,rx,px,ox.

23 (((alpha or beta or delta or eta or gamma or iota or kappa or lambda or mu) adj3 variant?) and (coronavirus* or corona virus* or covid*)).tw,kf.

24 or/1-23 [COVID-19]

25 COVID-19 Testing/

26 Point-of-Care Testing/

27 ((fast or quick* or rapid* or point-of-care or POC or portab* or bedside or bed side or near patient? or same-day) adj5 (test* or assay* or immunoassay* or immuno-assay* or detect* or diagnos* or screen*)).tw,kf.

28 POCT.tw,kf.

29 (antigen* adj5 (test* or assay* or immunoassay* or immuno-assay* or detect* or diagnos* or screen*)).tw,kf.

30 (lateral flow adj5 (test* or assay* or immunoassay* or immuno-assay* or detect* or diagnos* or identif* or screen*)).tw,kf.

31 ((immunochromatographic* or immuno-chromatographic* or immuno-chromato-graphic* or immunochromato-graphic*) adj5 (test* or assay* or immunoassay* or immuno-assay* or detect* or diagnos* or identif* or screen*)).tw,kf.

32 (RADT or RADTs).tw,kf.

33 (RDT and diagnos* and test*).tw,kf.

34 ((LFD or LFDs of LFIA or LFIAs or LFT or LFTs) and (lateral and flow) and (test* or assay* or immunoassay* or immuno-assay*)).tw,kf.

35 ((rapid adj (polymerase chain reaction? or PCR)) and (test* or assay* or immunoassay* or immuno-assay* or detect* or diagnos* or identif* or screen*)).tw,kf.

36 (nucleic acid amplification test* or NAAT).tw,kf.

37 (((isothermal or iso-thermal) adj3 amplification*) or LAMP).tw,kf.

38 or/25-37 [RAPID TESTING]

39 24 and 38 [COVID-19 - RAPID TESTING]

40 exp Animals/ not Humans/

41 39 not 40 [ANIMAL-ONLY REMOVED]

42 (comment or editorial or news or newspaper article).pt.

43 41 not 42 [OPINION PIECES REMOVED]

44 (controlled clinical trial or randomized controlled trial or pragmatic clinical trial or equivalence trial).pt.

45 "Clinical Trials as Topic"/

46 exp "Controlled Clinical Trials as Topic"/

47 (randomi#ed or randomi#ation? or randomly or RCT or placebo*).tw,kf.

48 ((singl* or doubl* or trebl* or tripl*) adj (mask* or blind* or dumm*)).tw,kf.

49 trial.ti.

50 or/44-49 [RCT FILTER]

51 43 and 50 [COVID-19 - RAPID TESTING - RCTs]

52 controlled clinical trial.pt.

53 Controlled Clinical Trial/ or Controlled Clinical Trials as Topic/

54 (control* adj2 trial).tw,kf.

55 Non-Randomized Controlled Trials as Topic/

56 (nonrandom* or non-random* or quasi-random* or quasi-experiment*).tw,kf.

57 (nRCT or non-RCT).tw,kf.

58 Controlled Before-After Studies/

59 (control* adj3 ("before and after" or "before after")).tw,kf.

60 Interrupted Time Series Analysis/

61 time series.tw,kf.

62 (pre- adj3 post-).tw,kf.

63 (pretest adj3 posttest).tw,kf.

64 Historically Controlled Study/

65 (control* adj2 study).tw,kf.

66 Control Groups/

67 (control* adj2 group?).tw,kf.

68 trial.ti.

69 or/52-68

70 43 and 69 [nRCTs]

71 exp Cohort Studies/

72 cohort$1.tw,kf.

73 Retrospective Studies/

74 (longitudinal* or prospective* or retrospective*).tw,kf.

75 ((followup or follow-up) adj (study or studies)).tw,kf.

76 Observational study.pt.

77 (observation$2 adj (study or studies)).tw,kf.

78 ((population or population-based) adj (study or studies or analys#s)).tw,kf.

79 ((multidimensional or multi-dimensional) adj (study or studies)).tw,kf.

80 Comparative Study.pt.

81 ((comparative or comparison) adj (study or studies)).tw,kf.

82 exp Case-Control Studies/

83 ((case-control* or case-based or case-comparison) adj (study or studies)).tw,kf.

84 Cross-Sectional Studies/

85 (crosssection* or cross section*).tw,kf.

86 or/71-85

87 43 and 86 [OBSERVATIONAL STUDIES]

88 51 or 70 or 87 [ALL STUDY DESIGNS]

89 88 use medall [MEDLINE RECORDS]

90 coronavirus disease 2019/

91 severe acute respiratory syndrome coronavirus 2/

92 Coronavirinae/

93 Betacoronavirus/

94 coronavirus infection/

95 (COVID-19 or COVID19).tw,kw.

96 ((coronavirus* or corona virus*) and (hubei or wuhan or beijing or shanghai)).tw,kw.

97 (wuhan adj5 virus*).tw,kw.

98 (2019-nCoV or 19nCoV or 2019nCoV).tw,kw.

99 (nCoV or n-CoV or "CoV 2" or CoV2).tw,kw.

100 (SARS-CoV-2 or SARS-CoV2 or SARSCoV-2 or SARSCoV2 or SARS2 or SARS-2 or severe acute respiratory syndrome coronavirus 2).tw,kw.

101 (2019-novel CoV or Sars-coronavirus2 or Sars-coronavirus-2 or SARS-like coronavirus* or ((novel or new or nouveau) adj2 (CoV or nCoV or covid or coronavirus* or corona virus or Pandemi*2)) or (coronavirus* and pneumonia)).tw,kw.

102 (novel coronavirus* or novel corona virus* or novel CoV).tw,kw.

103 ((coronavirus* or corona virus*) adj2 "2019").tw,kw.

104 ((coronavirus* or corona virus*) adj2 "19").tw,kw.

105 ("coronavirus 2" or "corona virus 2").tw,kw.

106 (OC43 or NL63 or 229E or HKU1 or HCoV* or Sars-coronavirus*).tw,kw.

107 (coronavirus* or corona virus*).ti.

108 COVID.ti.

109 ("B.1.1.7" or "B.1.351" or "B.1.617" or "B.1.427" or "B.1.429" or "B.1.621").tw,kw.

110 ("P.1" and (Brazil* or variant?)).tw,kw.

111 (((alpha or beta or delta or eta or gamma or iota or kappa or lambda or mu) adj3 variant?) and (coronavirus* or corona virus* or covid*)).tw,kw.

112 or/90-111 [COVID-19]

113 SARS coronavirus 2 test kit/ and (antigen or lateral flow or fast or quick* or rapid* or point-of-care or POC or portab* or bedside or bed side or near patient? or same-day).tw,kw.

114 rapid test/

115 point of care testing/

116 ((fast or quick* or rapid* or point-of-care or POC or portab* or bedside or bed side or near patient? or same-day) adj5 (test* or assay* or immunoassay* or immuno-assay* or detect* or diagnos* or screen*)).tw,kw.

117 POCT.tw,kw.

118 (antigen* adj5 (test* or assay* or immunoassay* or immuno-assay* or detect* or diagnos* or screen*)).tw,kw.

119 (lateral flow adj5 (test* or assay* or immunoassay* or immuno-assay* or detect* or diagnos* or identif* or screen*)).tw,kw.

120 ((immunochromatographic* or immuno-chromatographic* or immuno-chromato-graphic* or immunochromato-graphic*) adj5 (test* or assay* or immunoassay* or immuno-assay* or detect* or diagnos* or identif* or screen*)).tw,kw.

121 (RADT or RADTs).tw,kw.

122 (RDT and diagnos* and test*).tw,kw.

123 ((LFD or LFDs of LFIA or LFIAs or LFT or LFTs) and (lateral and flow) and (test* or assay* or immunoassay* or immuno-assay*)).tw,kw.

124 ((rapid adj (polymerase chain reaction? or PCR)) and (test* or assay* or immunoassay* or immuno-assay* or detect* or diagnos* or identif* or screen*)).tw,kw.

125 (nucleic acid amplification test* or NAAT).tw,kw.

126 (((isothermal or iso-thermal) adj3 amplification*) or LAMP).tw,kw.

127 or/113-126 [RAPID TESTING]

128 112 and 127 [COVID-19 - RAPID TESTING]

129 exp animal/ or exp animal experimentation/ or exp animal model/ or exp animal experiment/ or nonhuman/ or exp vertebrate/

130 exp human/ or exp human experimentation/ or exp human experiment/

131 129 not 130

132 128 not 131 [ANIMAL-ONLY REMOVED]

133 editorial.pt.

134 132 not 133 [OPINION PIECES REMOVED]

135 exp randomized controlled trial/ or controlled clinical trial/

136 clinical trial/

137 exp "controlled clinical trial (topic)"/

138 (randomi#ed or randomi#ation? or randomly or RCT or placebo*).tw,kw.

139 ((singl* or doubl* or trebl* or tripl*) adj (mask* or blind* or dumm*)).tw,kw.

140 trial.ti.

141 or/135-140 [RCT FILTER]

142 134 and 141 [COVID-19 - RAPID TESTING - RCTs]

143 exp controlled clinical trial/

144 exp "controlled clinical trial (topic)"/

145 (control* adj2 trial*).kw,tw.

146 (nonrandom* or non-random* or quasi-random* or quasi-experiment*).kw,tw.

147 (nRCT or non-RCT).kw,tw.

148 (control* adj3 ("before and after" or "before after")).kw,tw.

149 time series analysis/

150 time series.kw,tw.

151 pretest posttest control group design/

152 (pre- adj3 post-).kw,tw.

153 (pretest adj3 posttest).kw,tw.

154 controlled study/

155 (control* adj2 stud$3).kw,tw.

156 control group/

157 (control* adj2 group?).kw,tw.

158 or/143-157

159 134 and 158 [COVID-19 - RAPID TESTING - nRCTS]

160 cohort analysis/

161 cohort?.tw,kw.

162 retrospective study/

163 longitudinal study/

164 prospective study/

165 (longitudinal* or prospective* or retrospective*).tw,kw.

166 follow up/

167 ((followup or follow-up) adj (study or studies)).tw,kw.

168 observational study/

169 (observation$2 adj (study or studies)).tw,kw.

170 population research/

171 ((population or population-based) adj (study or studies or analys#s)).tw,kw.

172 ((multidimensional or multi-dimensional) adj (study or studies)).tw,kw.

173 exp comparative study/

174 ((comparative or comparison) adj (study or studies)).tw,kw.

175 exp case control study/

176 ((case-control* or case-based or case-comparison) adj (study or studies)).tw,kw.

177 cross-sectional study/

178 (crosssection* or cross section*).tw,kw.

179 major clinical study/

180 or/160-179

181 134 and 180 [COVID-19 - RAPID TESTING - OBSERVATIONAL STUDIES]

182 142 or 159 or 181 [ALL STUDY DESIGNS]

183 182 use emczd [EMBASE RECORDS]

184 COVID-19/

185 SARS-CoV-2/

186 Coronavirus/

187 Betacoronavirus/

188 Coronavirus Infections/

189 (COVID-19 or COVID19).ti,ab,kw.

190 ((coronavirus* or corona virus*) and (hubei or wuhan or beijing or shanghai)).ti,ab,kw.

191 (wuhan adj5 virus*).ti,ab,kw.

192 (2019-nCoV or 19nCoV or 2019nCoV).ti,ab,kw.

193 (nCoV or n-CoV or "CoV 2" or CoV2).ti,ab,kw.

194 (SARS-CoV-2 or SARS-CoV2 or SARSCoV-2 or SARSCoV2 or SARS2 or SARS-2 or severe acute respiratory syndrome coronavirus 2).ti,ab,kw.

195 (2019-novel CoV or Sars-coronavirus2 or Sars-coronavirus-2 or SARS-like coronavirus* or ((novel or new or nouveau) adj2 (CoV or nCoV or covid or coronavirus* or corona virus or Pandemi*2)) or (coronavirus* and pneumonia)).ti,ab,kw.

196 (novel coronavirus* or novel corona virus* or novel CoV).ti,ab,kw.

197 ((coronavirus* or corona virus*) adj2 "2019").ti,ab,kw.

198 ((coronavirus* or corona virus*) adj2 "19").ti,ab,kw.

199 ("coronavirus 2" or "corona virus 2").ti,ab,kw.

200 (OC43 or NL63 or 229E or HKU1 or HCoV* or Sars-coronavirus*).ti,ab,kw

201 (COVID-19 or severe acute respiratory syndrome coronavirus 2).os.

202 (coronavirus* or corona virus*).ti.

203 COVID.ti.

204 ("B.1.1.7" or "B.1.351" or "B.1.617" or "B.1.427" or "B.1.429" or "B.1.621").ti,ab,kw.

205 ("P.1" and (Brazil* or variant?)).ti,ab,kw.

206 (((alpha or beta or delta or eta or gamma or iota or kappa or lambda or mu) adj3 variant?) and (coronavirus* or corona virus* or covid*)).ti,ab,kw.

207 or/184-206 [COVID-19

208 COVID-19 Testing/

209 Point-of-Care Testing/

210 ((fast or quick* or rapid* or point-of-care or POC or portab* or bedside or bed side or near patient? or same-day) adj5 (test* or assay* or immunoassay* or immuno-assay* or detect* or diagnos* or screen*)).ti,ab,kw.

211 POCT.ti,ab,kw.

212 (antigen* adj5 (test* or assay* or immunoassay* or immuno-assay* or detect* or diagnos* or screen*)).ti,ab,kw.

213 (lateral flow adj5 (test* or assay* or immunoassay* or immuno-assay* or detect* or diagnos* or identif* or screen*)).ti,ab,kw.

214 ((immunochromatographic* or immuno-chromatographic* or immuno-chromato-graphic* or immunochromato-graphic*) adj5 (test* or assay* or immunoassay* or immuno-assay* or detect* or diagnos* or identif* or screen*)).ti,ab,kw.

215 (RADT or RADTs).ti,ab,kw.

216 (RDT and diagnos* and test*).ti,ab,kw.

217 ((LFD or LFDs of LFIA or LFIAs or LFT or LFTs) and (lateral and flow) and (test* or assay* or immunoassay* or immuno-assay*)).ti,ab,kw.

218 ((rapid adj (polymerase chain reaction? or PCR)) and (test* or assay* or immunoassay* or immuno-assay* or detect* or diagnos* or identif* or screen*)).ti,ab,kw.

219 (nucleic acid amplification test* or NAAT).ti,ab,kw.

220 (((isothermal or iso-thermal) adj3 amplification*) or LAMP).ti,ab,kw.

221 or/208-220 [RAPID TESTING]

222 207 and 221 [COVID-19 - RAPID TESTING]

223 222 use cctr [CENTRAL RECORDS - RCTs]

224 89 or 183 or 223 [ALL DATABASES]

225 51 use medall [MEDLINE RCTS]

226 142 use emczd [EMBASE RCTS]

227 223 use cctr [CENTRAL RCTS]

228 or/225-227 [RCTs - ALL DATBASES]

229 remove duplicates from 228 (746) [TOTAL UNIQUE RCTS]

230 229 use medall [MEDLINE UNIQUE RCTs]

231 229 use emczd [EMBASE UNIQUE RCTs]

232 229 use cctr [CENTRAL UNIQUE RCTs]

233 70 use medall [MEDLINE NRCTS]

234 87 use medall [MEDLINE OBSERVATIONAL STUDIES]

235 159 use emczd [EMBASE NRCTS]

236 181 use emczd [EMBASE OBSERVATIONAL STUDIES]

237 or/233-236 [nRCTS & OBSERVATIONAL STUDIES]

238 remove duplicates from 237

239 238 not 229 [OVERLAP WITH RCTS REMOVED] [TOTAL UNIQUE NRCTS/OBSERV STUDIES]

240 239 use medall [MEDLINE UNIQUE NRCTS/OBSERVATIONAL STUDIES]

241 239 use emczd [EMBASE UNIQUE nRCTS/OBSERVATIONAL STUDIES]

***************************

# Appendix 2. List of Included Studies (n=88)

1. Abusrewil Z, Alhudiri IM, Kaal HH, El Meshri SE, Ebrahim FO, Dalyoum T, et al. Time scale performance of rapid antigen testing for SARS-CoV-2: Evaluation of 10 rapid antigen assays. J Med Virol. 2021;93(12):6512-8.
2. Allan-Blitz LT, Klausner JD. A Real-World Comparison of SARS-CoV-2 Rapid Antigen Testing versus PCR Testing in Florida. J Clin Microbiol. 2021;59(10):e0110721.
3. Anastasiou OE, Holtkamp C, Schafer M, Schon F, Eis-Hubinger AM, Krumbholz A. Fast Detection of SARS-CoV-2 RNA Directly from Respiratory Samples Using a Loop-Mediated Isothermal Amplification (LAMP) Test. Viruses. 2021;13(5).
4. Aoki K, Nagasawa T, Ishii Y, Yagi S, Kashiwagi K, Miyazaki T, et al. Evaluation of clinical utility of novel coronavirus antigen detection reagent, Espline(R) SARS-CoV-2. J Infect Chemother. 2021;27(2):319-22.
5. Baro B, Rodo P, Ouchi D, Bordoy AE, Saya Amaro EN, Salsench SV, et al. Performance characteristics of five antigen-detecting rapid diagnostic test (Ag-RDT) for SARS-CoV-2 asymptomatic infection: a head-to-head benchmark comparison. J Infect. 2021;82(6):269-75.
6. Basawarajappa SG, Rangaiah A, Padukone S, Yadav PD, Gupta N, Shankar SM. Performance evaluation of Truenat Beta CoV & Truenat SARS-CoV-2 point-of-care assays for coronavirus disease 2019. Indian J Med Res. 2021;153(1 & 2):144-50.
7. Basu A, Zinger T, Inglima K, Woo KM, Atie O, Yurasits L, et al. Performance of Abbott ID Now COVID-19 Rapid Nucleic Acid Amplification Test Using Nasopharyngeal Swabs Transported in Viral Transport Media and Dry Nasal Swabs in a New York City Academic Institution. J Clin Microbiol. 2020;58(8).
8. Berger A, Nsoga MTN, Perez-Rodriguez FJ, Aad YA, Sattonnet-Roche P, Gayet-Ageron A, et al. Diagnostic accuracy of two commercial SARS-CoV-2 antigen-detecting rapid tests at the point of care in community-based testing centers. PLoS One. 2021;16(3):e0248921.
9. Bianco G, Boattini M, Barbui AM, Scozzari G, Riccardini F, Coggiola M, et al. Evaluation of an antigen-based test for hospital point-of-care diagnosis of SARS-CoV-2 infection. J Clin Virol. 2021;139:104838.
10. Bordi L, Piralla A, Lalle E, Giardina F, Colavita F, Tallarita M, et al. Rapid and sensitive detection of SARS-CoV-2 RNA using the Simplexa COVID-19 direct assay. J Clin Virol. 2020;128:104416.
11. Bouam A, Vincent JJ, Le Glass E, Almeras L, Levy PY, Tissot-Dupont H, et al. Rapid Isothermal Amplification for the Buccal Detection SARS-CoV-2 in the Context of Out-Patient COVID-19 Screening. J Clin Med. 2021;10(12).
12. Boum Y, Fai KN, Nikolay B, Mboringong AB, Bebell LM, Ndifon M, et al. Performance and operational feasibility of antigen and antibody rapid diagnostic tests for COVID-19 in symptomatic and asymptomatic patients in Cameroon: a clinical, prospective, diagnostic accuracy study. Lancet Infect Dis. 2021;21(8):1089-96.
13. Burnes LE, Clark ST, Sheldrake E, Faheem A, Poon BP, Christie-Holmes N, et al. One swab, two tests: Validation of dual SARS-CoV-2 testing on the Abbott ID NOW. J Clin Virol. 2021;141:104896.
14. Caputo V, Bax C, Colantoni L, Peconi C, Termine A, Fabrizio C, et al. Comparative analysis of antigen and molecular tests for the detection of Sars-CoV-2 and related variants: A study on 4266 samples. Int J Infect Dis. 2021;108:187-9.
15. Caruana G, Lebrun LL, Aebischer O, Opota O, Urbano L, de Rham M, et al. The dark side of SARS-CoV-2 rapid antigen testing: screening asymptomatic patients. New Microbes New Infect. 2021;42:100899.
16. Cassuto NG, Gravier A, Colin M, Theillay A, Pires-Roteira D, Pallay S, et al. Evaluation of a SARS-CoV-2 antigen-detecting rapid diagnostic test as a self-test: Diagnostic performance and usability. J Med Virol. 2021;93(12):6686-92.
17. Cento V, Renica S, Matarazzo E, Antonello M, Colagrossi L, Di Ruscio F, et al. Frontline Screening for SARS-CoV-2 Infection at Emergency Department Admission by Third Generation Rapid Antigen Test: Can We Spare RT-qPCR? Viruses. 2021;13(5).
18. Cerutti F, Burdino E, Milia MG, Allice T, Gregori G, Bruzzone B, et al. Urgent need of rapid tests for SARS CoV-2 antigen detection: Evaluation of the SD-Biosensor antigen test for SARS-CoV-2. J Clin Virol. 2020;132:104654.
19. Chaimayo C, Kaewnaphan B, Tanlieng N, Athipanyasilp N, Sirijatuphat R, Chayakulkeeree M, et al. Rapid SARS-CoV-2 antigen detection assay in comparison with real-time RT-PCR assay for laboratory diagnosis of COVID-19 in Thailand. Virol J. 2020;17(1):177.
20. Chiu RYT, Kojima N, Mosley GL, Cheng KK, Pereira DY, Brobeck M, et al. Evaluation of the INDICAID COVID-19 Rapid Antigen Test in Symptomatic Populations and Asymptomatic Community Testing. Microbiol Spectr. 2021;9(1):e0034221.
21. Ciotti M, Maurici M, Pieri M, Andreoni M, Bernardini S. Performance of a rapid antigen test in the diagnosis of SARS-CoV-2 infection. J Med Virol. 2021;93(5):2988-91.
22. Collier DA, Assennato SM, Warne B, Sithole N, Sharrocks K, Ritchie A, et al. Point of Care Nucleic Acid Testing for SARS-CoV-2 in Hospitalized Patients: A Clinical Validation Trial and Implementation Study. Cell Rep Med. 2020;1(5):100062.
23. Courtellemont L, Guinard J, Guillaume C, Giache S, Rzepecki V, Seve A, et al. High performance of a novel antigen detection test on nasopharyngeal specimens for diagnosing SARS-CoV-2 infection. J Med Virol. 2021;93(5):3152-7.
24. Craney A, Petrik D, Suhku A, Qiu Y, Racine-Brzostek S, Rennert H, et al. Performance Evaluation of the MatMaCorp COVID-19 2SF Assay for the Detection of SARS-CoV-2 from Nasopharyngeal Swabs. Microbiol Spectr. 2021;9(1):e0008321.
25. De Luca C, Gragnano G, Conticelli F, Cennamo M, Pisapia P, Terracciano D, et al. Evaluation of a fully closed real time PCR platform for the detection of SARS-CoV-2 in nasopharyngeal swabs: a pilot study. J Clin Pathol. 2021.
26. Dust K, Hedley A, Nichol K, Stein D, Adam H, Karlowsky JA, et al. Comparison of commercial assays and laboratory developed tests for detection of SARS-CoV-2. J Virol Methods. 2020;285:113970.
27. Favresse J, Gillot C, Oliveira M, Cadrobbi J, Elsen M, Eucher C, et al. Head-to-Head Comparison of Rapid and Automated Antigen Detection Tests for the Diagnosis of SARS-CoV-2 Infection. J Clin Med. 2021;10(2).
28. Fiedler M, Holtkamp C, Dittmer U, Anastasiou OE. Performance of the LIAISON((R)) SARS-CoV-2 Antigen Assay vs. SARS-CoV-2-RT-PCR. Pathogens. 2021;10(6).
29. Fowler VL, Armson B, Gonzales JL, Wise EL, Howson ELA, Vincent-Mistiaen Z, et al. A highly effective reverse-transcription loop-mediated isothermal amplification (RT-LAMP) assay for the rapid detection of SARS-CoV-2 infection. J Infect. 2021;82(1):117-25.
30. Freire-Paspuel B, Garcia-Bereguiain MA. Low clinical performance of the Isopollo COVID-19 detection kit (M Monitor, South Korea) for RT-LAMP SARS-CoV-2 diagnosis: A call for action against low quality products for developing countries. Int J Infect Dis. 2021;104:303-5.
31. Garcia-Finana M, Hughes DM, Cheyne CP, Burnside G, Stockbridge M, Fowler TA, et al. Performance of the Innova SARS-CoV-2 antigen rapid lateral flow test in the Liverpool asymptomatic testing pilot: population based cohort study. BMJ. 2021;374:n1637.
32. Gonzalez-Donapetry P, Garcia-Clemente P, Bloise I, Garcia-Sanchez C, Sanchez Castellano MA, Romero MP, et al. Think of the Children: Evaluation of SARS-CoV-2 Rapid Antigen Test in Pediatric Population. Pediatr Infect Dis J. 2021;40(5):385-8.
33. Gupta A, Khurana S, Das R, Srigyan D, Singh A, Mittal A, et al. Rapid chromatographic immunoassay-based evaluation of COVID-19: A cross-sectional, diagnostic test accuracy study & its implications for COVID-19 management in India. Indian J Med Res. 2021;153(1 & 2):126-31.
34. Hansen G, Marino J, Wang ZX, Beavis KG, Rodrigo J, Labog K, et al. Clinical Performance of the Point-of-Care cobas Liat for Detection of SARS-CoV-2 in 20 Minutes: a Multicenter Study. J Clin Microbiol. 2021;59(2).
35. Hartard C, Berger S, Josse T, Schvoerer E, Jeulin H. Performance evaluation of an automated SARS-CoV-2 Ag test for the diagnosis of COVID-19 infection on nasopharyngeal swabs. Clin Chem Lab Med. 2021;59(12):2003-9.
36. Hirotsu Y, Maejima M, Shibusawa M, Nagakubo Y, Hosaka K, Amemiya K, et al. Comparison of automated SARS-CoV-2 antigen test for COVID-19 infection with quantitative RT-PCR using 313 nasopharyngeal swabs, including from seven serially followed patients. Int J Infect Dis. 2020;99:397-402.
37. Hirotsu Y, Sugiura H, Maejima M, Hayakawa M, Mochizuki H, Tsutsui T, et al. Comparison of Roche and Lumipulse quantitative SARS-CoV-2 antigen test performance using automated systems for the diagnosis of COVID-19. Int J Infect Dis. 2021;108:263-9.
38. Igloi Z, Velzing J, van Beek J, van de Vijver D, Aron G, Ensing R, et al. Clinical Evaluation of Roche SD Biosensor Rapid Antigen Test for SARS-CoV-2 in Municipal Health Service Testing Site, the Netherlands. Emerg Infect Dis. 2021;27(5):1323-9.
39. Inaba M, Higashimoto Y, Toyama Y, Horiguchi T, Hibino M, Iwata M, et al. Diagnostic accuracy of LAMP versus PCR over the course of SARS-CoV-2 infection. Int J Infect Dis. 2021;107:195-200.
40. Jaaskelainen AE, Ahava MJ, Jokela P, Szirovicza L, Pohjala S, Vapalahti O, et al. Evaluation of three rapid lateral flow antigen detection tests for the diagnosis of SARS-CoV-2 infection. J Clin Virol. 2021;137:104785.
41. James AE, Gulley T, Kothari A, Holder K, Garner K, Patil N. Performance of the BinaxNOW coronavirus disease 2019 (COVID-19) Antigen Card test relative to the severe acute respiratory coronavirus virus 2 (SARS-CoV-2) real-time reverse transcriptase polymerase chain reaction (rRT-PCR) assay among symptomatic and asymptomatic healthcare employees. Infect Control Hosp Epidemiol. 2022;43(1):99-101.
42. Jegerlehner S, Suter-Riniker F, Jent P, Bittel P, Nagler M. Diagnostic accuracy of a SARS-CoV-2 rapid antigen test in real-life clinical settings. Int J Infect Dis. 2021;109:118-22.
43. Jokela P, Jaaskelainen AE, Jarva H, Holma T, Ahava MJ, Mannonen L, et al. SARS-CoV-2 sample-to-answer nucleic acid testing in a tertiary care emergency department: evaluation and utility. J Clin Virol. 2020;131:104614.
44. Kahn M, Schuierer L, Bartenschlager C, Zellmer S, Frey R, Freitag M, et al. Performance of antigen testing for diagnosis of COVID-19: a direct comparison of a lateral flow device to nucleic acid amplification based tests. BMC Infect Dis. 2021;21(1):798.
45. Kim D, Lee J, Bal J, Seo SK, Chong CK, Lee JH, et al. Development and Clinical Evaluation of an Immunochromatography-Based Rapid Antigen Test (GenBody COVAG025) for COVID-19 Diagnosis. Viruses. 2021;13(5).
46. Koeleman JGM, Brand H, de Man SJ, Ong DSY. Clinical evaluation of rapid point-of-care antigen tests for diagnosis of SARS-CoV-2 infection. Eur J Clin Microbiol Infect Dis. 2021;40(9):1975-81.
47. Korenkov M, Poopalasingam N, Madler M, Vanshylla K, Eggeling R, Wirtz M, et al. Evaluation of a Rapid Antigen Test To Detect SARS-CoV-2 Infection and Identify Potentially Infectious Individuals. J Clin Microbiol. 2021;59(9):e0089621.
48. Kruger LJ, Gaeddert M, Tobian F, Lainati F, Gottschalk C, Klein JAF, et al. The Abbott PanBio WHO emergency use listed, rapid, antigen-detecting point-of-care diagnostic test for SARS-CoV-2-Evaluation of the accuracy and ease-of-use. PLoS One. 2021;16(5):e0247918.
49. Kruger LJ, Klein JAF, Tobian F, Gaeddert M, Lainati F, Klemm S, et al. Evaluation of accuracy, exclusivity, limit-of-detection and ease-of-use of LumiraDx: An antigen-detecting point-of-care device for SARS-CoV-2. Infection. 2022;50(2):395-406.
50. Kyritsi M, Vontas A, Voulgaridi I, Matziri A, Komnos A, Babalis D, et al. Rapid Test Ag 2019-nCoV (PROGNOSIS, BIOTECH, Larissa, Greece); Performance Evaluation in Hospital Setting with Real Time RT-PCR. Int J Environ Res Public Health. 2021;18(17).
51. Landaas ET, Storm ML, Tollanes MC, Barlinn R, Kran AB, Bragstad K, et al. Diagnostic performance of a SARS-CoV-2 rapid antigen test in a large, Norwegian cohort. J Clin Virol. 2021;137:104789.
52. Leli C, Di Matteo L, Gotta F, Cornaglia E, Vay D, Megna I, et al. Performance of a SARS-CoV-2 antigen rapid immunoassay in patients admitted to the emergency department. Int J Infect Dis. 2021;110:135-40.
53. L'Huillier AG, Lacour M, Sadiku D, Gadiri MA, De Siebenthal L, Schibler M, et al. Diagnostic Accuracy of SARS-CoV-2 Rapid Antigen Detection Testing in Symptomatic and Asymptomatic Children in the Clinical Setting. J Clin Microbiol. 2021;59(9):e0099121.
54. Loeffelholz MJ, Alland D, Butler-Wu SM, Pandey U, Perno CF, Nava A, et al. Multicenter Evaluation of the Cepheid Xpert Xpress SARS-CoV-2 Test. J Clin Microbiol. 2020;58(8).
55. Mahmoud SA, Ganesan S, Ibrahim E, Thakre B, Teddy JG, Raheja P, et al. Evaluation of six different rapid methods for nucleic acid detection of SARS-COV-2 virus. J Med Virol. 2021;93(9):5538-43.
56. Maniscalco M, Ambrosino P, Ciullo A, Fuschillo S, Valente V, Gaudiosi C, et al. A Rapid Antigen Detection Test to Diagnose SARS-CoV-2 Infection Using Exhaled Breath Condensate by A Modified Inflammacheck((R)) Device. Sensors (Basel). 2021;21(17).
57. Martin-Sanchez V, Fernandez-Villa T, Carvajal Uruena A, Rivero Rodriguez A, Reguero Celada S, Sanchez Antolin G, et al. Role of Rapid Antigen Testing in Population-Based SARS-CoV-2 Screening. J Clin Med. 2021;10(17).
58. McKay SL, Tobolowsky FA, Moritz ED, Hatfield KM, Bhatnagar A, LaVoie SP, et al. Performance Evaluation of Serial SARS-CoV-2 Rapid Antigen Testing During a Nursing Home Outbreak. Ann Intern Med. 2021;174(7):945-51.
59. Menchinelli G, De Angelis G, Cacaci M, Liotti FM, Candelli M, Palucci I, et al. SARS-CoV-2 Antigen Detection to Expand Testing Capacity for COVID-19: Results from a Hospital Emergency Department Testing Site. Diagnostics (Basel). 2021;11(7).
60. Merino P, Guinea J, Munoz-Gallego I, Gonzalez-Donapetry P, Galan JC, Antona N, et al. Multicenter evaluation of the Panbio COVID-19 rapid antigen-detection test for the diagnosis of SARS-CoV-2 infection. Clin Microbiol Infect. 2021.
61. Merino-Amador P, Gonzalez-Donapetry P, Dominguez-Fernandez M, Gonzalez-Romo F, Sanchez-Castellano MA, Seoane-Estevez A, et al. Clinitest rapid COVID-19 antigen test for the diagnosis of SARS-CoV-2 infection: A multicenter evaluation study. J Clin Virol. 2021;143:104961.
62. Minami K, Masutani R, Suzuki Y, Kubota M, Osaka N, Nakanishi T, et al. Evaluation of SARS-CoV-2 RNA quantification by RT-LAMP compared to RT-qPCR. J Infect Chemother. 2021;27(7):1068-71.
63. Nomoto H, Yamamoto K, Yamada G, Suzuki M, Kinoshita N, Takasaki J, et al. Time-course evaluation of the quantitative antigen test for severe acute respiratory syndrome coronavirus 2: The potential contribution to alleviating isolation of COVID-19 patients. J Infect Chemother. 2021;27(11):1669-73.
64. Norz D, Olearo F, Perisic S, Bauer MF, Riester E, Schneider T, et al. Multicenter Evaluation of a Fully Automated High-Throughput SARS-CoV-2 Antigen Immunoassay. Infect Dis Ther. 2021;10(4):2371-9.
65. Oh SM, Jeong H, Chang E, Choe PG, Kang CK, Park WB, et al. Clinical Application of the Standard Q COVID-19 Ag Test for the Detection of SARS-CoV-2 Infection. J Korean Med Sci. 2021;36(14):e101.
66. Osmanodja B, Budde K, Zickler D, Naik MG, Hofmann J, Gertler M, et al. Accuracy of a Novel SARS-CoV-2 Antigen-Detecting Rapid Diagnostic Test from Standardized Self-Collected Anterior Nasal Swabs. J Clin Med. 2021;10(10).
67. Osterdahl MF, Lee KA, Lochlainn MN, Wilson S, Douthwaite S, Horsfall R, et al. Detecting SARS-CoV-2 at point of care: preliminary data comparing loop-mediated isothermal amplification (LAMP) to polymerase chain reaction (PCR). BMC Infect Dis. 2020;20(1):783.
68. Pena-Rodriguez M, Viera-Segura O, Garcia-Chagollan M, Zepeda-Nuno JS, Munoz-Valle JF, Mora-Mora J, et al. Performance evaluation of a lateral flow assay for nasopharyngeal antigen detection for SARS-CoV-2 diagnosis. J Clin Lab Anal. 2021;35(5):e23745.
69. Perez-Garcia F, Romanyk J, Gomez-Herruz P, Arroyo T, Perez-Tanoira R, Linares M, et al. Diagnostic performance of CerTest and Panbio antigen rapid diagnostic tests to diagnose SARS-CoV-2 infection. J Clin Virol. 2021;137:104781.
70. Perez-Garcia F, Romanyk J, Moya Gutierrez H, Labrador Ballestero A, Perez Ranz I, Gonzalez Arroyo J, et al. Comparative evaluation of Panbio and SD Biosensor antigen rapid diagnostic tests for COVID-19 diagnosis. J Med Virol. 2021;93(9):5650-4.
71. Porte L, Legarraga P, Iruretagoyena M, Vollrath V, Pizarro G, Munita J, et al. Evaluation of two fluorescence immunoassays for the rapid detection of SARS-CoV-2 antigen-new tool to detect infective COVID-19 patients. PeerJ. 2021;9:e10801.
72. Ristic M, Nikolic N, Cabarkapa V, Turkulov V, Petrovic V. Validation of the STANDARD Q COVID-19 antigen test in Vojvodina, Serbia. PLoS One. 2021;16(2):e0247606.
73. Rus KR UT. Shortening turnaround time for high-priority patients during the COVID-19 epidemic: evaluation of the Xpert Xpress SARS-CoV-2 test. Slovenian Medical Journal. 2020;14(11-12):614-25.
74. Schellenberg JJ, Ormond M, Keynan Y. Extraction-free RT-LAMP to detect SARS-CoV-2 is less sensitive but highly specific compared to standard RT-PCR in 101 samples. J Clin Virol. 2021;136:104764.
75. Schuit E, Veldhuijzen IK, Venekamp RP, van den Bijllaardt W, Pas SD, Lodder EB, et al. Diagnostic accuracy of rapid antigen tests in asymptomatic and presymptomatic close contacts of individuals with confirmed SARS-CoV-2 infection: cross sectional study. BMJ. 2021;374:n1676.
76. Scohy A, Anantharajah A, Bodeus M, Kabamba-Mukadi B, Verroken A, Rodriguez-Villalobos H. Low performance of rapid antigen detection test as frontline testing for COVID-19 diagnosis. J Clin Virol. 2020;129:104455.
77. Seynaeve Y, Heylen J, Fontaine C, Maclot F, Meex C, Diep AN, et al. Evaluation of Two Rapid Antigenic Tests for the Detection of SARS-CoV-2 in Nasopharyngeal Swabs. J Clin Med. 2021;10(13).
78. Shrestha B, Neupane AK, Pant S, Shrestha A, Bastola A, Rajbhandari B, et al. Sensitivity and Specificity of Lateral Flow Antigen Test Kits for COVID-19 in Asymptomatic Population of Quarantine Centre of Province 3. Kathmandu Univ Med J (KUMJ). 2020;18(70):36-9.
79. Smith RD, Johnson JK, Clay C, Girio-Herrera L, Stevens D, Abraham M, et al. Clinical evaluation of Sofia Rapid Antigen Assay for detection of severe acute respiratory syndrome coronavirus 2 (SARS-CoV-2) among emergency department to hospital admissions. Infect Control Hosp Epidemiol. 2021:1-6.
80. Stevens B, Hogan CA, Sahoo MK, Huang C, Garamani N, Zehnder J, et al. Comparison of a Point-of-Care Assay and a High-Complexity Assay for Detection of SARS-CoV-2 RNA. J Appl Lab Med. 2020;5(6):1307-12.
81. Takeuchi Y, Akashi Y, Kato D, Kuwahara M, Muramatsu S, Ueda A, et al. The evaluation of a newly developed antigen test (QuickNavi-COVID19 Ag) for SARS-CoV-2: A prospective observational study in Japan. J Infect Chemother. 2021;27(6):890-4.
82. Thakur P, Saxena S, Manchanda V, Rana N, Goel R, Arora R. Utility of Antigen-Based Rapid Diagnostic Test for Detection of SARS-CoV-2 Virus in Routine Hospital Settings. Lab Med. 2021;52(6):e154-e8.
83. Toptan T, Eckermann L, Pfeiffer AE, Hoehl S, Ciesek S, Drosten C, et al. Evaluation of a SARS-CoV-2 rapid antigen test: Potential to help reduce community spread? J Clin Virol. 2021;135:104713.
84. Van der Moeren N, Zwart VF, Goderski G, Rijkers GT, van den Bijllaardt W, Veenemans J, et al. Performance of the Diasorin SARS-CoV-2 antigen detection assay on the LIAISON XL. J Clin Virol. 2021;141:104909.
85. Villaverde S, Dominguez-Rodriguez S, Sabrido G, Perez-Jorge C, Plata M, Romero MP, et al. Diagnostic Accuracy of the Panbio Severe Acute Respiratory Syndrome Coronavirus 2 Antigen Rapid Test Compared with Reverse-Transcriptase Polymerase Chain Reaction Testing of Nasopharyngeal Samples in the Pediatric Population. J Pediatr. 2021;232:287-9 e4.
86. Visseaux B, Le Hingrat Q, Collin G, Bouzid D, Lebourgeois S, Le Pluart D, et al. Evaluation of the QIAstat-Dx Respiratory SARS-CoV-2 Panel, the First Rapid Multiplex PCR Commercial Assay for SARS-CoV-2 Detection. J Clin Microbiol. 2020;58(8).
87. Watanabe R, Asai S, Kakizoe H, Saeki H, Masukawa A, Miyazawa M, et al. Evaluation of the basic assay performance of the GeneSoc(R) rapid PCR testing system for detection of severe acute respiratory syndrome coronavirus 2. PLoS One. 2021;16(3):e0248397.
88. Zowawi HM, Alenazi TH, AlOmaim WS, Wazzan A, Alsufayan A, Hasanain RA, et al. Portable RT-PCR System: a Rapid and Scalable Diagnostic Tool for COVID-19 Testing. J Clin Microbiol. 2021;59(5).

# Appendix 3. Tests identified in this review of the included studies

| **Test name** | **Manufacturer** |
| --- | --- |
| **Rapid Antigen Tests (n = 36)** | |
| Accucare COVID-19 Antigen Card Test | Lab-Care Diagnostics (India) Pvt. Ltd |
| AMP Rapid Test SARS-CoV-2 Ag | AMP Diagnostics |
| Assut Europe COVID-19 Ag | Assut Europe |
| BD Veritor Ag Test | Beckton Dickinson |
| Biocredit Covid-19 Ag | RapiGEN Inc |
| Bioperfectus SARS-CoV -2 Ag Rapid Test Kit | Bioperfectus technologies |
| Biotical SARS-CoV-2 Ag card test | Biotical Health |
| Certest Biotec SARS-CoV-2 Ag | Certest Biotec |
| Clinitest Rapid COVID-19 Antigen Self-Test | Siemens Healthineers |
| Coris BioConcept COVID-19 Ag Respi-Strip | Coris BioConcept |
| COVID-19 Rapid Antigen Test (Surescreen) | Surescreen |
| COVID-VIRO® | AAZ-LMB |
| Dräger Antigen Test SARS-CoV-2 | Dräger |
| Espline SARS-CoV-2 rapid antigen test | Fujirebio Inc., Japan |
| Flowflex COVID-19 Ag | Acon Laboratories |
| Fluorecare SARS-CoV--2 spike protein | Shenzhen Microprofit Biotech Co |
| GenBody COVID-19 Ag | Meridian Bioscience |
| Healgen COVID-19 Ag Test | Healgen Scientific |
| Indicaid COVID-19 Rapid Antigen Test | Phase Scientifc International |
| Inflammacheck Device for rapid Ag detection | Exhalation Medical Technology |
| Innova Medical Group SARS-CoV-2 Ag Test | Innova |
| Lepu Medical SARS-CoV-2 antigen test | Lepu Medical |
| Liaison SARS-CoV-2 Ag | DiaSorin |
| Lumipulse SARS-CoV-2 Ag | Fujirebio |
| Orient Gene Rapid Covid-19 Ag | Orient Gene/Healgen Biotech |
| Ortho’s VITROS SARS-CoV-2 Ag Test | Ortho Clinical Diagnostics |
| PanBio COVID-19 Ag Rapid test | Abbott |
| QuickNavi-COVID19 Ag | Otsuka Pharmaceutical Co., Ltd. |
| Rapid Test Ag 2019-nCoV (Prognosis, Biotech) | Prognosis, Biotech |
| RIDA®QUICK SARS-CoV-2 Antigen test | R-Biopharm AG |
| Romed COVID-19 Ag | Romed |
| SARS-CoV-2 Ag Test on the LumiraDx™ Platform | LumiraDx |
| SARS-CoV-2 Rapid Antigen Test (Roche) | Roche |
| Sofia SARS Antigen FIA | Quidel |
| Standard F COVID-19 Ag | SD-Biosensor |
| Standard Q COVID-19 Ag | SD-Biosensor |
| **Rapid Molecular Tests (n = 23)** | |
| AQ-TOP COVID-19 Rapid Detection Kit Plus | Seasun Biomaterials |
| Biomeme SARS-CoV-2 Real-Time RT-PCR Test | Biomeme |
| Cobas Liat SARS-CoV-2 and influenza A/B nucleic acid test | Roche |
| Cobas SARS-CoV-2 Nucleic Acid Test | Roche |
| Direct RT-LAMP | Optigene Ltd. |
| Genechecker PCR system‐UF 300–RT PCR system | Biomedal Ivydal |
| GeneSoC | Kyorin Pharmaceutical Co. Ltd |
| iAMP COVID-19 Detection Kit | Atila BioSystems |
| ID Now COVID-19 | Abbott |
| Idylla SARS-CoV-2 Test | Biocartis |
| Isopollo COVID-19 detection kit | M Monitor |
| Loopamp SARS-CoV-2 Detection Kit | Eiken Chemical, Tokyo, Japan |
| MatMaCorp COVID-19 2SF | MatMaCorp |
| MicrosensDx RapiPrep® SARS-CoV-2 | MicrosensDx |
| Mobidiag Novodiag Covid-19 | Mobidiag |
| POCKIT Central SARS-CoV-2 (ORF1 ab) | GeneReach Biotechnology Corp |
| QiaStat-Dx Respiratory SARS-CoV-2 Panel | Qiagen |
| RNA RT-LAMP | Optigene |
| SAMBA II SARS-CoV-2 Test | DRW: Diagnostics for the Real World |
| SARS-CoV-2 Rapid Colorimetric LAMP Assay Kit | New England BioLabs |
| Simplexa COVID-19 Direct Kit | DiaSorin |
| Truenat Beta CoV | Molbio Diagnostics |
| Xpert Xpress | Cepheid |

# Appendix 4. Proportion of included studies using different types of rapid test


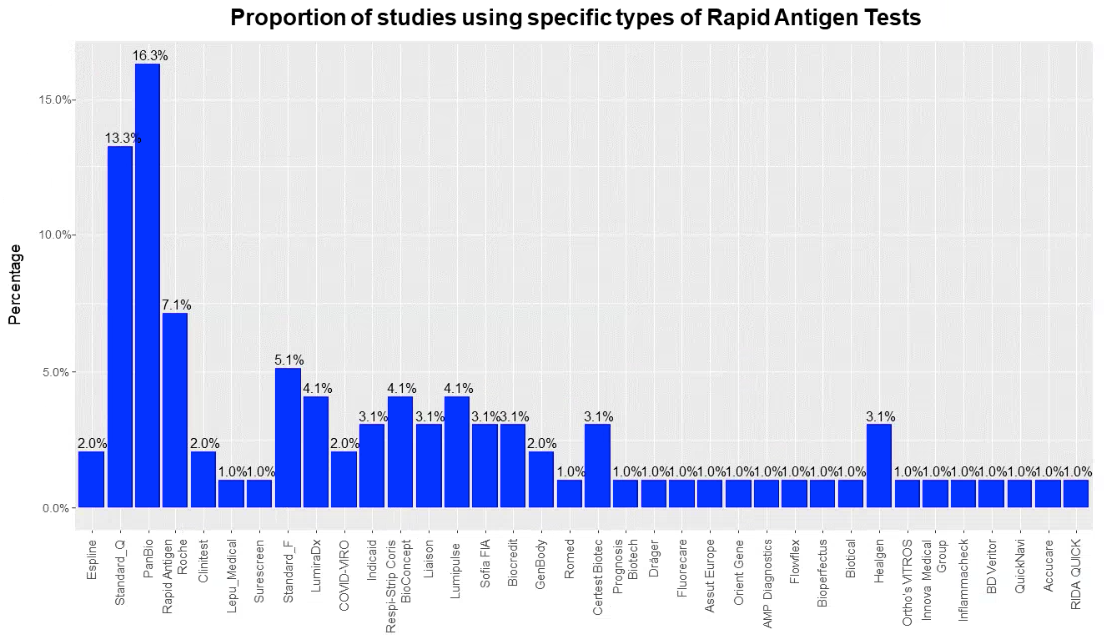


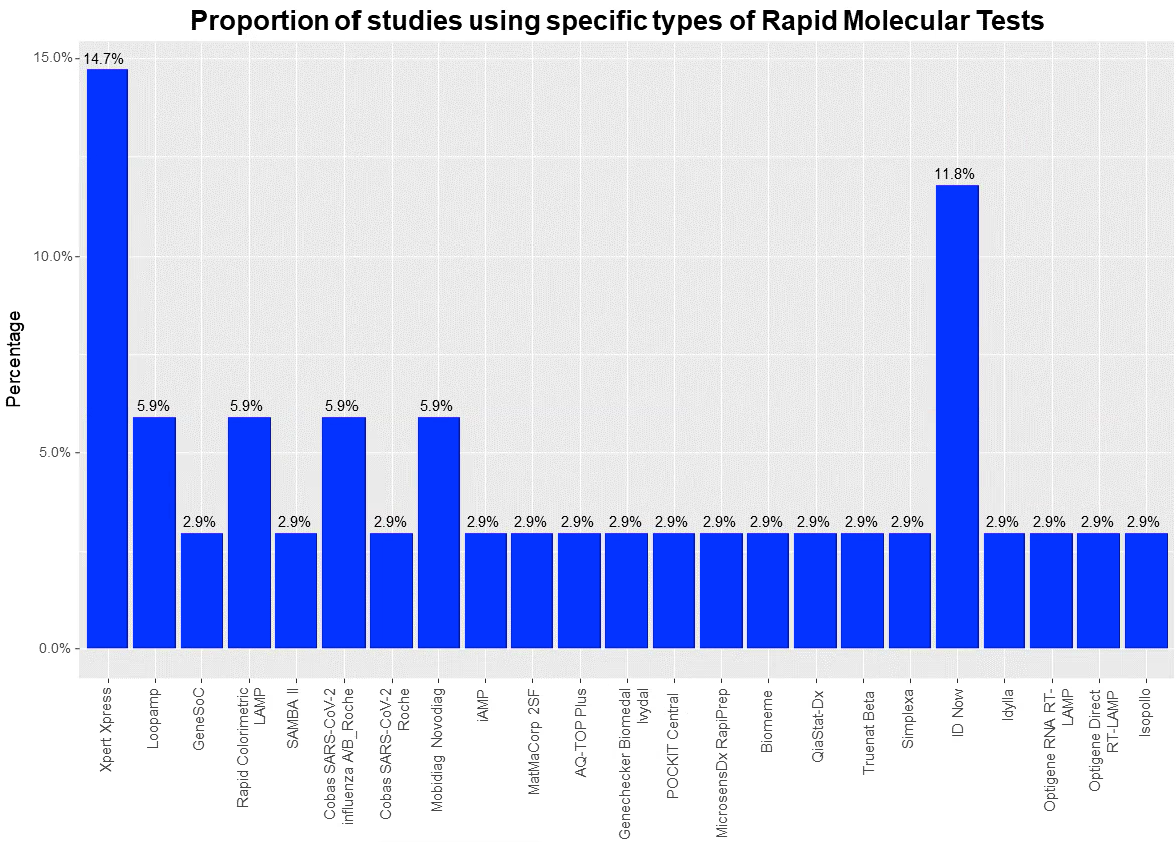


# Appendix 5. Forest plots of studies assessing each rapid antigen test separately


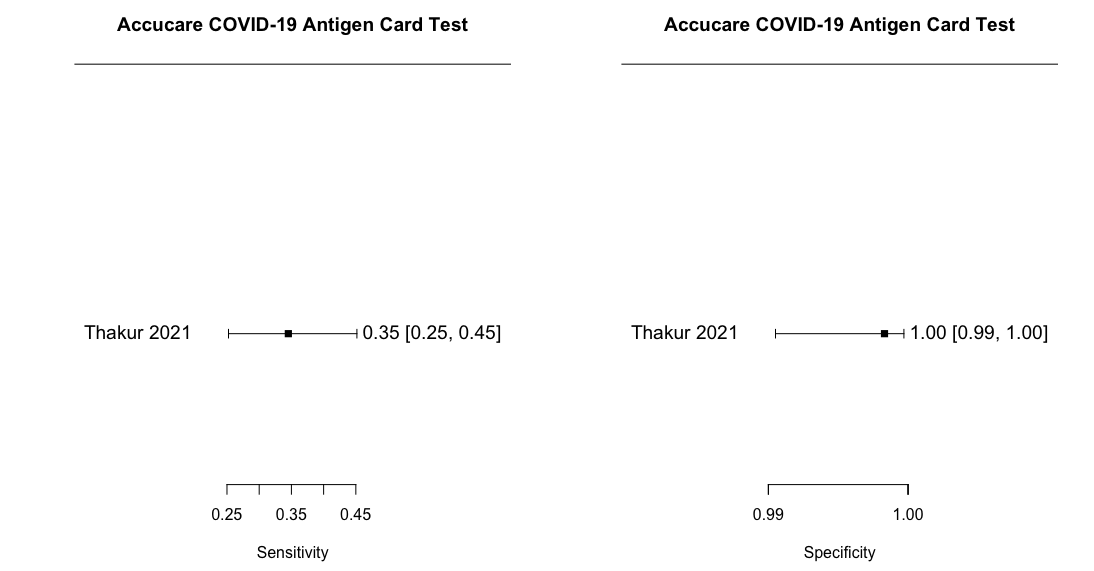


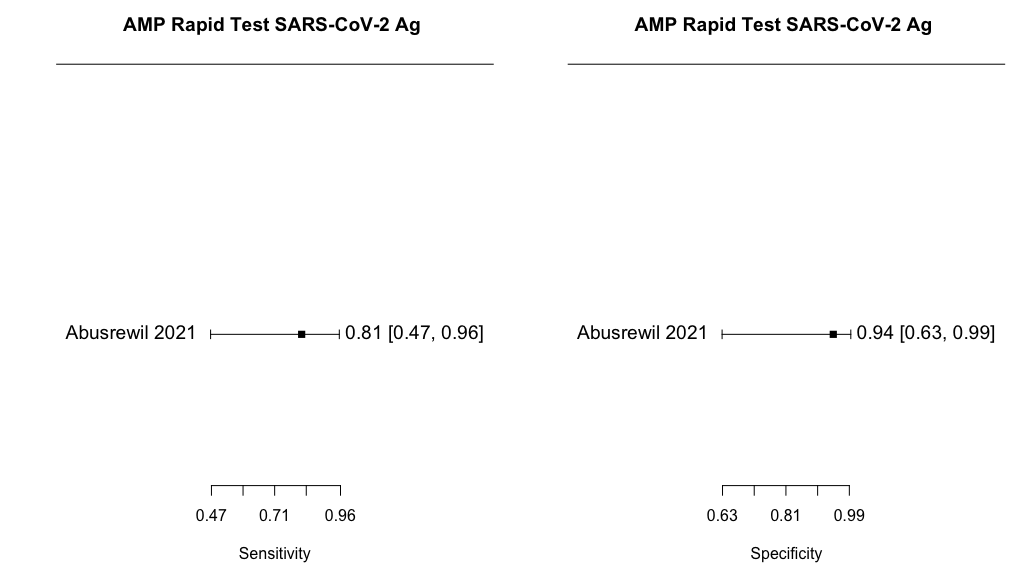


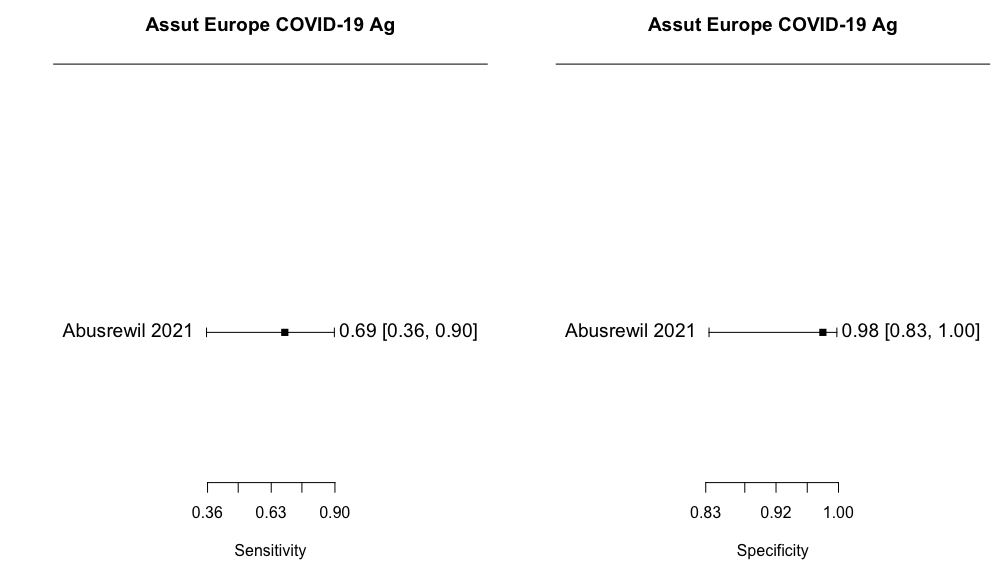


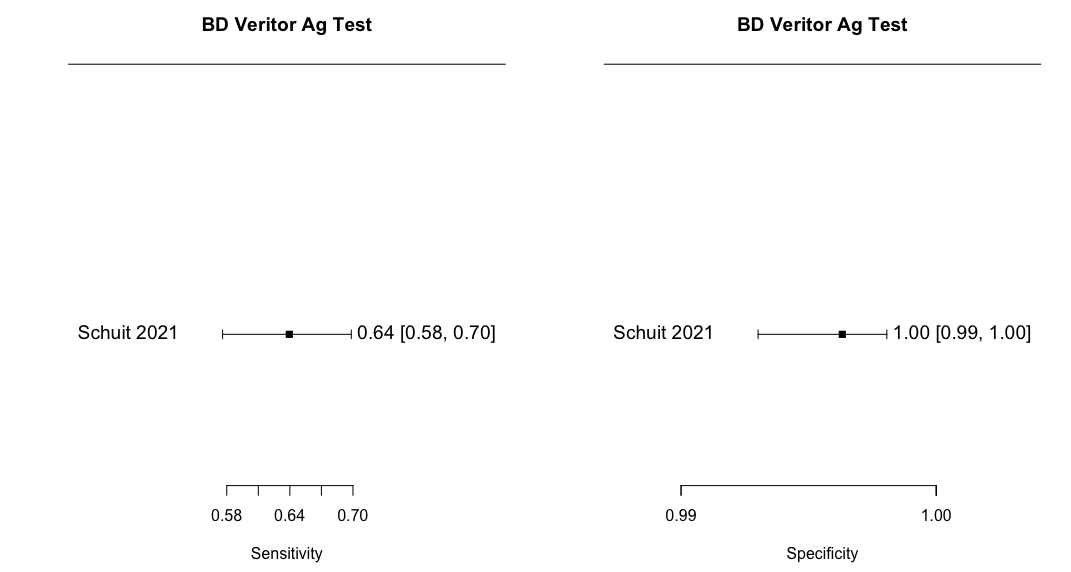


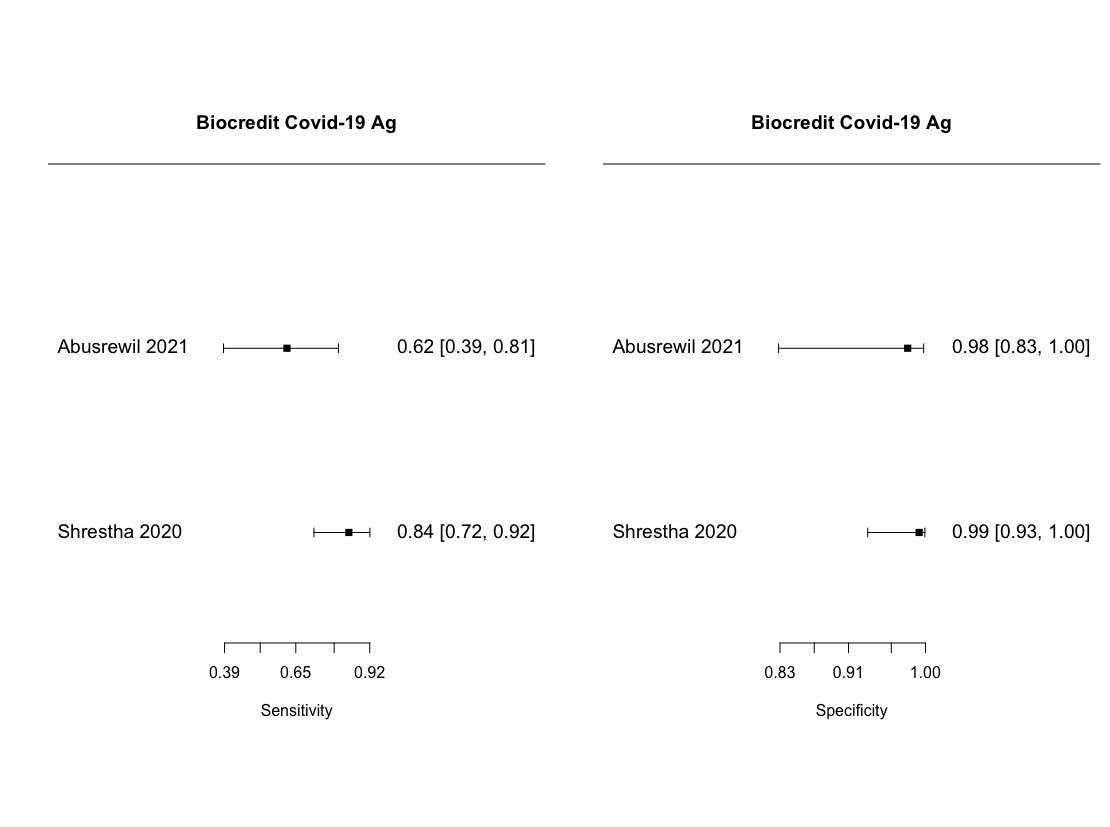


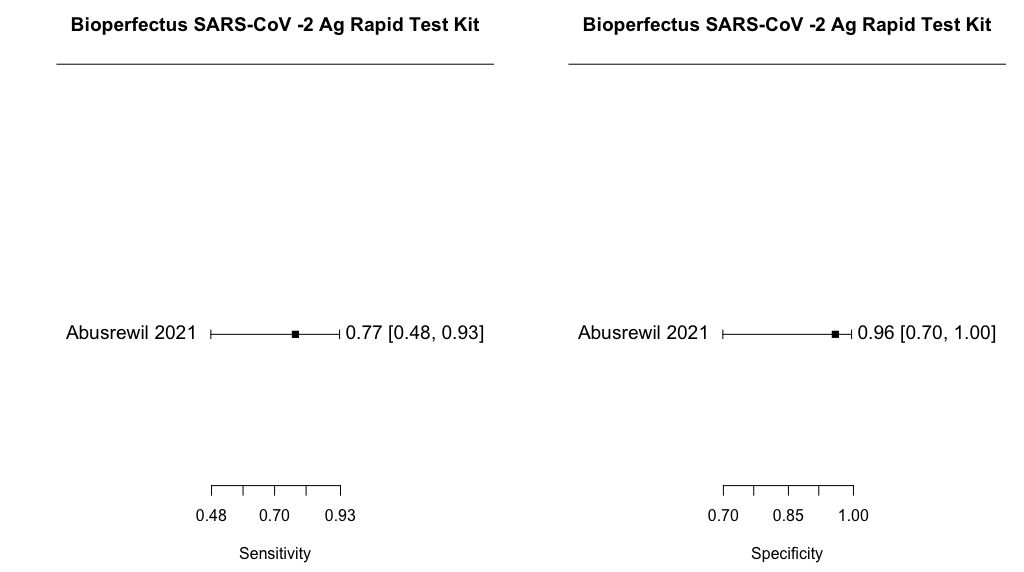


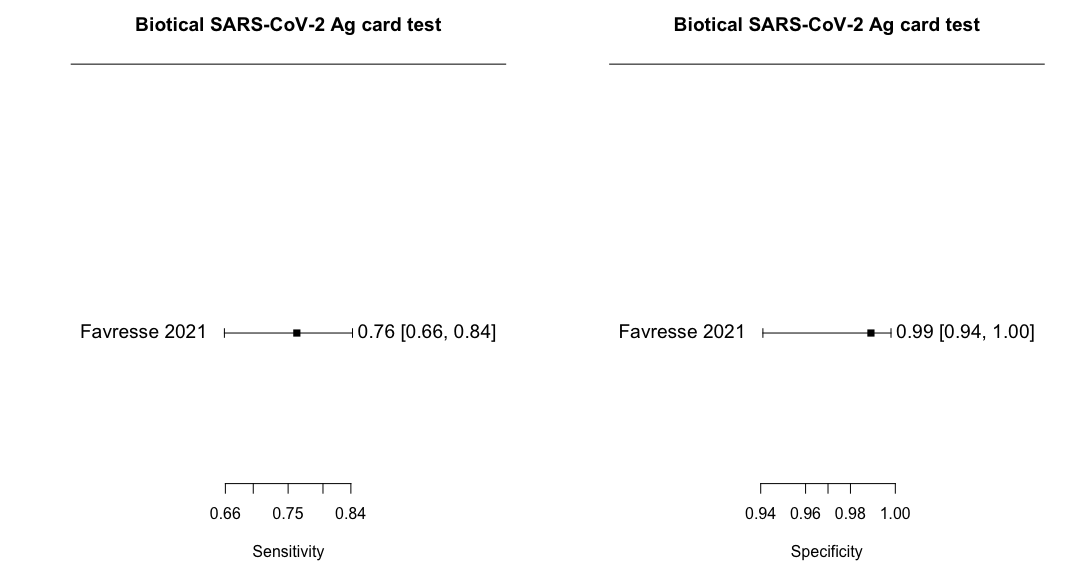


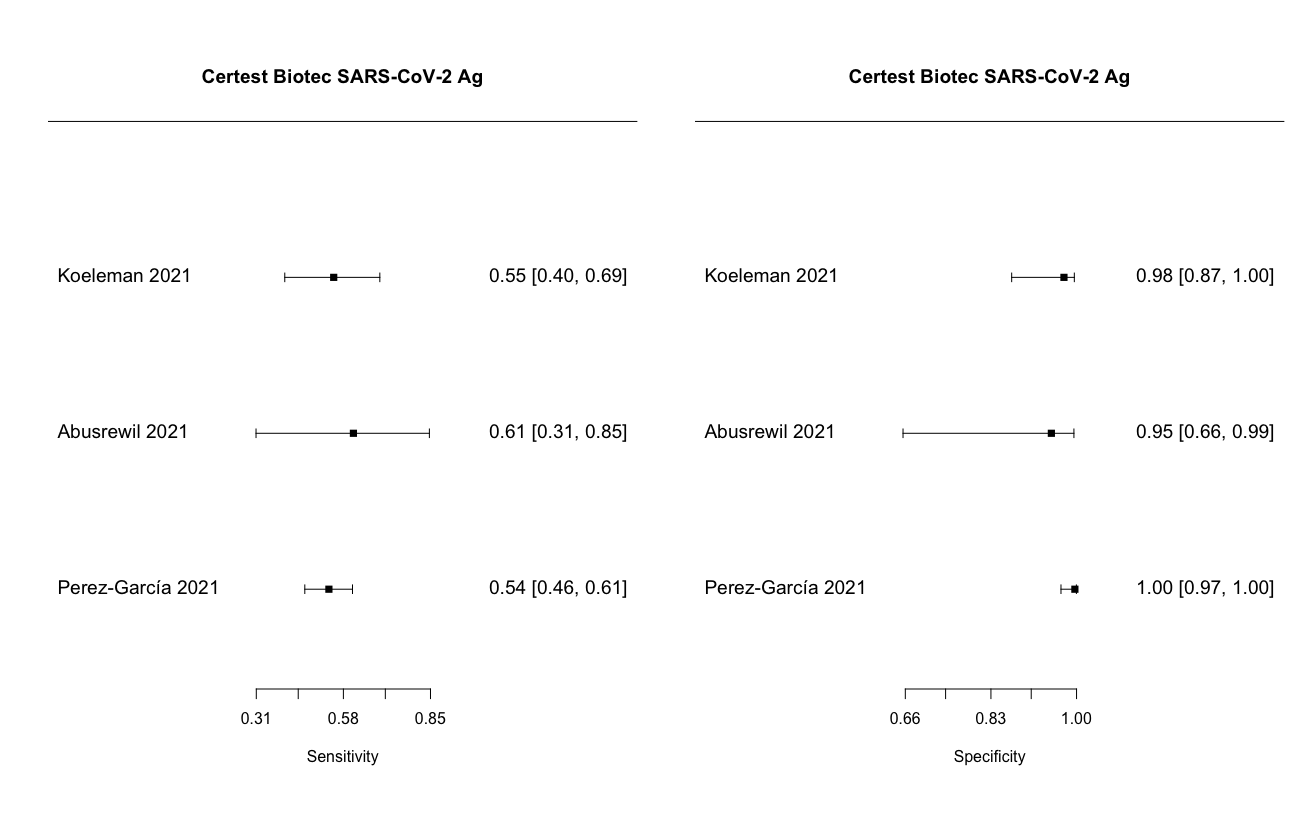


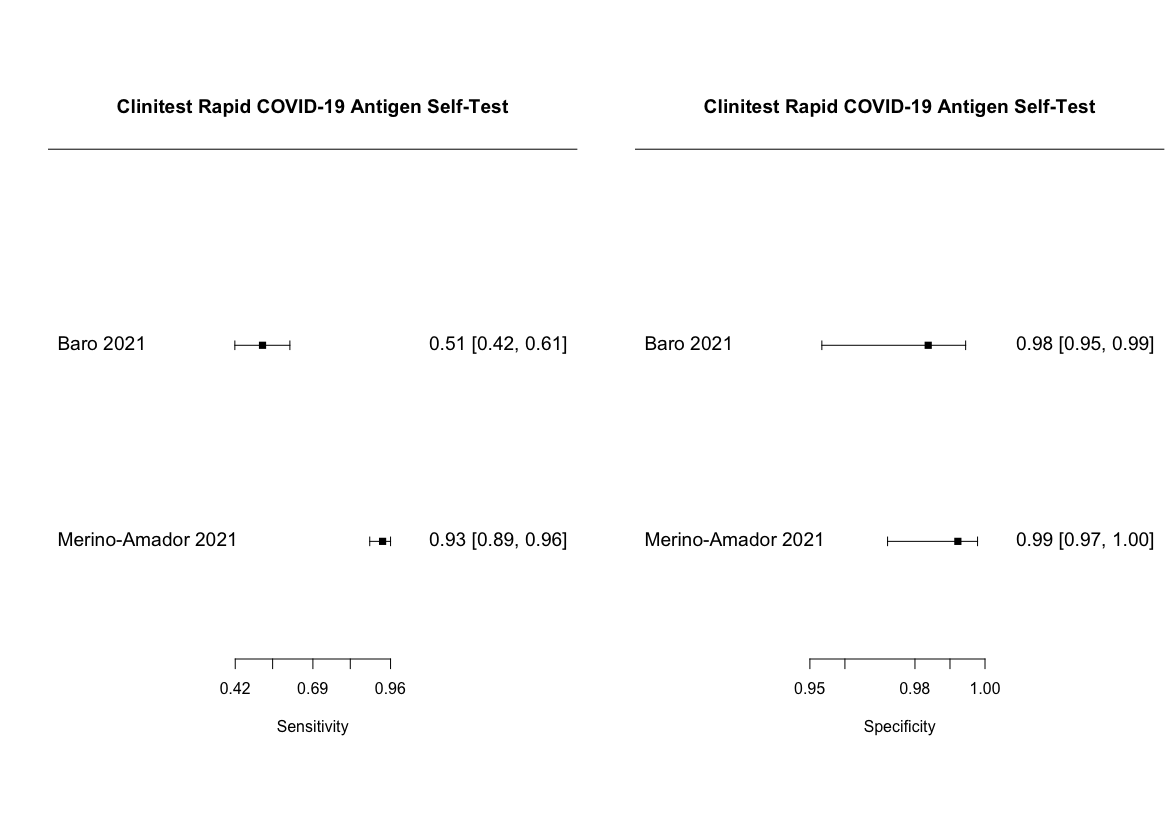


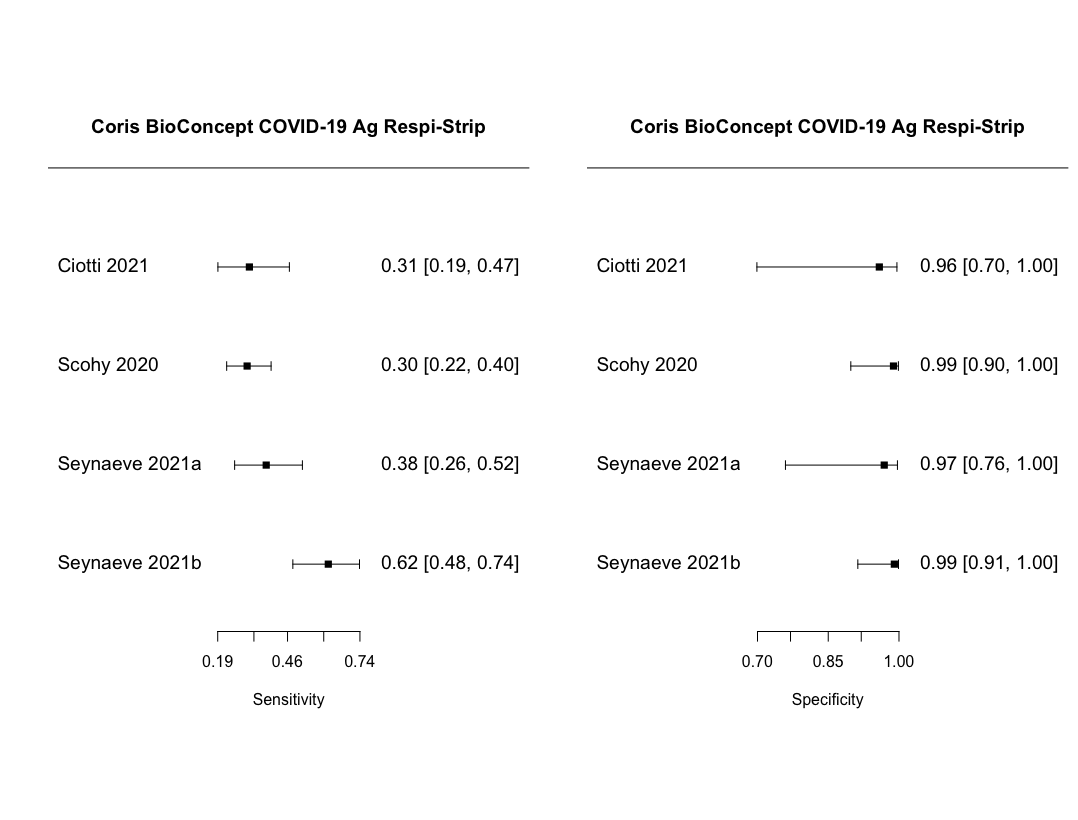

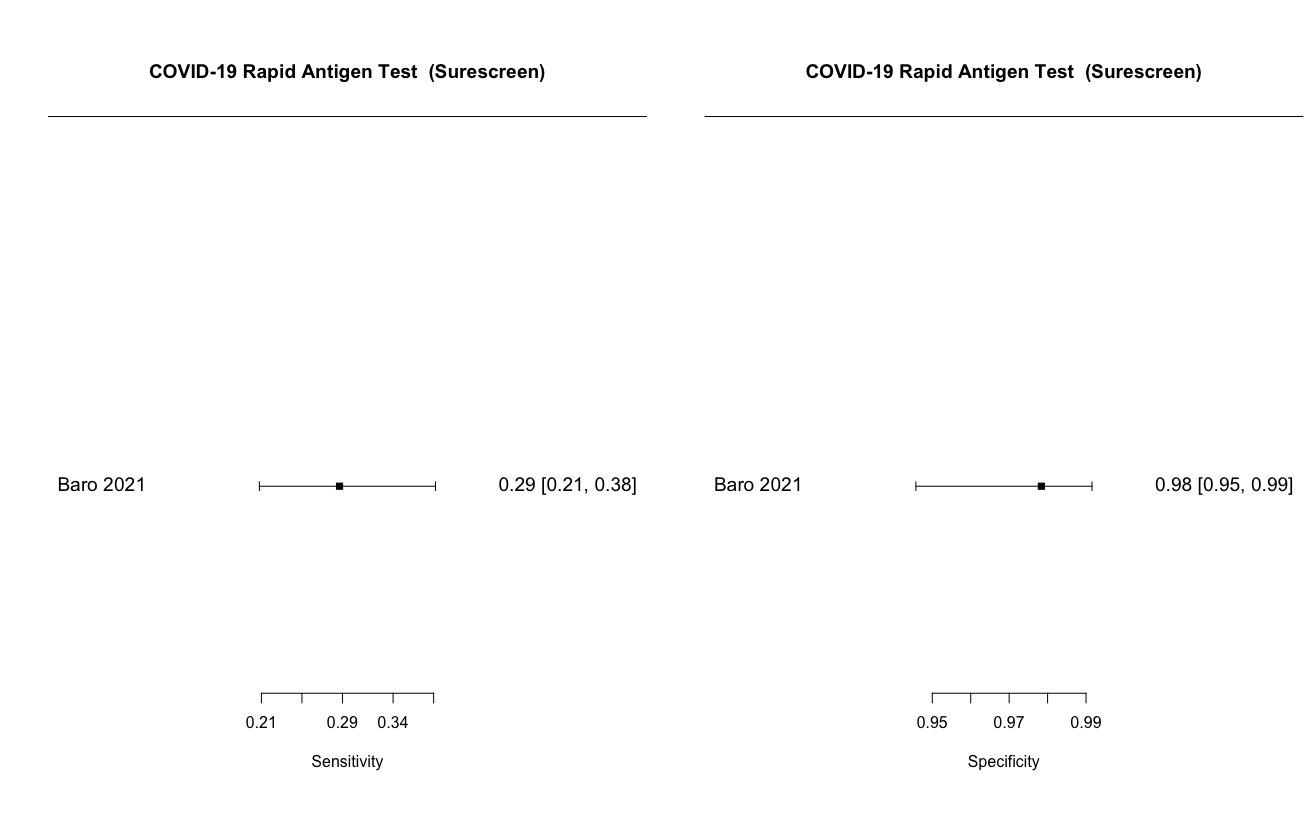


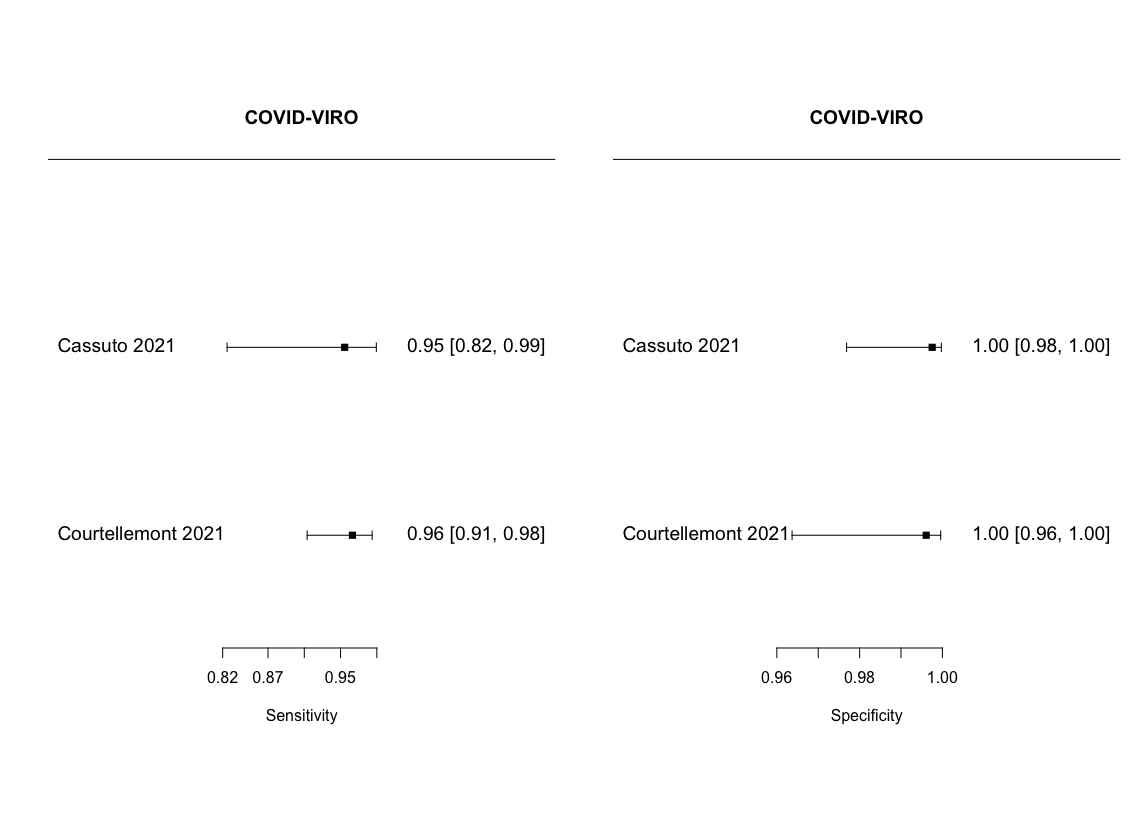


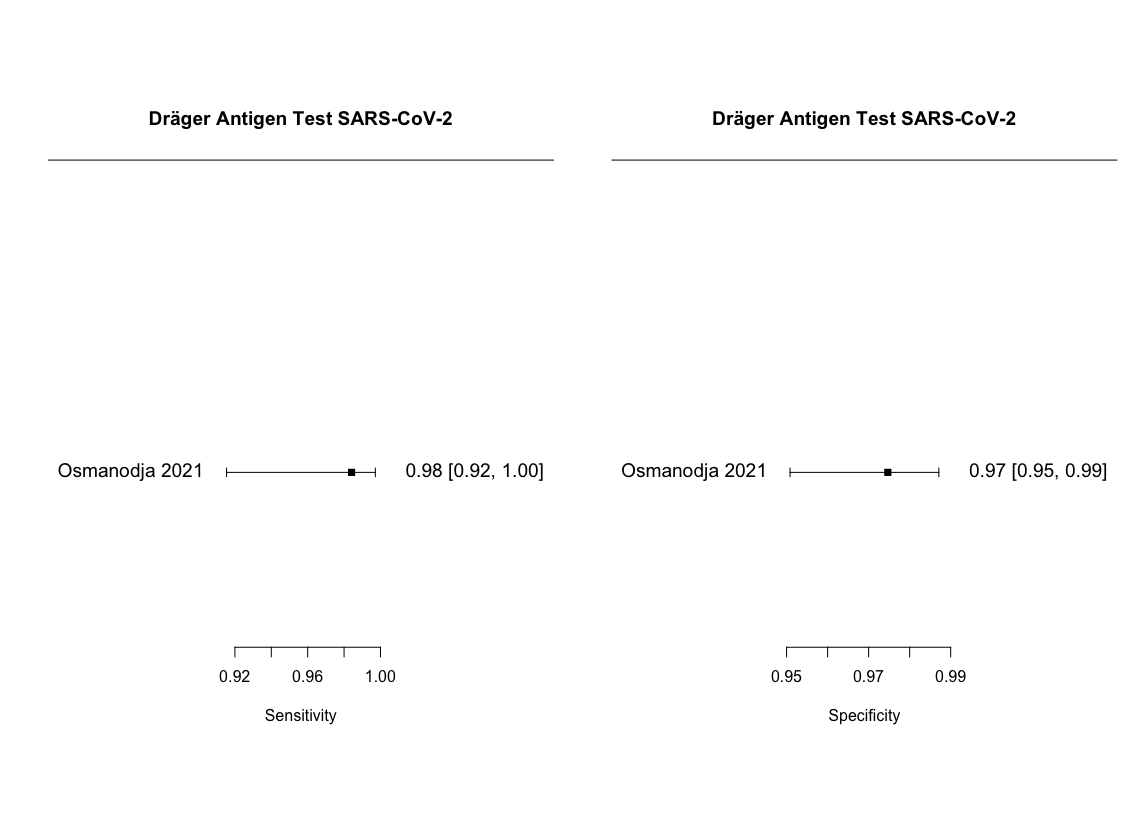


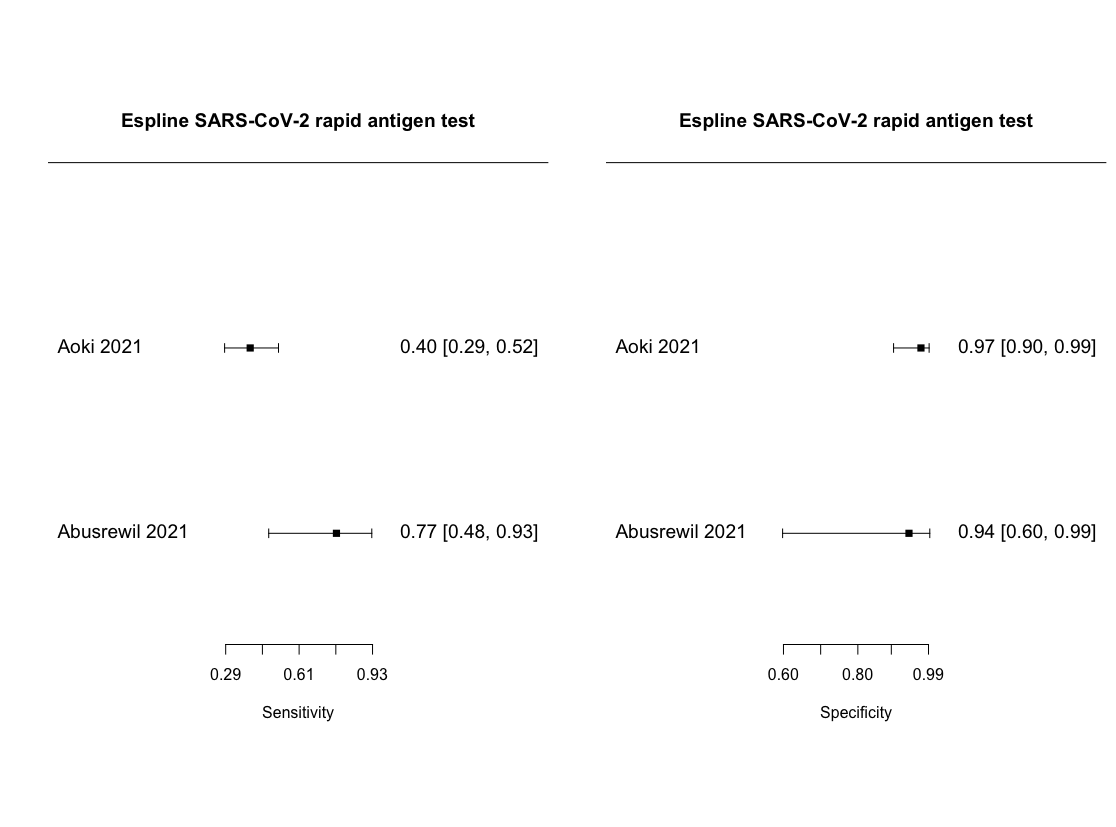


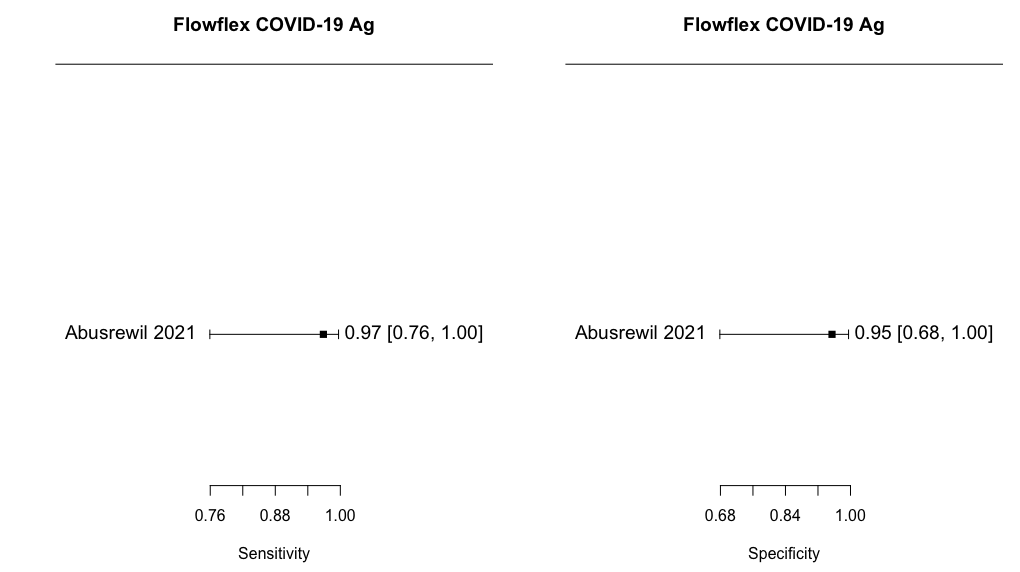


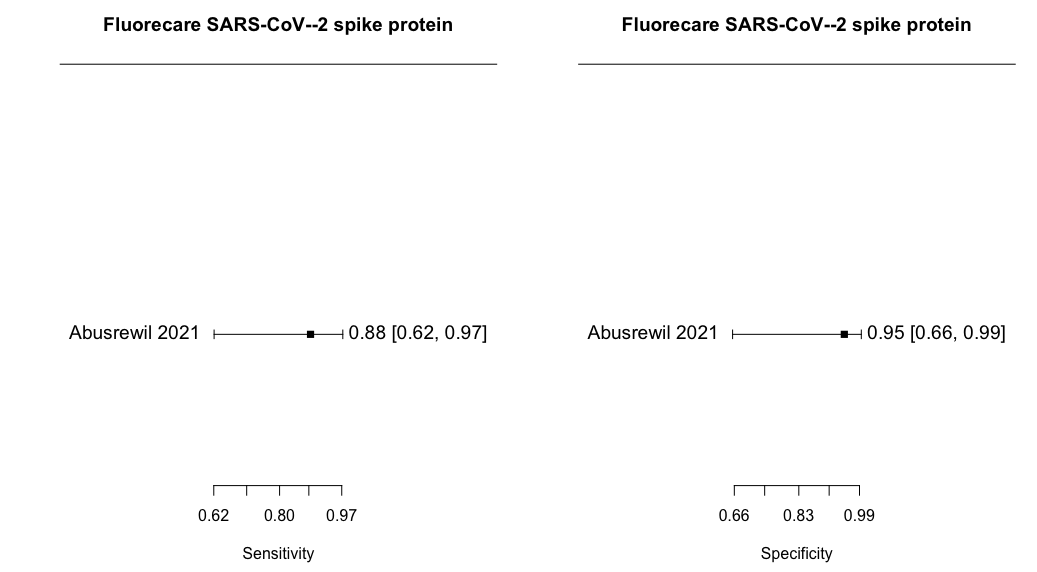


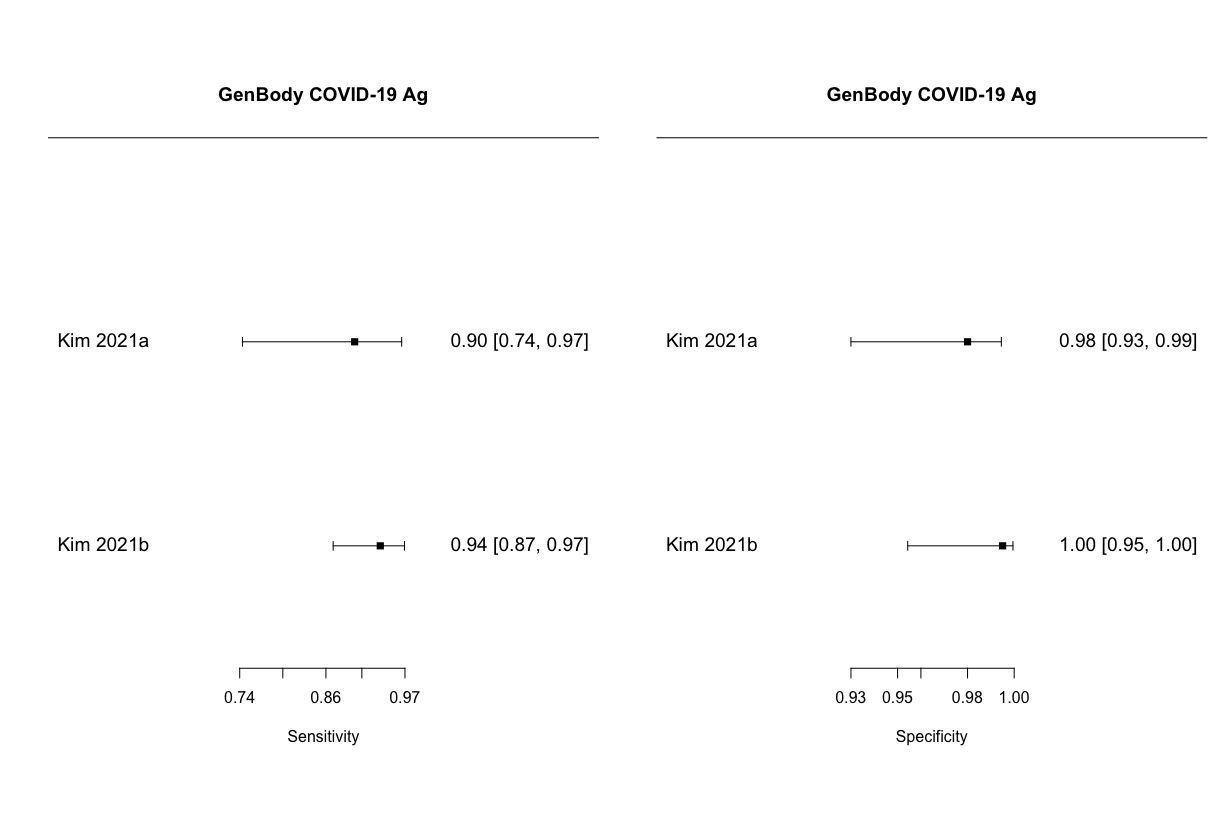


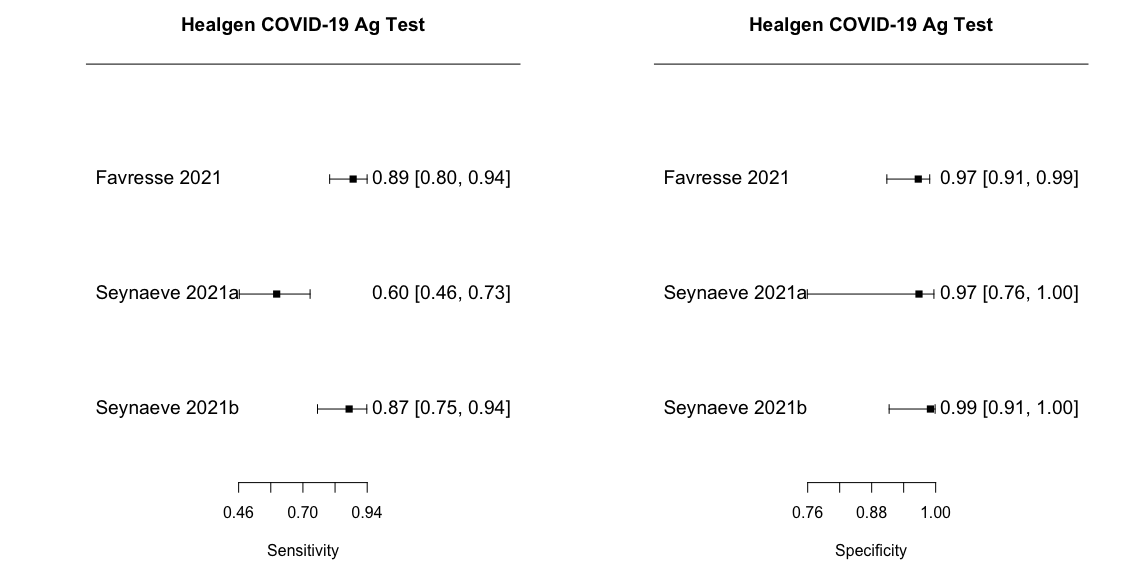


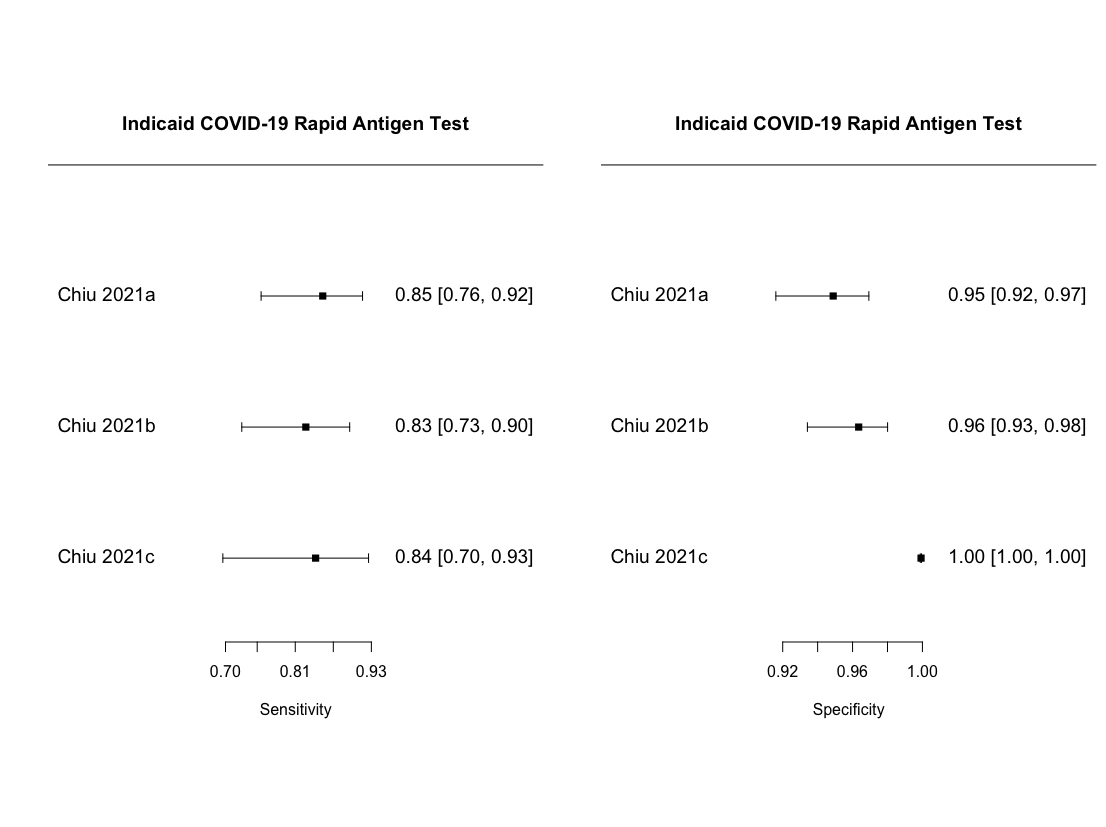


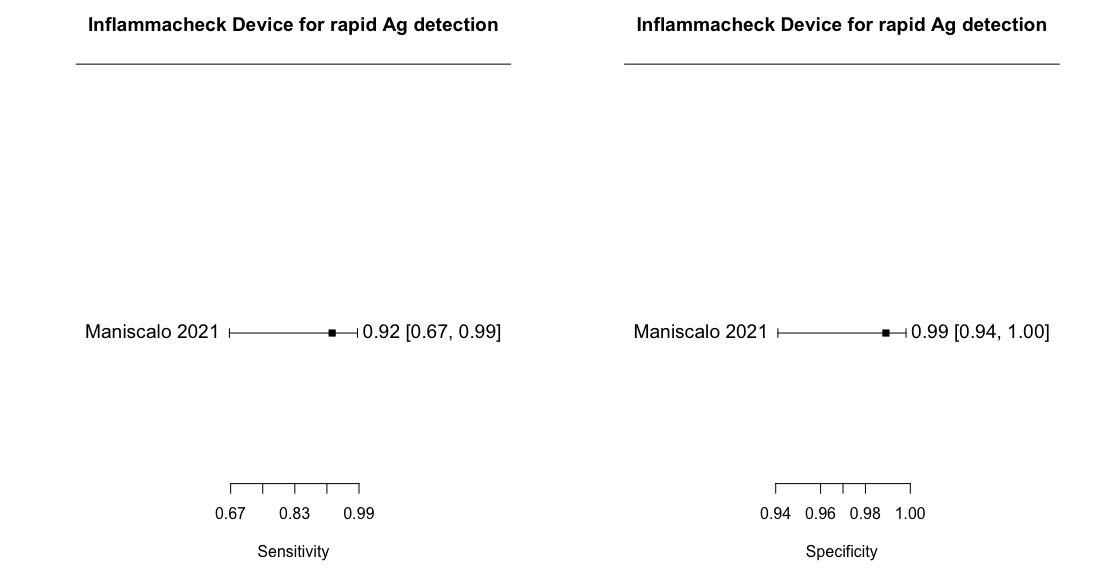


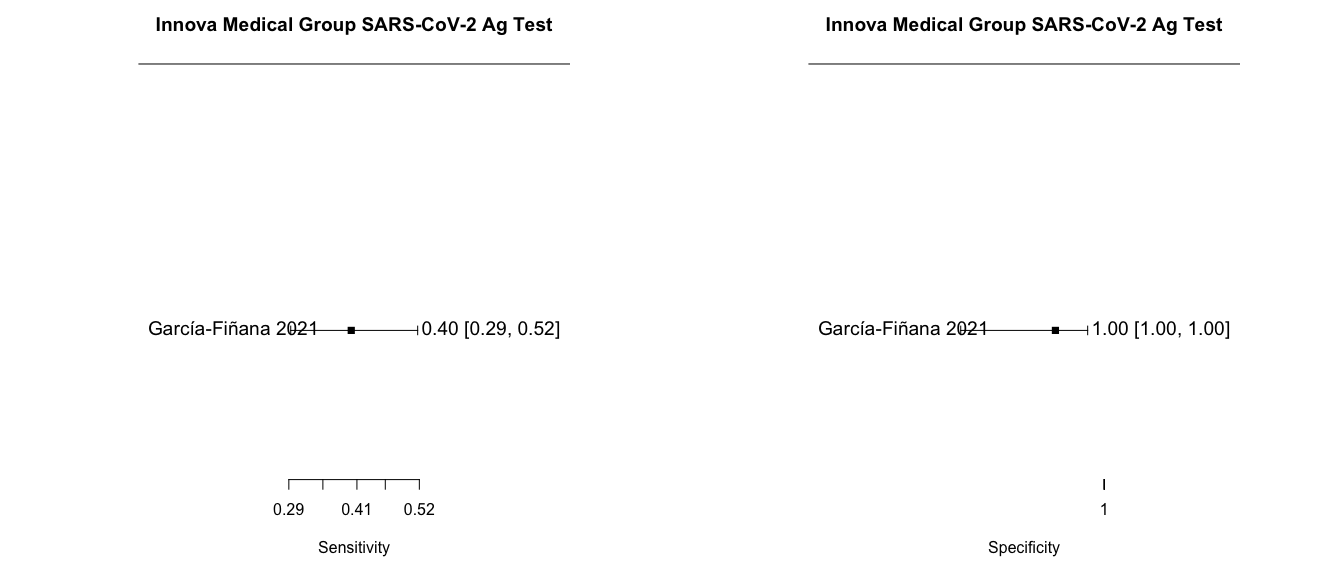


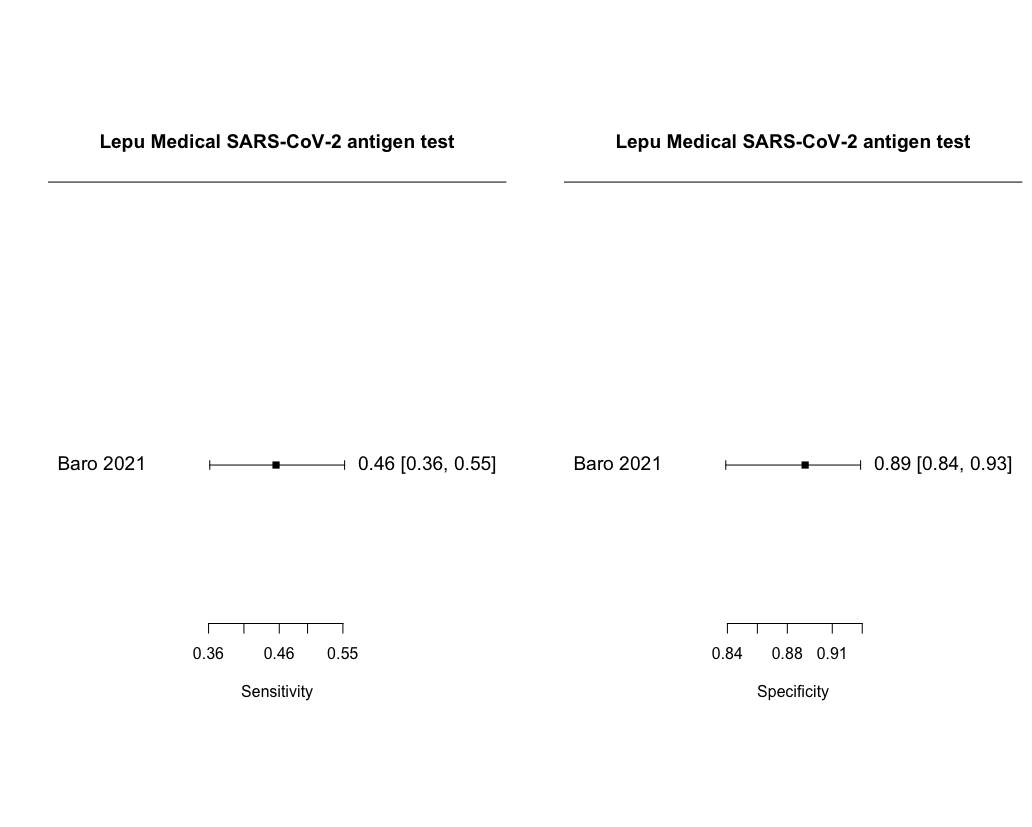


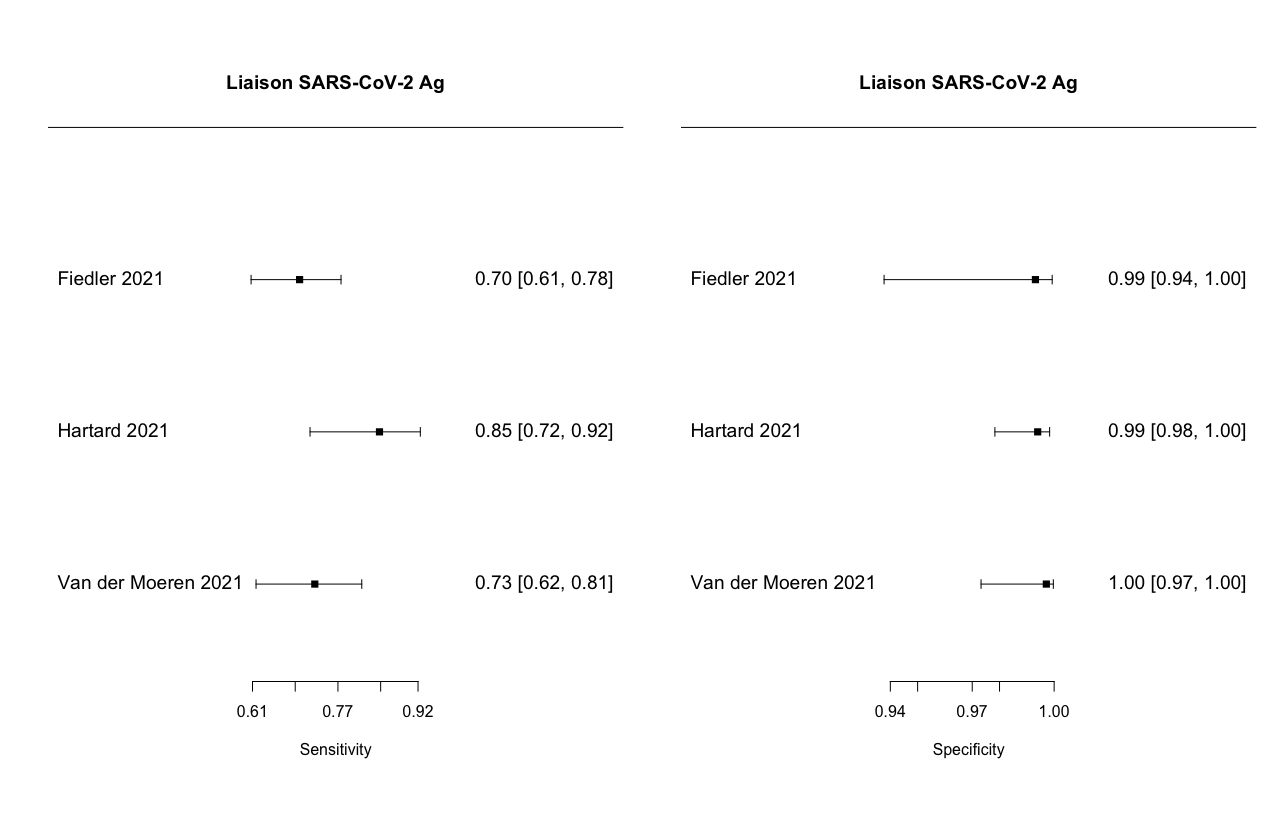


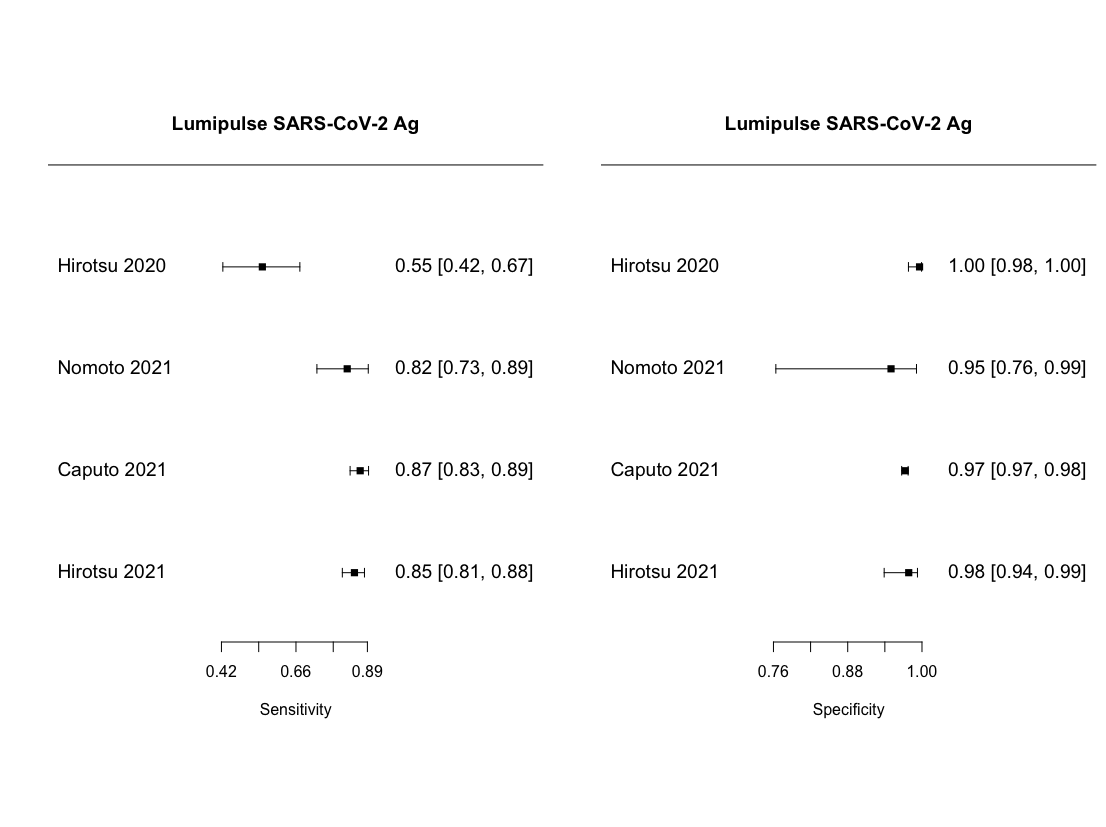


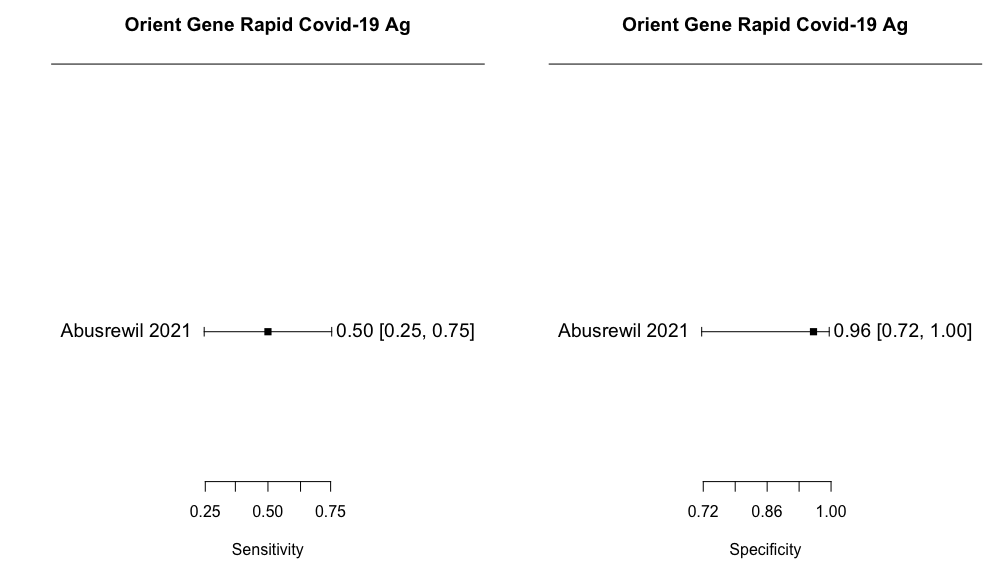


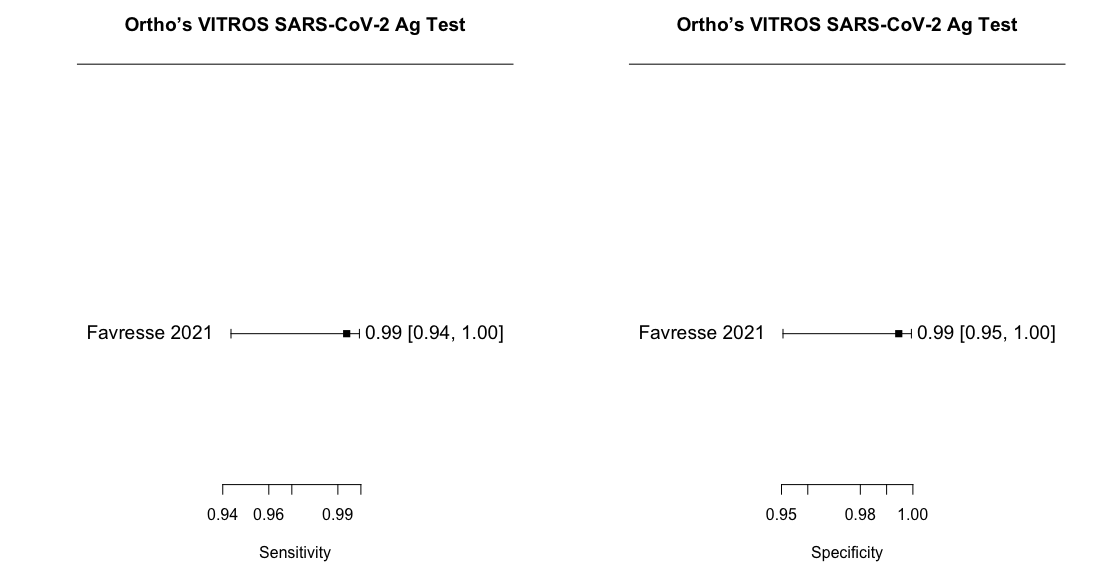


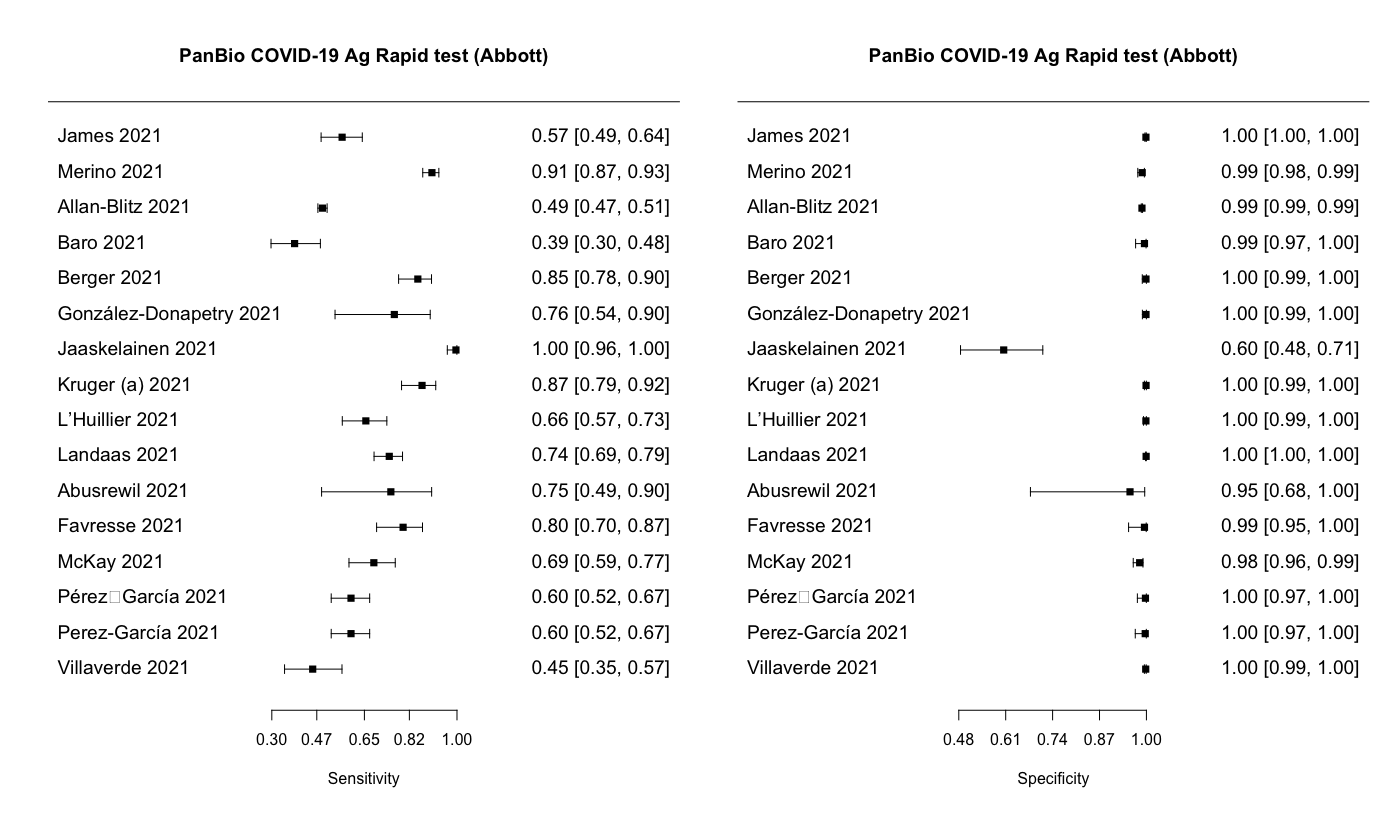


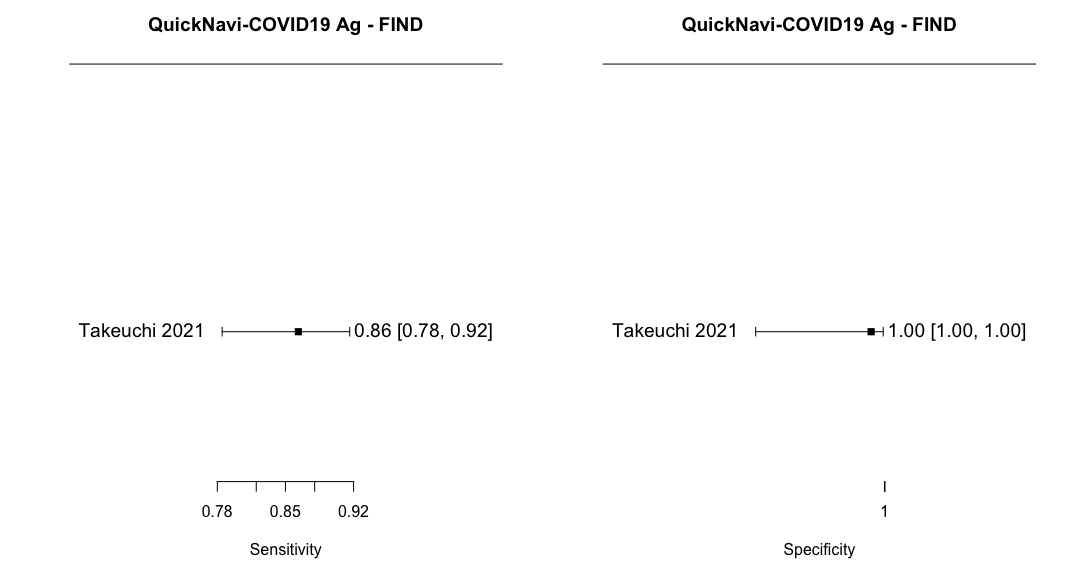


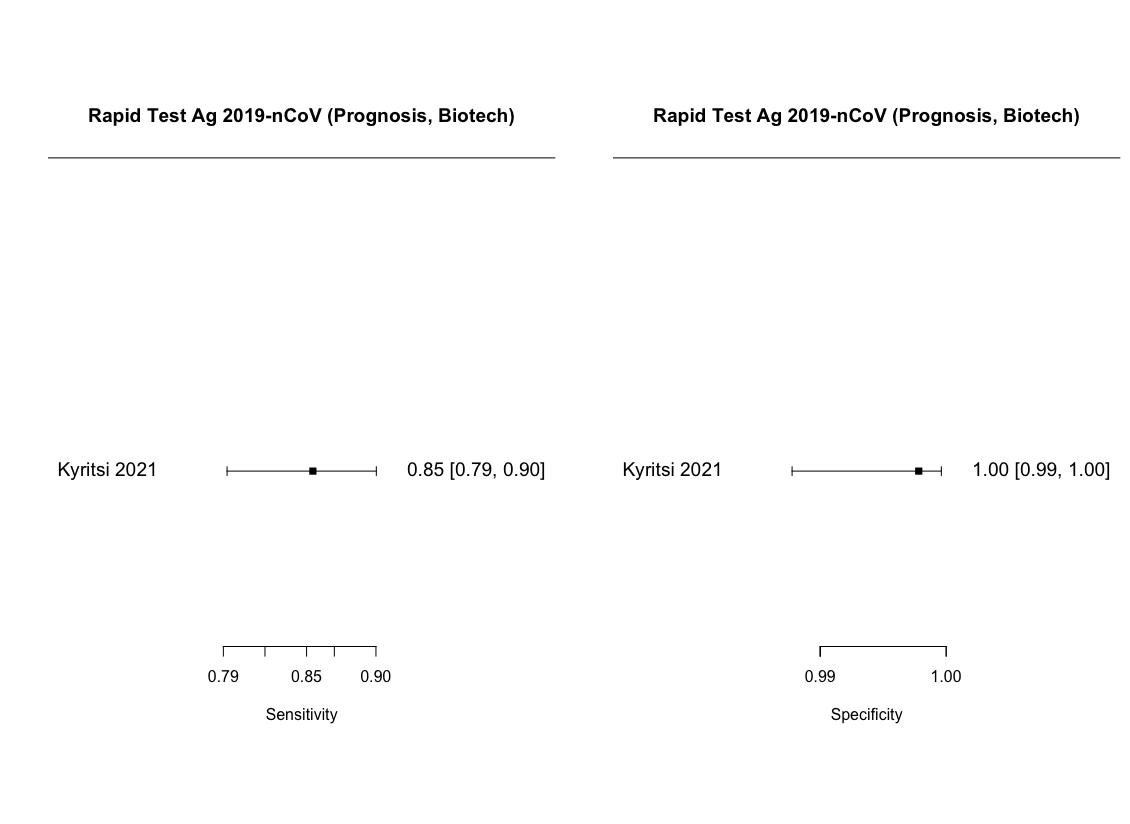


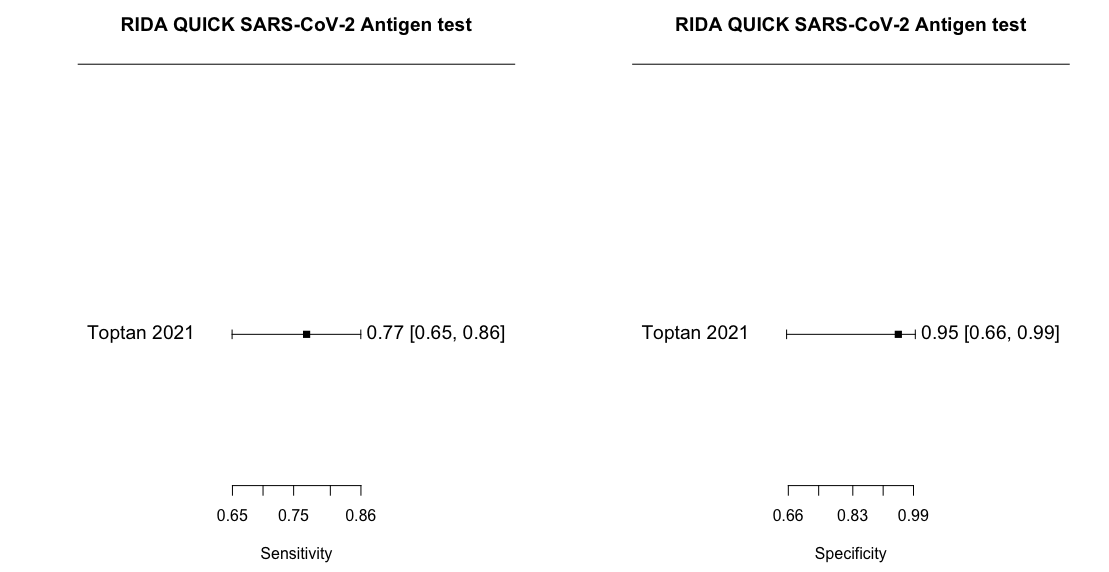


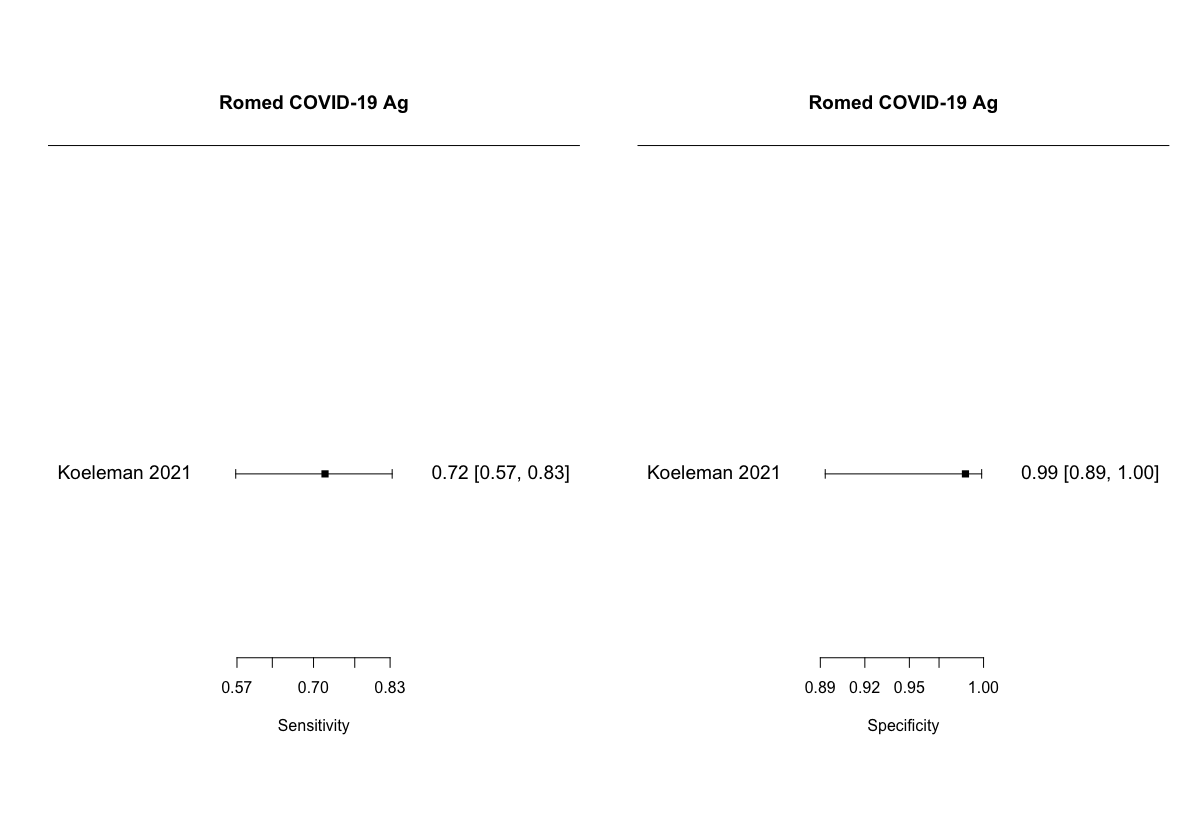


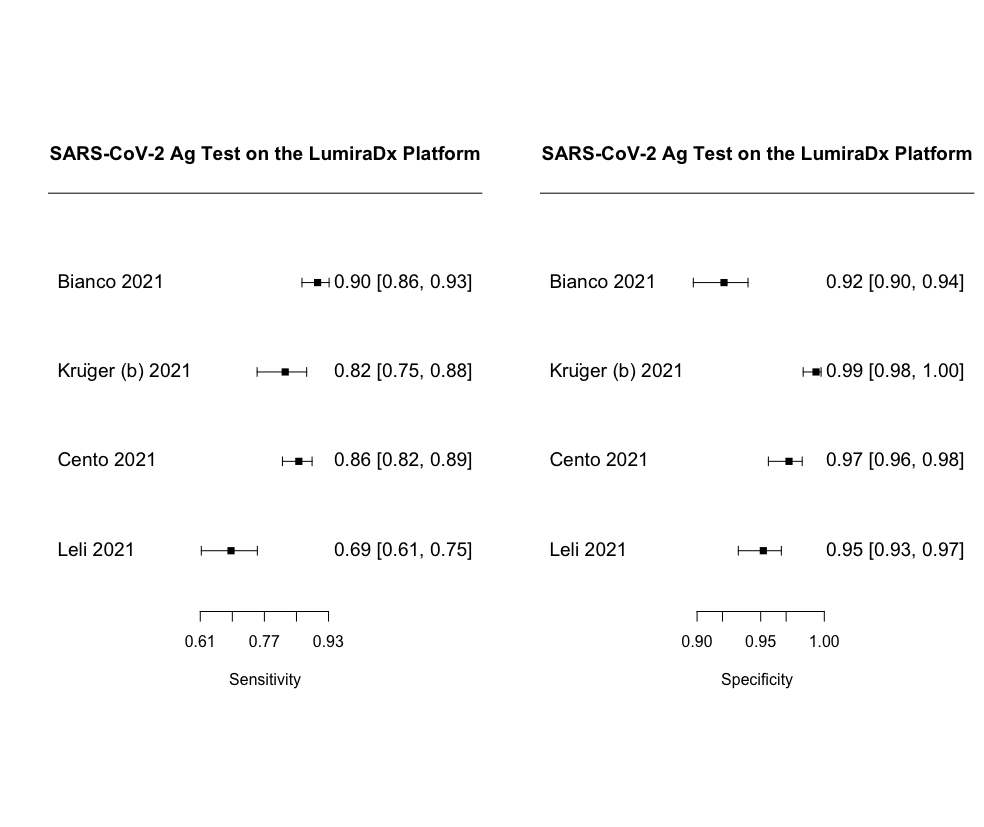


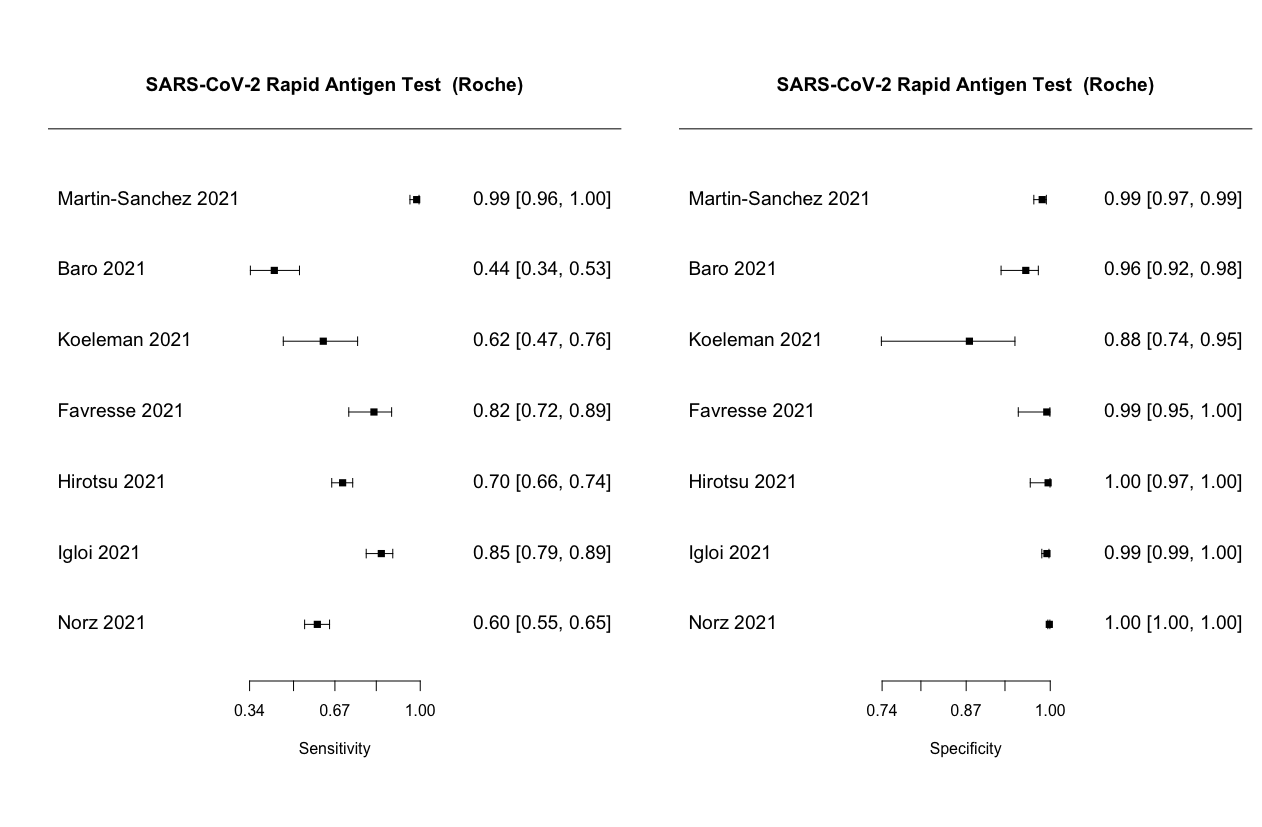


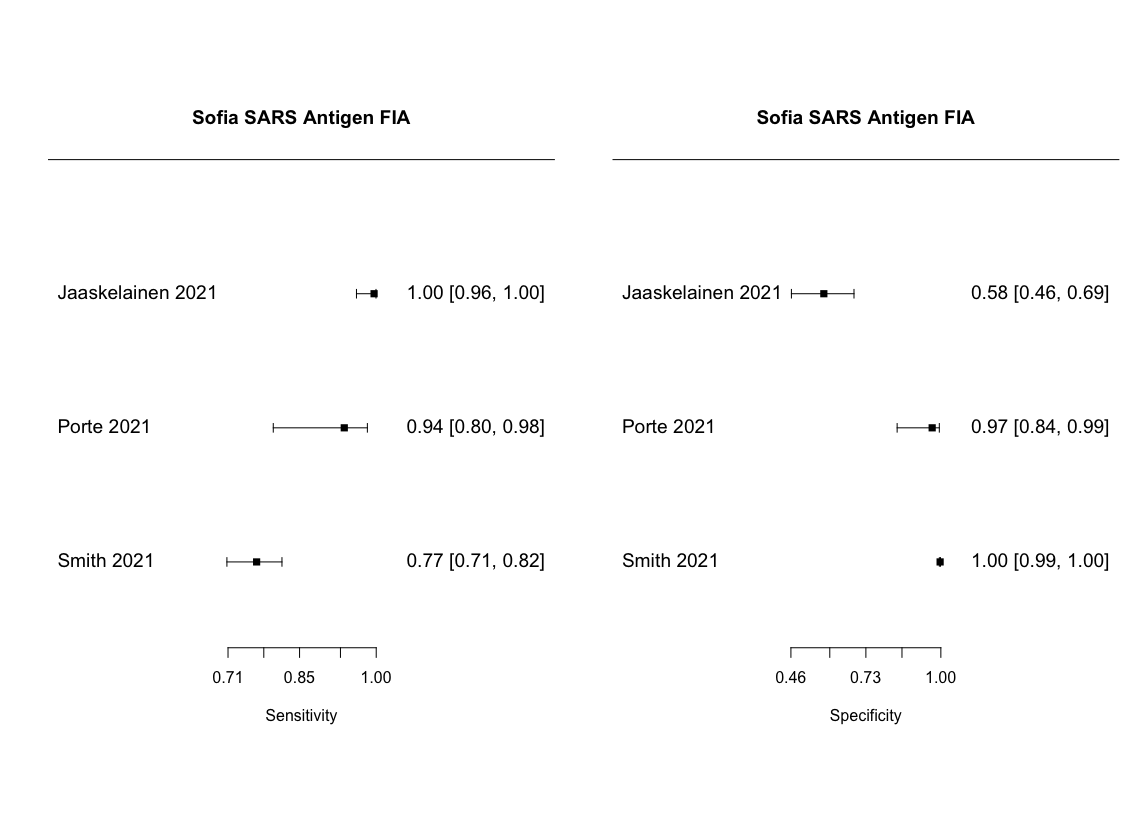


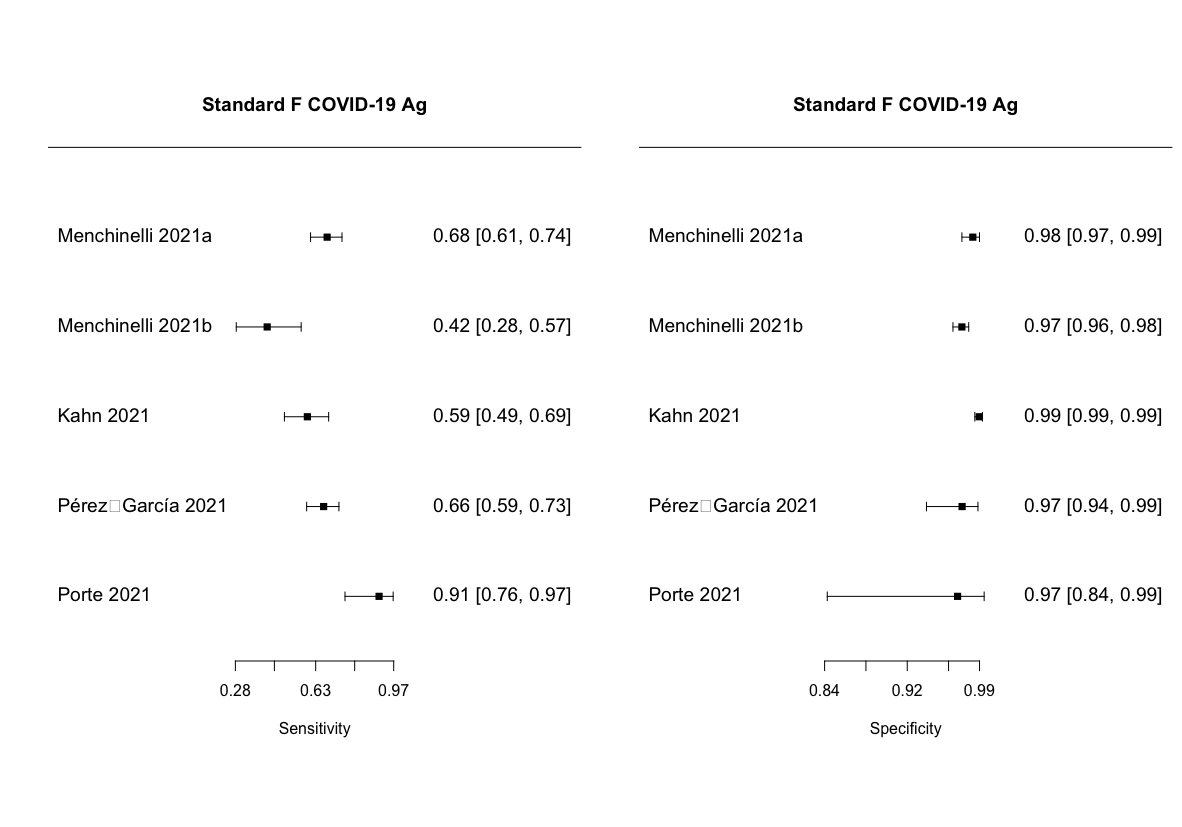


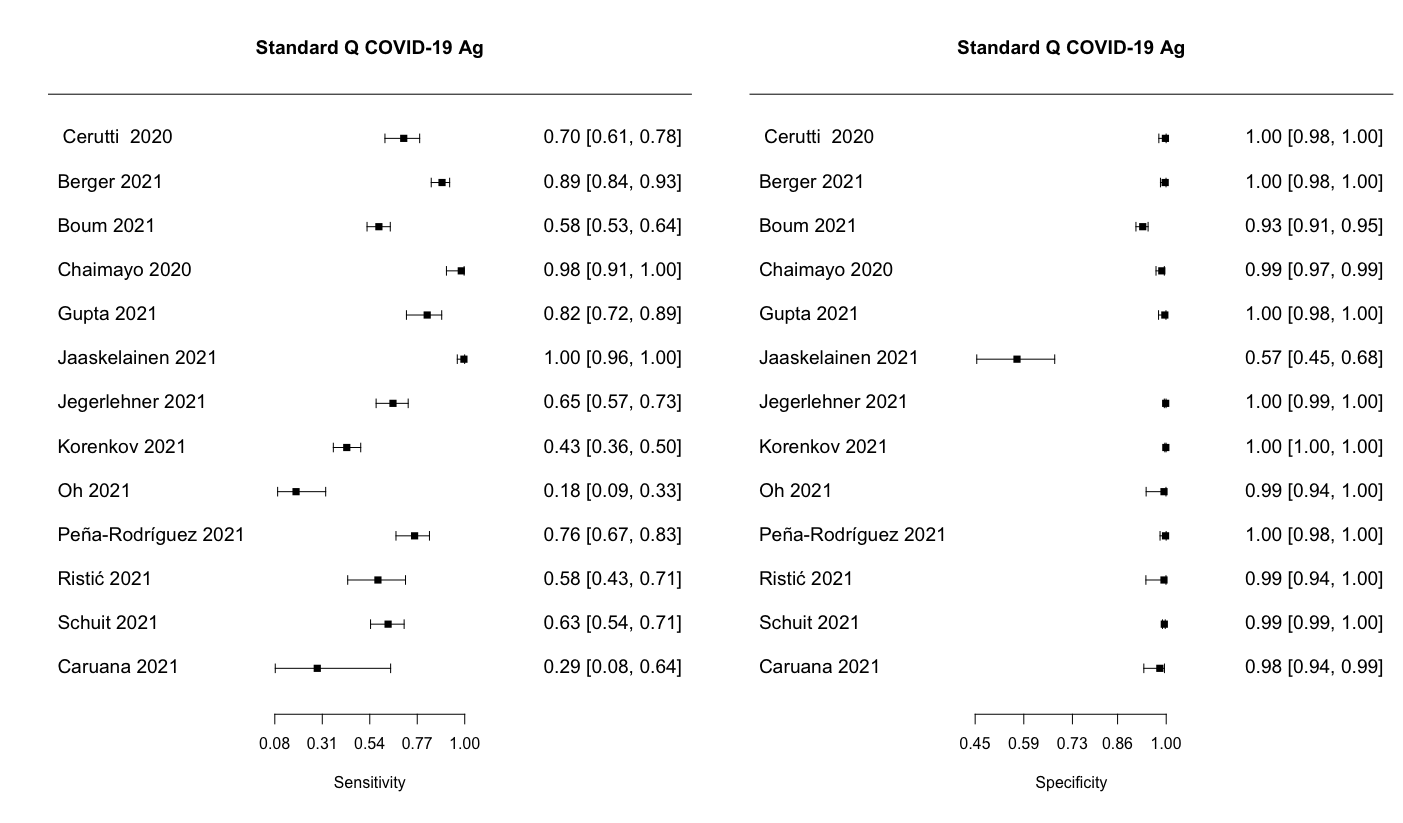


# Appendix 6. Forest plots of studies assessing each rapid molecular test separately


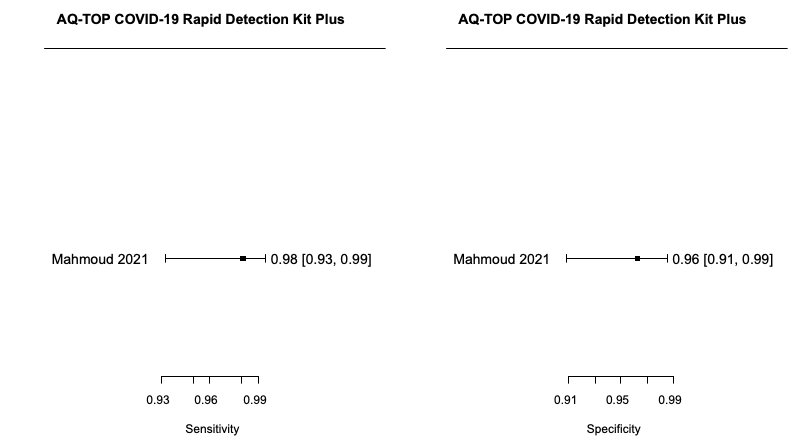


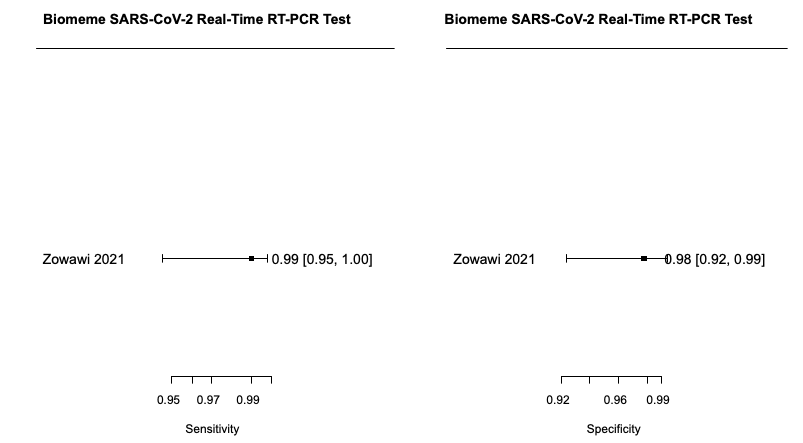


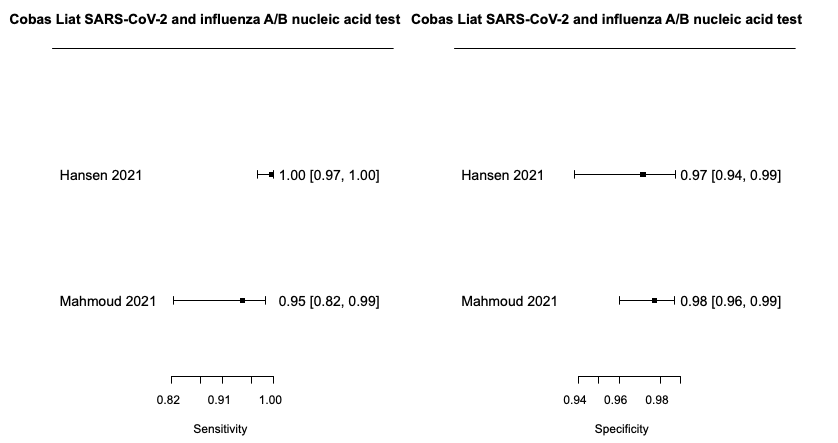


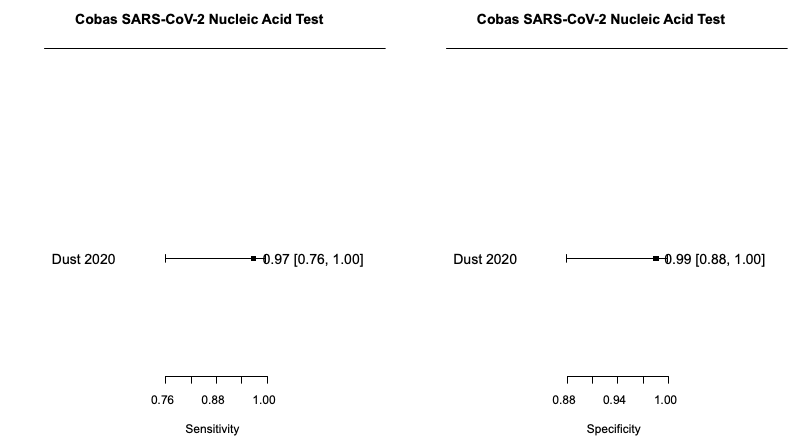


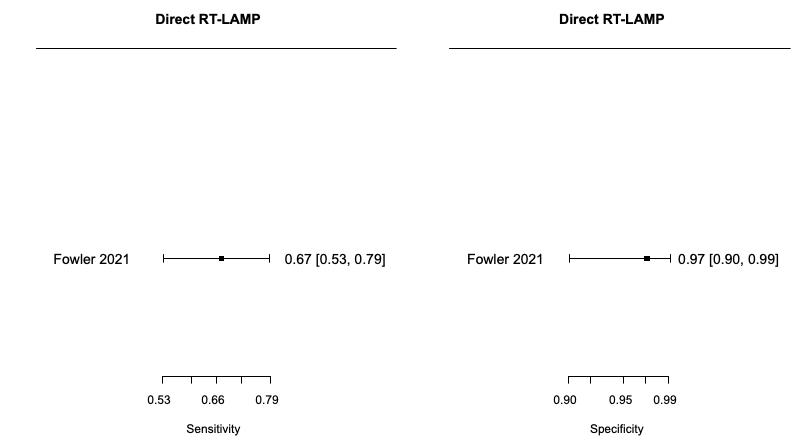


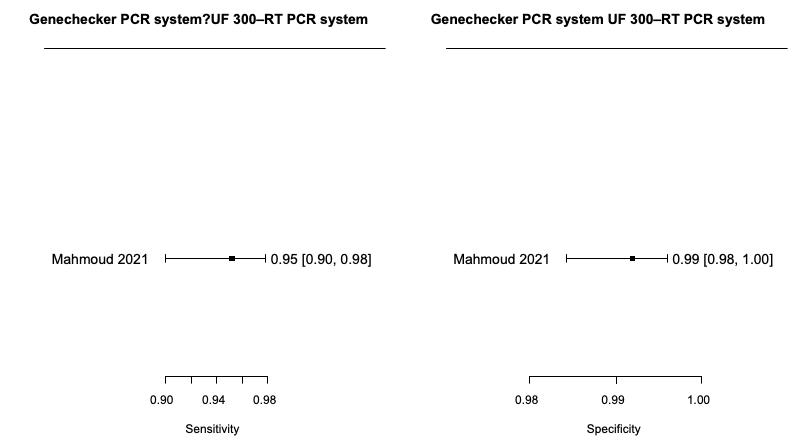


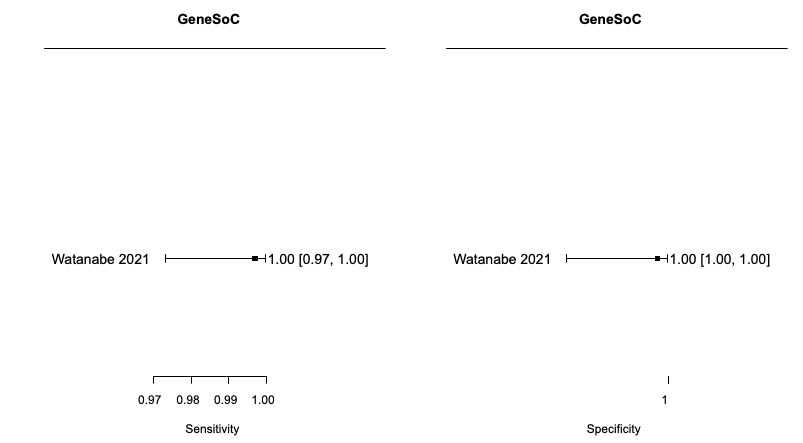


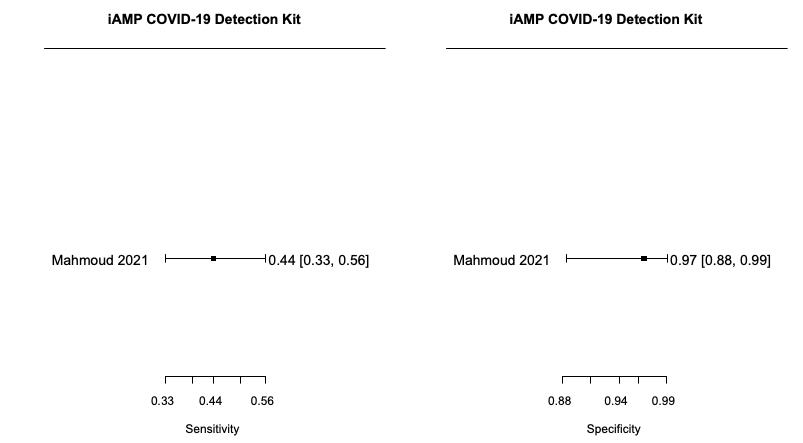


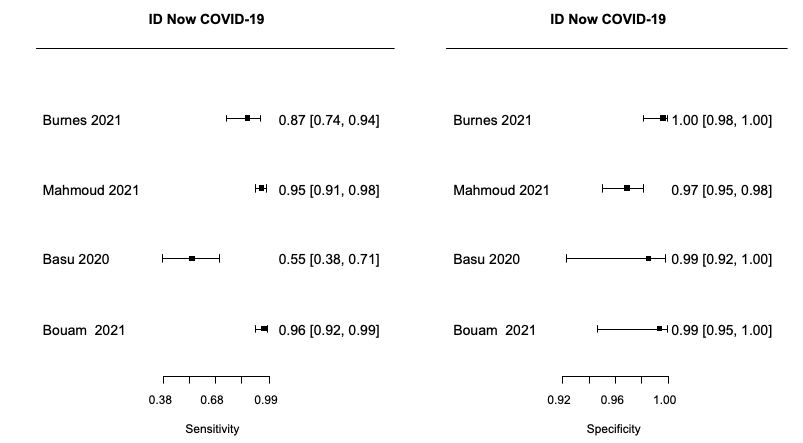


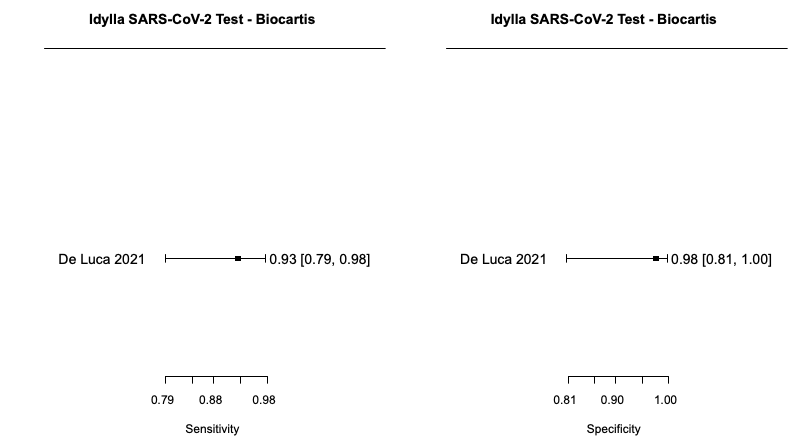


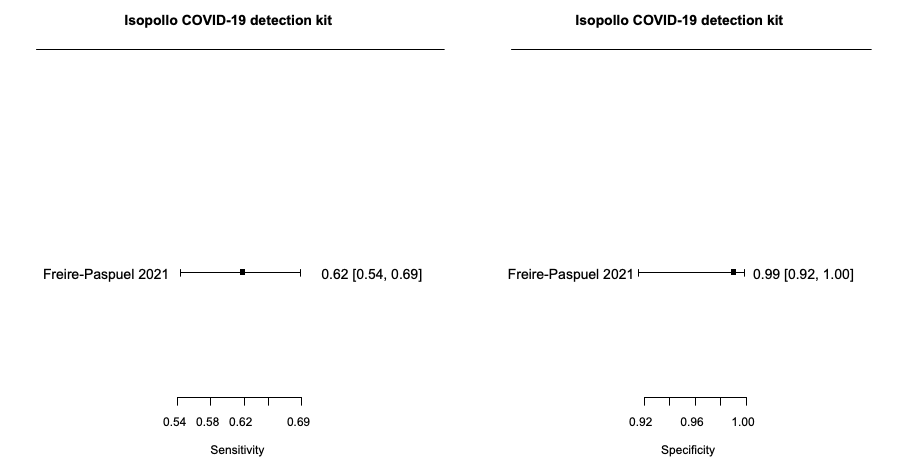


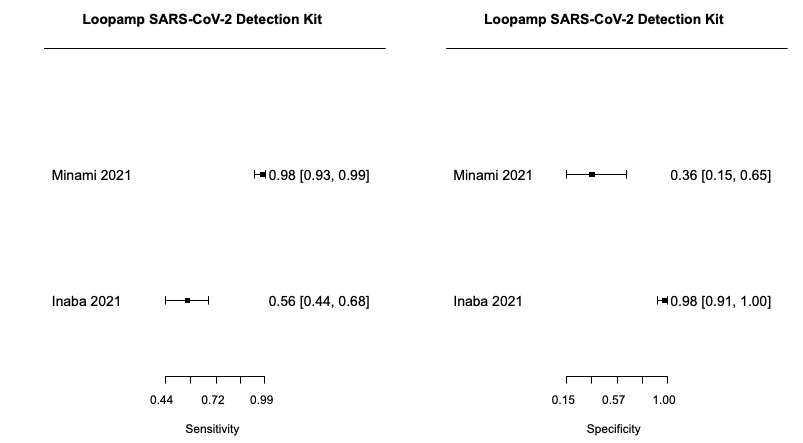


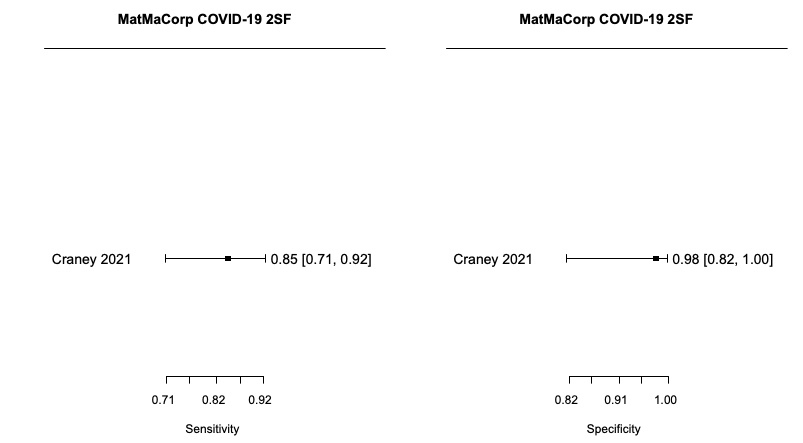


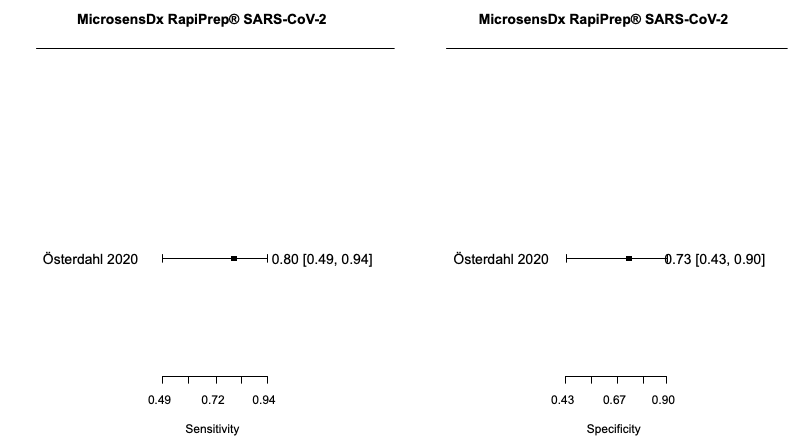


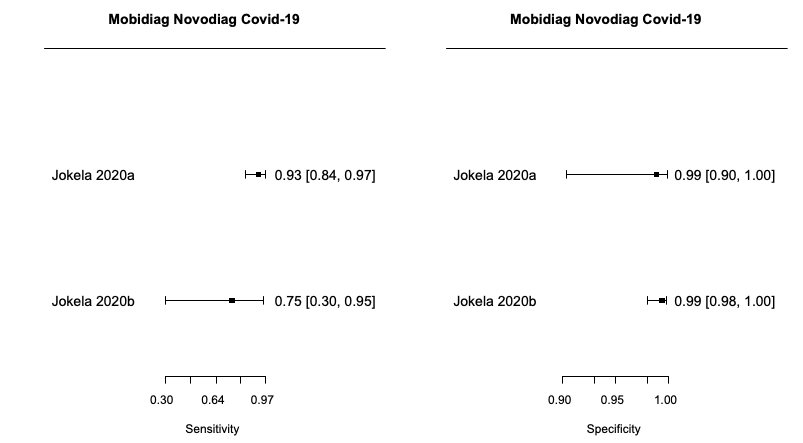


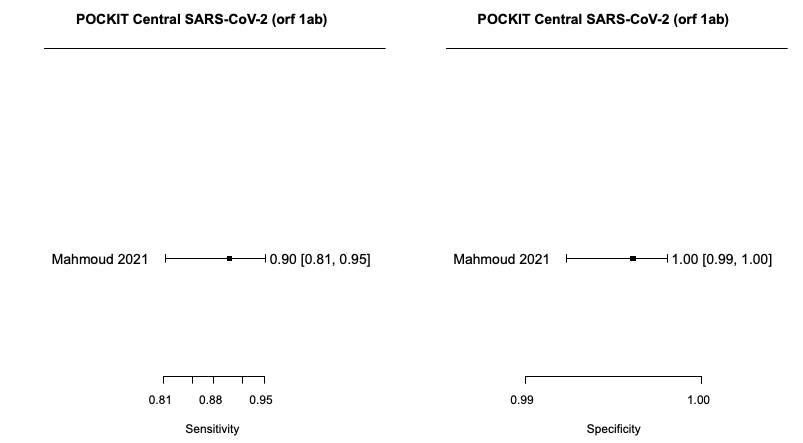


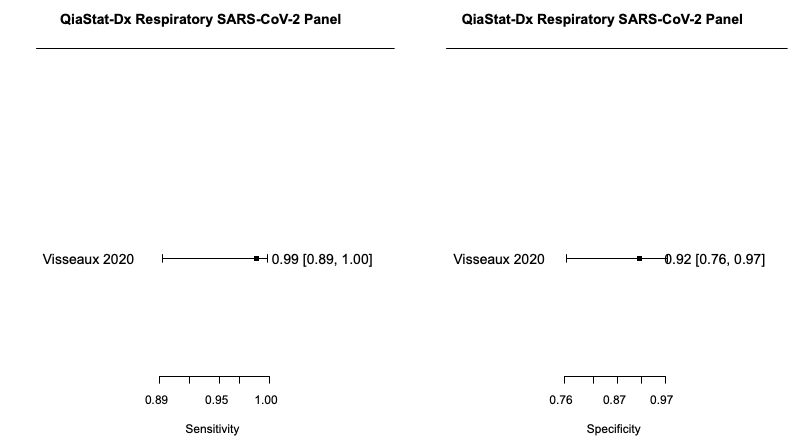


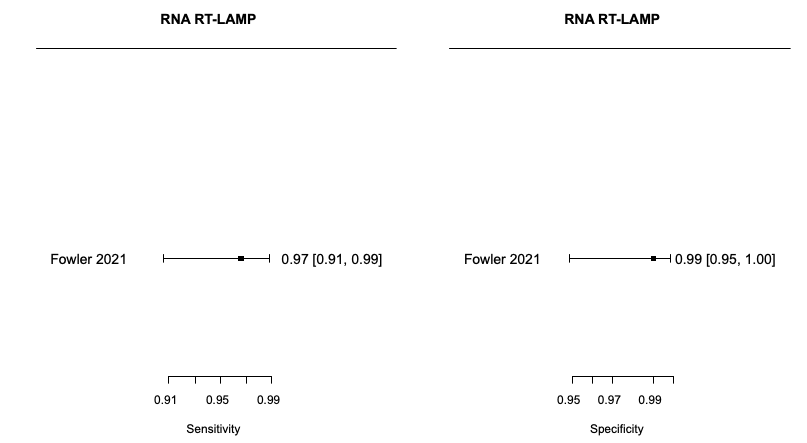


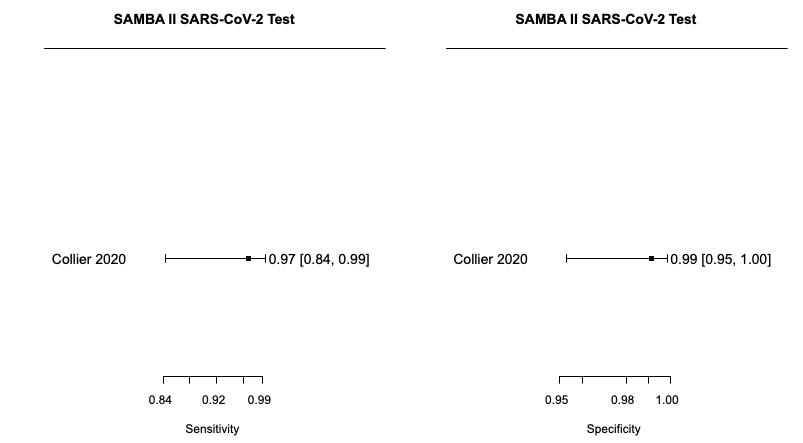


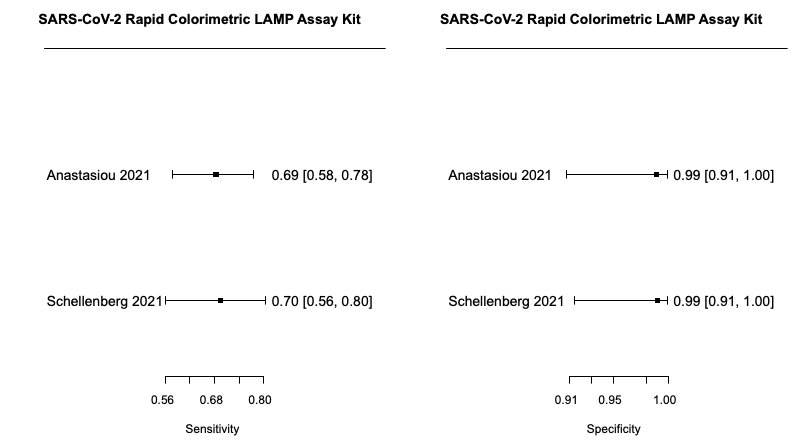


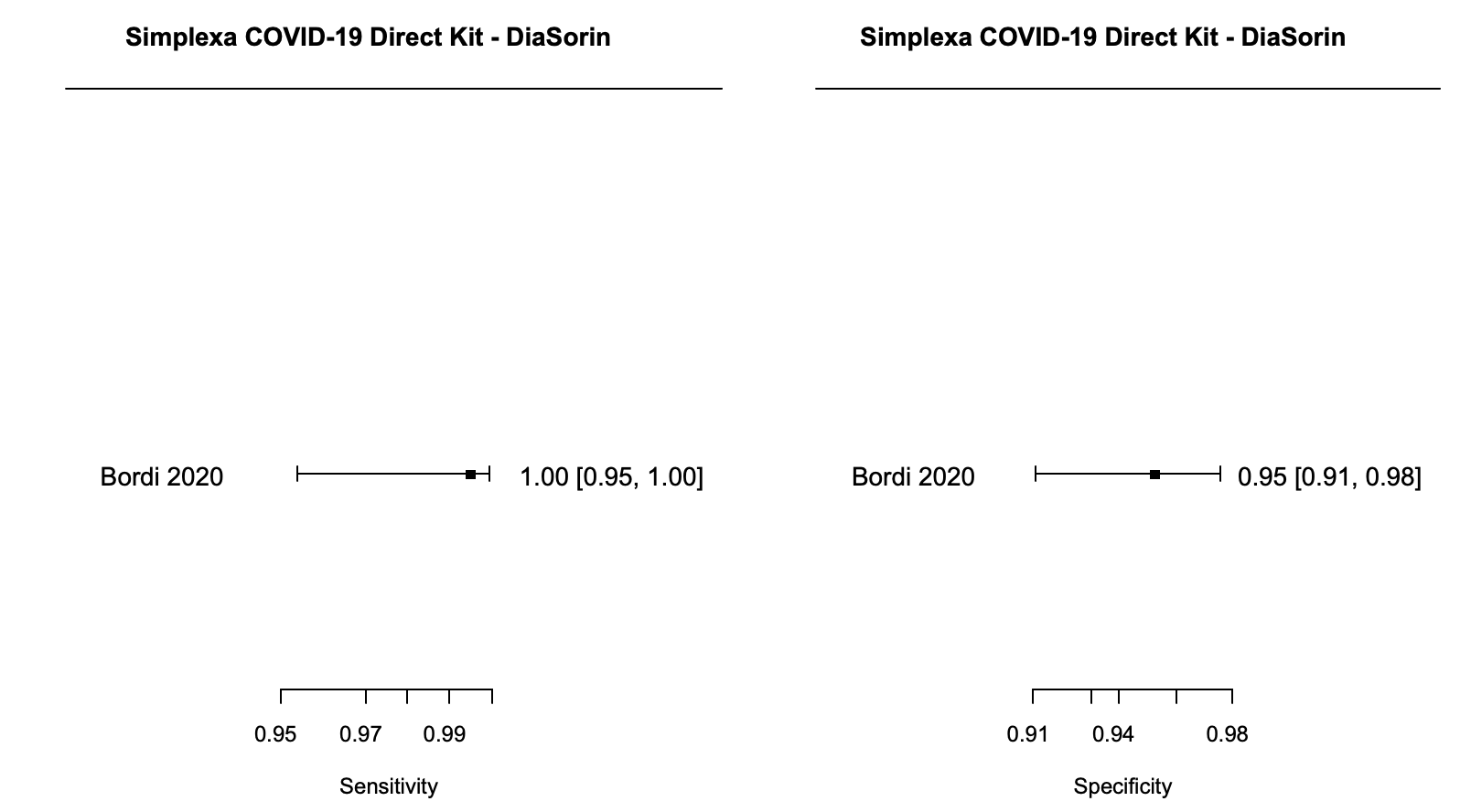


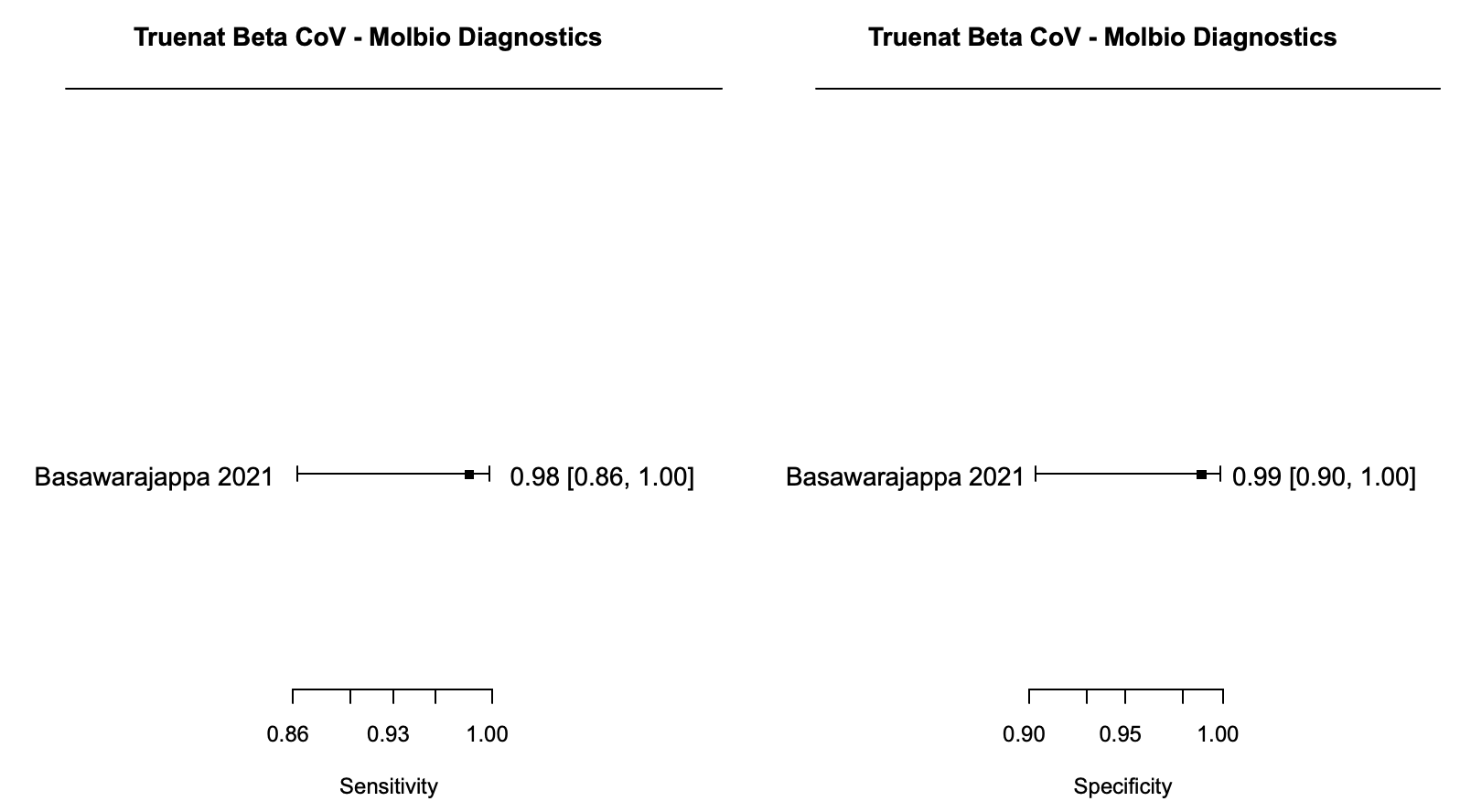


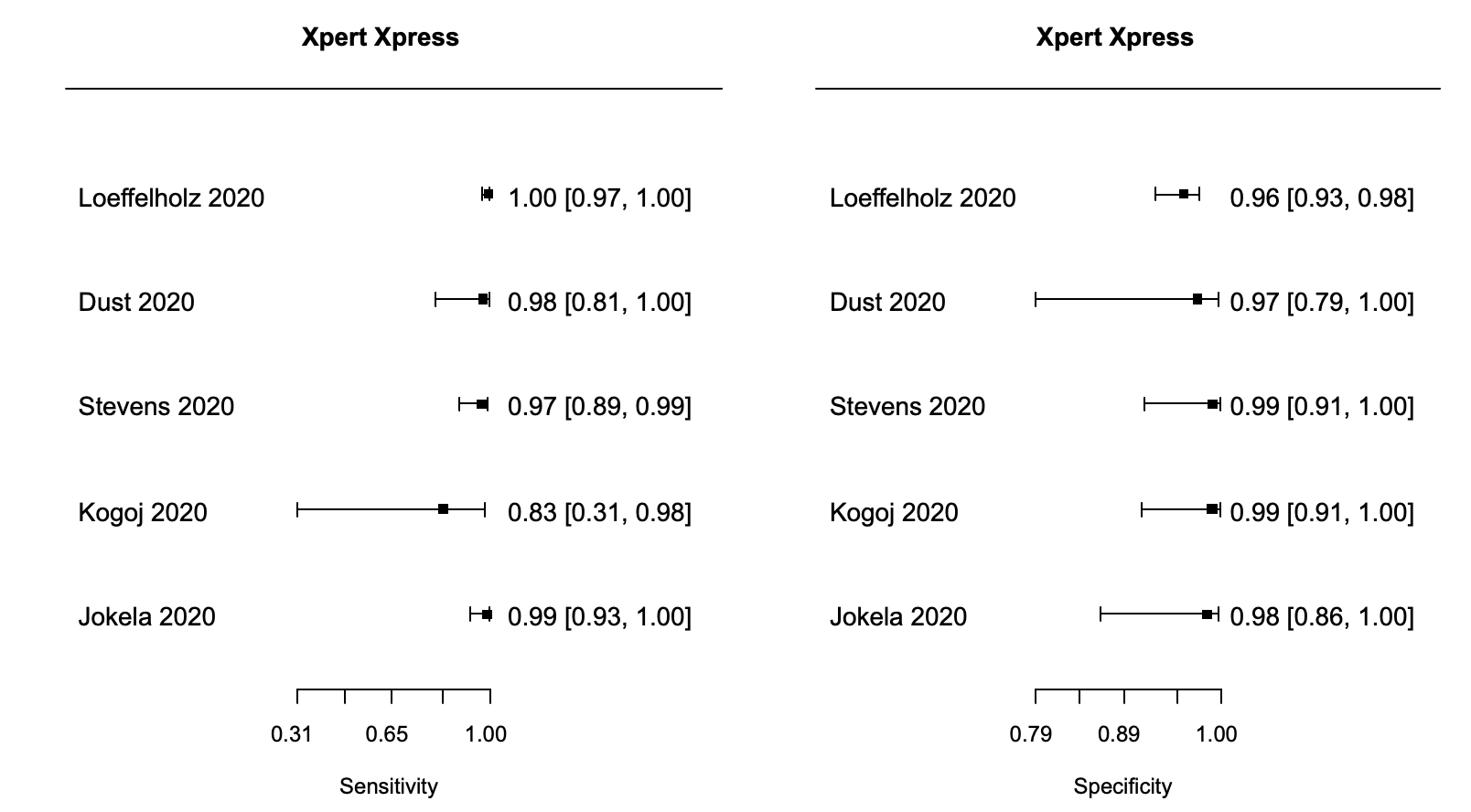


# Appendix 7. ROC space and summary estimate for a) any rapid antigen test, and b) any rapid molecular test included in the DTA bivariate meta-analysis model

| *(a) Any rapid antigen test* | *(b) Any rapid molecular test* |
| --- | --- |
| 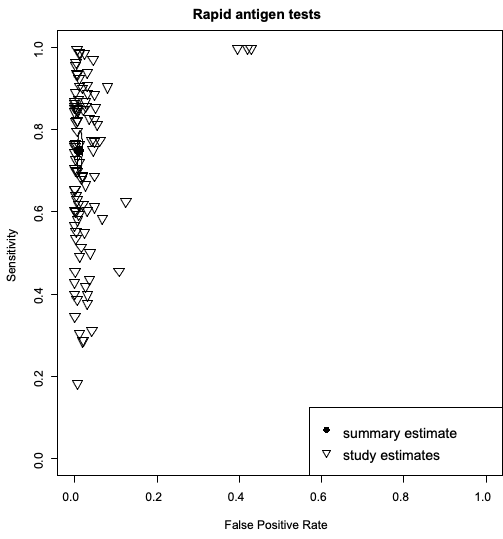 | 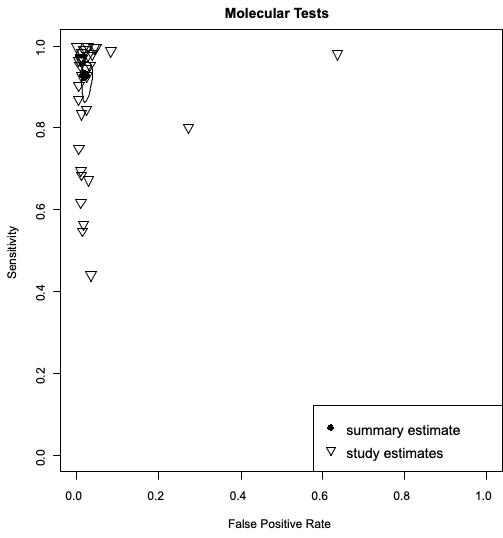 |

# Appendix 8: Transitivity assessment by index test comparison

| **Studies** | **Test Comparison*** | **% of symptomatic / % of asymptomatic** | **% of test sample type** | **Mean Age** | **Participant type** |
| --- | --- | --- | --- | --- | --- |
| ***Rapid Antigen Tests*** | | | | | |
| Seynaeve 2021a; Seynaeve 2021b^1^ | Coris BioConcept COVID-19 Ag Respi-Strip vs Healgen COVID-19 Ag Test | NA/NA | 100% Nasopharyngeal | NA | 100% General Public |
| Baro 2021^2^ | COVID-19 Rapid Antigen Test (Surescreen) vs Lepu Medical SARS-CoV-2 antigen test | 100% / 0% | 100% Nasopharyngeal | NA | 100% General Public |
| Baro 2021^2^ | COVID-19 Rapid Antigen Test (Surescreen) vs PanBio COVID-19 Ag Rapid test (Abbott) | 100% / 0% | 100% Nasopharyngeal | NA | NA |
| Baro 2021^2^ | COVID-19 Rapid Antigen Test (Surescreen) vs SARS-CoV-2 Rapid Antigen Test (Roche) | 100% / 0% | 100% Nasopharyngeal | NA | NA |
| Abusrewil 2021^3^ | Espline SARS-CoV-2 rapid antigen test vs Flowflex COVID-19 Ag | NA/NA | 100% Nasopharyngeal | 40.8 | 100% General Public |
| Abusrewil 2021^3^ | Espline SARS-CoV-2 rapid antigen test vs Fluorecare SARS-CoV--2 spike protein | NA/NA | 100% Nasopharyngeal | 40.8 | 100% General Public |
| Abusrewil 2021^3^ | Espline SARS-CoV-2 rapid antigen test vs Orient Gene Rapid Covid-19 Ag | NA/NA | 100% Nasopharyngeal | 40.8 | 100% General Public |
| Abusrewil 2021^3^ | Espline SARS-CoV-2 rapid antigen test vs PanBio COVID-19 Ag Rapid test (Abbott) | NA/NA | 100% Nasopharyngeal | 40.8 | 100% General Public |
| Abusrewil 2021^3^ | Flowflex COVID-19 Ag vs Fluorecare SARS-CoV--2 spike protein | NA/NA | 100% Nasopharyngeal | 40.8 | 100% General Public |
| Abusrewil 2021^3^ | Flowflex COVID-19 Ag vs Orient Gene Rapid Covid-19 Ag | NA/NA | 100% Nasopharyngeal | 40.8 | 100% General Public |
| Abusrewil 2021^3^ | Flowflex COVID-19 Ag vs PanBio COVID-19 Ag Rapid test (Abbott) | NA/NA | 100% Nasopharyngeal | 40.8 | 100% General Public |
| Abusrewil 2021^3^ | Fluorecare SARS-CoV--2 spike protein vs Orient Gene Rapid Covid-19 Ag | NA/NA | 100% Nasopharyngeal | 40.8 | 100% General Public |
| Abusrewil 2021^3^ | Fluorecare SARS-CoV--2 spike protein vs PanBio COVID-19 Ag Rapid test (Abbott) | NA/NA | 100% Nasopharyngeal | 40.8 | 100% General Public |
| Favresse 2021^4^ | Healgen COVID-19 Ag Test vs Ortho’s VITROS SARS-CoV-2 Ag Test | NA/NA | 100% Nasopharyngeal | 55.5 | 100% General Public |
| Favresse 2021^4^ | Healgen COVID-19 Ag Test vs PanBio COVID-19 Ag Rapid test (Abbott) | NA/NA | 100% Nasopharyngeal | 55.5 | 100% General Public |
| Favresse 2021^4^ | Healgen COVID-19 Ag Test vs SARS-CoV-2 Rapid Antigen Test (Roche) | NA/NA | 100% Nasopharyngeal | 55.5 | 100% General Public |
| Abusrewil 2021^3^ | AMP Rapid Test SARS-CoV-2 Ag  vs Espline SARS-CoV-2 rapid antigen test | NA/NA | 100% Nasopharyngeal | 40.8 | 100% General Public |
| Abusrewil 2021^3^ | AMP Rapid Test SARS-CoV-2 Ag  vs Flowflex COVID-19 Ag | NA/NA | 100% Nasopharyngeal | 40.8 | 100% General Public |
| Abusrewil 2021^3^ | AMP Rapid Test SARS-CoV-2 Ag  vs Fluorecare SARS-CoV--2 spike protein | NA/NA | 100% Nasopharyngeal | 40.8 | 100% General Public |
| Abusrewil 2021^3^ | AMP Rapid Test SARS-CoV-2 Ag  vs Orient Gene Rapid Covid-19 Ag | NA/NA | 100% Nasopharyngeal | 40.8 | 100% General Public |
| Abusrewil 2021^3^ | AMP Rapid Test SARS-CoV-2 Ag  vs PanBio COVID-19 Ag Rapid test (Abbott) | NA/NA | 100% Nasopharyngeal | 40.8 | 100% General Public |
| Abusrewil 2021^3^ | AMP Rapid Test SARS-CoV-2 Ag  vs Assut Europe COVID-19 Ag | NA/NA | 100% Nasopharyngeal | 40.8 | 100% General Public |
| Abusrewil 2021^3^ | AMP Rapid Test SARS-CoV-2 Ag  vs Biocredit Covid-19 Ag | NA/NA | 100% Nasopharyngeal | 40.8 | 100% General Public |
| Abusrewil 2021^3^ | AMP Rapid Test SARS-CoV-2 Ag  vs Bioperfectus SARS-CoV -2 Ag Rapid Test Kit | NA/NA | 100% Nasopharyngeal | 40.8 | 100% General Public |
| Abusrewil 2021^3^ | AMP Rapid Test SARS-CoV-2 Ag  vs Certest Biotec SARS-CoV-2 Ag | NA/NA | 100% Nasopharyngeal | 40.8 | 100% General Public |
| Baro 2021^2^ | Lepu Medical SARS-CoV-2 antigen test vs PanBio COVID-19 Ag Rapid test (Abbott) | 100% / 0% | 100% Nasopharyngeal | NA | NA |
| Baro 2021^2^ | Lepu Medical SARS-CoV-2 antigen test vs SARS-CoV-2 Rapid Antigen Test (Roche) | 100% / 0% | 100% Nasopharyngeal | NA | NA |
| Hirotsu 2021^5^ | Lumipulse SARS-CoV-2 Ag vs SARS-CoV-2 Rapid Antigen Test (Roche) | NA/NA | 100% Nasopharyngeal | NA | NA |
| Abusrewil 2021^3^ | Orient Gene Rapid Covid-19 Ag vs PanBio COVID-19 Ag Rapid test (Abbott) | NA/NA | 100% Nasopharyngeal | 40.8 | 100% General Public |
| Favresse 2021^4^ | Ortho’s VITROS SARS-CoV-2 Ag Test vs PanBio COVID-19 Ag Rapid test (Abbott) | NA/NA | 100% Nasopharyngeal | 55.5 | 100% General Public |
| Favresse 2021^4^ | Ortho’s VITROS SARS-CoV-2 Ag Test vs SARS-CoV-2 Rapid Antigen Test (Roche) | NA/NA | 100% Nasopharyngeal | 55.5 | 100% General Public |
| Baro 2021^2^; Favresse 2021^4^ | PanBio COVID-19 Ag Rapid test (Abbott) vs SARS-CoV-2 Rapid Antigen Test (Roche) | 100% / 0% | 100% Nasopharyngeal | NA | 100% General Public |
| Jaaskelainen 2021^6^ | PanBio COVID-19 Ag Rapid test (Abbott) vs Sofia SARS Antigen FIA | 0% / 100% | 100% Mixed | NA | 100% General Public |
| Pérez‐García 2021^7^ | PanBio COVID-19 Ag Rapid test (Abbott) vs Standard F COVID-19 Ag | NA/NA | 100% Nasopharyngeal | NA | NA |
| Berger 2021^8^; Jaaskelainen 2021^6^ | PanBio COVID-19 Ag Rapid test (Abbott) vs Standard Q COVID-19 Ag | 0% / 100% | 50% Nasopharyngeal / 50% Mixed | 36.7 | 100% General Public |
| Abusrewil 2021^3^ | Assut Europe COVID-19 Ag vs Espline SARS-CoV-2 rapid antigen test | NA/NA | 100% Nasopharyngeal | 40.8 | 100% General Public |
| Abusrewil 2021^3^ | Assut Europe COVID-19 Ag vs Flowflex COVID-19 Ag | NA/NA | 100% Nasopharyngeal | 40.8 | 100% General Public |
| Abusrewil 2021^3^ | Assut Europe COVID-19 Ag vs Fluorecare SARS-CoV--2 spike protein | NA/NA | 100% Nasopharyngeal | 40.8 | 100% General Public |
| Abusrewil 2021^3^ | Assut Europe COVID-19 Ag vs Orient Gene Rapid Covid-19 Ag | NA/NA | 100% Nasopharyngeal | 40.8 | 100% General Public |
| Abusrewil 2021^3^ | Assut Europe COVID-19 Ag vs PanBio COVID-19 Ag Rapid test (Abbott) | NA/NA | 100% Nasopharyngeal | 40.8 | 100% General Public |
| Abusrewil 2021^3^ | Assut Europe COVID-19 Ag vs Biocredit Covid-19 Ag | NA/NA | 100% Nasopharyngeal | 40.8 | 100% General Public |
| Abusrewil 2021^3^ | Assut Europe COVID-19 Ag vs Bioperfectus SARS-CoV -2 Ag Rapid Test Kit | NA/NA | 100% Nasopharyngeal | 40.8 | 100% General Public |
| Abusrewil 2021^3^ | Assut Europe COVID-19 Ag vs Certest Biotec SARS-CoV-2 Ag | NA/NA | 100% Nasopharyngeal | 40.8 | 100% General Public |
| Koeleman 2021^9^ | Romed COVID-19 Ag vs SARS-CoV-2 Rapid Antigen Test (Roche) | 0% / 100% | 100% Nasopharyngeal | NA | NA |
| Porte 2021^10^ | Sofia SARS Antigen FIA vs Standard F COVID-19 Ag | NA/NA | 100% Mixed | 39 | NA |
| Jaaskelainen 2021^6^ | Sofia SARS Antigen FIA vs Standard Q COVID-19 Ag | 0% / 100% | 100% Mixed | NA | 100% General Public |
| Schuit 2021^11^ | BD Veritor Ag Test vs Standard Q COVID-19 Ag | NA/NA | 100% Oropharyngeal | 43.3 | 100% General Public |
| Abusrewil 2021^3^ | Biocredit Covid-19 Ag vs Espline SARS-CoV-2 rapid antigen test | NA/NA | 100% Nasopharyngeal | 40.8 | 100% General Public |
| Abusrewil 2021^3^ | Biocredit Covid-19 Ag vs Flowflex COVID-19 Ag | NA/NA | 100% Nasopharyngeal | 40.8 | 100% General Public |
| Abusrewil 2021^3^ | Biocredit Covid-19 Ag vs Fluorecare SARS-CoV--2 spike protein | NA/NA | 100% Nasopharyngeal | 40.8 | 100% General Public |
| Abusrewil 2021^3^ | Biocredit Covid-19 Ag vs Orient Gene Rapid Covid-19 Ag | NA/NA | 100% Nasopharyngeal | 40.8 | 100% General Public |
| Abusrewil 2021^3^ | Biocredit Covid-19 Ag vs PanBio COVID-19 Ag Rapid test (Abbott) | NA/NA | 100% Nasopharyngeal | 40.8 | 100% General Public |
| Abusrewil 2021^3^ | Biocredit Covid-19 Ag vs Bioperfectus SARS-CoV -2 Ag Rapid Test Kit | NA/NA | 100% Nasopharyngeal | 40.8 | 100% General Public |
| Abusrewil 2021^3^ | Biocredit Covid-19 Ag vs Certest Biotec SARS-CoV-2 Ag | NA/NA | 100% Nasopharyngeal | 40.8 | 100% General Public |
| Abusrewil 2021^3^ | Bioperfectus SARS-CoV -2 Ag Rapid Test Kit vs Espline SARS-CoV-2 rapid antigen test | NA/NA | 100% Nasopharyngeal | 40.8 | 100% General Public |
| Abusrewil 2021^3^ | Bioperfectus SARS-CoV -2 Ag Rapid Test Kit vs Flowflex COVID-19 Ag | NA/NA | 100% Nasopharyngeal | 40.8 | 100% General Public |
| Abusrewil 2021^3^ | Bioperfectus SARS-CoV -2 Ag Rapid Test Kit vs Fluorecare SARS-CoV--2 spike protein | NA/NA | 100% Nasopharyngeal | 40.8 | 100% General Public |
| Abusrewil 2021^3^ | Bioperfectus SARS-CoV -2 Ag Rapid Test Kit vs Orient Gene Rapid Covid-19 Ag | NA/NA | 100% Nasopharyngeal | 40.8 | 100% General Public |
| Abusrewil 2021^3^ | Bioperfectus SARS-CoV -2 Ag Rapid Test Kit vs PanBio COVID-19 Ag Rapid test (Abbott) | NA/NA | 100% Nasopharyngeal | 40.8 | 100% General Public |
| Abusrewil 2021^3^ | Bioperfectus SARS-CoV -2 Ag Rapid Test Kit vs Certest Biotec SARS-CoV-2 Ag | NA/NA | 100% Nasopharyngeal | 40.8 | 100% General Public |
| Favresse 2021^4^ | Biotical SARS-CoV-2 Ag card test vs Healgen COVID-19 Ag Test | NA/NA | 100% Nasopharyngeal | 55.5 | 100% General Public |
| Favresse 2021^4^ | Biotical SARS-CoV-2 Ag card test vs Ortho’s VITROS SARS-CoV-2 Ag Test | NA/NA | 100% Nasopharyngeal | 55.5 | 100% General Public |
| Favresse 2021^4^ | Biotical SARS-CoV-2 Ag card test vs PanBio COVID-19 Ag Rapid test (Abbott) | NA/NA | 100% Nasopharyngeal | 55.5 | 100% General Public |
| Favresse 2021^4^ | Biotical SARS-CoV-2 Ag card test vs SARS-CoV-2 Rapid Antigen Test (Roche) | NA/NA | 100% Nasopharyngeal | 55.5 | 100% General Public |
| Abusrewil 2021^3^ | Certest Biotec SARS-CoV-2 Ag vs Espline SARS-CoV-2 rapid antigen test | NA/NA | 100% Nasopharyngeal | 40.8 | 100% General Public |
| Abusrewil 2021^3^ | Certest Biotec SARS-CoV-2 Ag vs Flowflex COVID-19 Ag | NA/NA | 100% Nasopharyngeal | 40.8 | 100% General Public |
| Abusrewil 2021^3^ | Certest Biotec SARS-CoV-2 Ag vs Fluorecare SARS-CoV--2 spike protein | NA/NA | 100% Nasopharyngeal | 40.8 | 100% General Public |
| Abusrewil 2021^3^ | Certest Biotec SARS-CoV-2 Ag vs Orient Gene Rapid Covid-19 Ag | NA/NA | 100% Nasopharyngeal | 40.8 | 100% General Public |
| Abusrewil 2021^3^; Perez-García 2021^12^ | Certest Biotec SARS-CoV-2 Ag vs PanBio COVID-19 Ag Rapid test (Abbott) | NA/NA | 100% Nasopharyngeal | 45.9 | 100% General Public |
| Koeleman 2021^9^ | Certest Biotec SARS-CoV-2 Ag vs Romed COVID-19 Ag | 0% / 100% | 100% Nasopharyngeal | NA | NA |
| Koeleman 2021^9^ | Certest Biotec SARS-CoV-2 Ag vs SARS-CoV-2 Rapid Antigen Test (Roche) | 0% / 100% | 100% Nasopharyngeal | NA | NA |
| Baro 2021^2^ | Clinitest Rapid COVID-19 Antigen Self-Test vs COVID-19 Rapid Antigen Test (Surescreen) | 100% / 0% | 100% Nasopharyngeal | NA | NA |
| Baro 2021^2^ | Clinitest Rapid COVID-19 Antigen Self-Test vs Lepu Medical SARS-CoV-2 antigen test | 100% / 0% | 100% Nasopharyngeal | NA | NA |
| Baro 2021^2^ | Clinitest Rapid COVID-19 Antigen Self-Test vs PanBio COVID-19 Ag Rapid test (Abbott) | 100% / 0% | 100% Nasopharyngeal | NA | NA |
| Baro 2021^2^ | Clinitest Rapid COVID-19 Antigen Self-Test vs SARS-CoV-2 Rapid Antigen Test (Roche) | 100% / 0% | 100% Nasopharyngeal | NA | NA |
| ***Rapid Molecular Tests*** | | | | | |
| Mahmoud 2021^13^ | Cobas Liat SARS-CoV-2 and influenza A/B nucleic acid test vs iAMP COVID-19 Detection Kit | NA/NA | 100% Nasopharyngeal | NA | NA |
| Mahmoud 2021^13^ | Cobas Liat SARS-CoV-2 and influenza A/B nucleic acid test vs AQ-TOP COVID-19 Rapid Detection Kit Plus | NA/NA | 100% Nasopharyngeal | NA | NA |
| Mahmoud 2021^13^ | Cobas Liat SARS-CoV-2 and influenza A/B nucleic acid test vs Genechecker PCR system‐UF 300–RT PCR system | NA/NA | 100% Nasopharyngeal | NA | NA |
| Mahmoud 2021^13^ | Cobas Liat SARS-CoV-2 and influenza A/B nucleic acid test vs POCKIT Central SARS-CoV-2 (orf 1ab) | NA/NA | 100% Nasopharyngeal | NA | NA |
| Mahmoud 2021^13^ | Cobas Liat SARS-CoV-2 and influenza A/B nucleic acid test vs ID Now COVID-19 | NA/NA | 100% Nasopharyngeal | NA | NA |
| Mahmoud 2021^13^ | iAMP COVID-19 Detection Kit vs AQ-TOP COVID-19 Rapid Detection Kit Plus | NA/NA | 100% Nasopharyngeal | NA | NA |
| Mahmoud 2021^13^ | iAMP COVID-19 Detection Kit vs Genechecker PCR system‐UF 300–RT PCR system | NA/NA | 100% Nasopharyngeal | NA | NA |
| Mahmoud 2021^13^ | iAMP COVID-19 Detection Kit vs POCKIT Central SARS-CoV-2 (orf 1ab) | NA/NA | 100% Nasopharyngeal | NA | NA |
| Mahmoud 2021^13^ | iAMP COVID-19 Detection Kit vs ID Now COVID-19 | NA/NA | 100% Nasopharyngeal | NA | NA |
| Mahmoud 2021^13^ | AQ-TOP COVID-19 Rapid Detection Kit Plus vs Genechecker PCR system‐UF 300–RT PCR system | NA/NA | 100% Nasopharyngeal | NA | NA |
| Mahmoud 2021^13^ | AQ-TOP COVID-19 Rapid Detection Kit Plus vs POCKIT Central SARS-CoV-2 (orf 1ab) | NA/NA | 100% Nasopharyngeal | NA | NA |
| Mahmoud 2021^13^ | AQ-TOP COVID-19 Rapid Detection Kit Plus vs ID Now COVID-19 | NA/NA | 100% Nasopharyngeal | NA | NA |
| Mahmoud 2021^13^ | Genechecker PCR system‐UF 300–RT PCR system vs POCKIT Central SARS-CoV-2 (orf 1ab) | NA/NA | 100% Nasopharyngeal | NA | NA |
| Mahmoud 2021^13^ | Genechecker PCR system‐UF 300–RT PCR system vs ID Now COVID-19 | NA/NA | 100% Nasopharyngeal | NA | NA |
| Mahmoud 2021^13^ | POCKIT Central SARS-CoV-2 (orf 1ab) vs ID Now COVID-19 | NA/NA | 100% Nasopharyngeal | NA | NA |
| Dust 2020^14^ | Cobas SARS-CoV-2 Nucleic Acid Test vs Xpert Xpress | NA/NA | 100% Nasopharyngeal | NA | NA |
| Fowler 2021^15^ | Direct RT-LAMP vs RNA RT-LAMP | NA/NA | 100% Mixed | NA | 100% Health-care workers/Mixed |
| Jokela 2020^16^ | Mobidiag Novodiag Covid-19 vs Xpert Xpress | NA/NA | 100% Mixed | 74 | 100% General Public |

**References**

1. Seynaeve Y, Heylen J, Fontaine C, et al. Evaluation of two rapid antigenic tests for the detection of SARS-CoV-2 in nasopharyngeal swabs. *J Clin Med* 2021;10(13):2774.

2. Baro B, Rodo P, Ouchi D, et al. Performance characteristics of five antigen-detecting rapid diagnostic test (Ag-RDT) for SARS-CoV-2 asymptomatic infection: a head-to-head benchmark comparison. *J Infect* 2021;82(6):269-75.

3. Abusrewil Z, Alhudiri IM, Kaal HH, et al. Time scale performance of rapid antigen testing for SARS‐CoV‐2: Evaluation of 10 rapid antigen assays. *J Med Virol* 2021;93(12):6512-18.

4. Favresse J, Gillot C, Oliveira M, et al. Head-to-head comparison of rapid and automated antigen detection tests for the diagnosis of SARS-CoV-2 infection. *Journal of clinical medicine* 2021;10(2):265.

5. Hirotsu Y, Sugiura H, Maejima M, et al. Comparison of Roche and Lumipulse quantitative SARS-CoV-2 antigen test performance using automated systems for the diagnosis of COVID-19. *J Clin Med* 2021;108:263-69.

6. Jaaskelainen A, Ahava MJ, Jokela P, et al. Evaluation of three rapid lateral flow antigen detection tests for the diagnosis of SARS-CoV-2 infection. *J Clin Virol* 2021;137:104785.

7. Pérez‐García F, Romanyk J, Moya Gutiérrez H, et al. Comparative evaluation of Panbio and SD Biosensor antigen rapid diagnostic tests for COVID‐19 diagnosis. *J Med Virol* 2021;93(9):5650-54.

8. Berger A, Nsoga MTN, Perez-Rodriguez FJ, et al. Diagnostic accuracy of two commercial SARS-CoV-2 antigen-detecting rapid tests at the point of care in community-based testing centers. *PLoS One* 2021;16(3):e0248921.

9. Koeleman JG, Brand H, de Man SJ, et al. Clinical evaluation of rapid point-of-care antigen tests for diagnosis of SARS-CoV-2 infection. *Eur J Clin Microbiol Infect Dis* 2021;40(9):1975-81.

10. Porte L, Legarraga P, Iruretagoyena M, et al. Evaluation of two fluorescence immunoassays for the rapid detection of SARS-CoV-2 antigen—new tool to detect infective COVID-19 patients. *PeerJ* 2021;9:e10801.

11. Schuit E, Veldhuijzen IK, Venekamp RP, et al. Diagnostic accuracy of rapid antigen tests in asymptomatic and presymptomatic close contacts of individuals with confirmed SARS-CoV-2 infection: cross sectional study. *BMJ* 2021;374:n1676.

12. Pérez-García F, Romanyk J, Gómez-Herruz P, et al. Diagnostic performance of CerTest and Panbio antigen rapid diagnostic tests to diagnose SARS-CoV-2 infection. *J Clin Virol* 2021;137:104781.

13. Mahmoud SA, Ganesan S, Ibrahim E, et al. Evaluation of six different rapid methods for nucleic acid detection of SARS‐COV‐2 virus. *J Med Virol* 2021;93(9):5538-43.

14. Dust K, Hedley A, Nichol K, et al. Comparison of commercial assays and laboratory developed tests for detection of SARS-CoV-2. *J Virol Methods* 2020;285:113970.

15. Fowler VL, Armson B, Gonzales JL, et al. A highly effective reverse-transcription loop-mediated isothermal amplification (RT-LAMP) assay for the rapid detection of SARS-CoV-2 infection. *J Infect* 2021;82(1):117-25.

16. Jokela P, Jaaskelainen AE, Jarva H, et al. SARS-CoV-2 sample-to-answer nucleic acid testing in a tertiary care emergency department: evaluation and utility. *J Clin Virol* 2020;131:104614.

# Appendix 9. Network meta-analysis results for rapid antigen tests, and rapid molecular tests.

| **Test** | **Manufacturer** | **Sensitivity  (95% CI)*** | **Specificity (95%CI)*** |
| --- | --- | --- | --- |
| **Rapid Antigen Tests** | | | |
| Accucare COVID-19 Antigen Card Test | Lab-Care Diagnostics (India) Pvt. Ltd | 0.43 (0.08, 0.86) | 0.94 (0.35, 1.00) |
| AMP Rapid Test SARS-CoV-2 Ag | AMP Diagnostics | 0.72 (0.21, 0.98) | 0.83 (0.27, 1.00) |
| Assut Europe COVID-19 Ag | Assut Europe | 0.62 (0.16, 0.95) | 0.88 (0.3, 1.00) |
| BD Veritor Ag Test | Bseckton Dickinson | 0.60 (0.16, 0.92) | 0.94 (0.34, 1.00) |
| Biocredit Covid-19 Ag | RapiGEN Inc | 0.65 (0.29, 0.89) | 0.96 (0.42, 1.00) |
| Bioperfectus SARS-CoV -2 Ag Rapid Test Kit | Bioperfectus technologies | 0.67 (0.18, 0.96) | 0.78 (0.29, 1.00) |
| Biotical SARS-CoV-2 Ag card test | Biotical Health | 0.60 (0.18, 0.91) | 0.88 (0.31, 1.00) |
| Certest Biotec SARS-CoV-2 Ag | Certest Biotec | 0.60 (0.35, 0.78) | **0.97 (0.56, 1.00)** |
| Clinitest Rapid COVID-19 Antigen Self-Test | Siemens Healthineers | 0.75 (0.35, 0.93) | 0.95 (0.52, 0.99) |
| Coris BioConcept COVID-19 Ag Respi-Strip | Coris BioConcept | 0.46 (0.25, 0.67) | **0.99 (0.65, 1.00)** |
| COVID-19 Rapid Antigen Test (Surescreen) | Surescreen | 0.53 (0.13, 0.89) | 0.85 (0.31, 0.99) |
| COVID-VIRO | AAZ-LMB | **0.93 (0.48, 0.99)** | **0.98 (0.44, 1.00)** |
| Dräger Antigen Test SARS-CoV-2 | Dräger | **0.89 (0.29, 1.00)** | 0.87 (0.29, 0.99) |
| Espline SARS-CoV-2 rapid antigen test | Fujirebio Inc., Japan | 0.58 (0.22, 0.87) | 0.89 (0.41, 0.99) |
| Flowflex COVID-19 Ag | Acon Laboratories | **0.88 (0.32, 1.00)** | 0.84 (0.28, 1.00) |
| Fluorecare SARS-CoV--2 spike protein | Shenzhen Microprofit Biotech Co | 0.76 (0.24, 0.99) | 0.84 (0.27, 1.00) |
| GenBody COVID-19 Ag | Meridian Bioscience | **0.85 (0.37, 0.97)** | 0.94 (0.45, 1.00) |
| Healgen COVID-19 Ag Test | Healgen Scientific | 0.74 (0.44, 0.89) | 0.93 (0.53, 0.99) |
| Indicaid COVID-19 Rapid Antigen Test | Phase Scientifc International | 0.78 (0.47, 0.92) | 0.93 (0.51, 0.99) |
| Inflammacheck Device for rapid Ag detection | Exhalation Medical Technology | 0.79 (0.23, 0.99) | 0.88 (0.31, 1.00) |
| Innova Medical Group SARS-CoV-2 Ag Test | Innova | 0.46 (0.09, 0.88) | 0.96 (0.36, 1.00) |
| Lepu Medical SARS-CoV-2 antigen test | Lepu Medical | 0.63 (0.18, 0.92) | 0.74 (0.24, 0.96) |
| Liaison SARS-CoV-2 Ag | DiaSorin | 0.71 (0.39, 0.89) | **0.98 (0.58, 1.00)** |
| Lumipulse SARS-CoV-2 Ag | Fujirebio | 0.78 (0.52, 0.89) | 0.94 (0.73, 0.99) |
| Orient Gene Rapid Covid-19 Ag | Orient Gene/Healgen Biotech | 0.49 (0.11, 0.88) | 0.82 (0.29, 1.00) |
| Ortho’s VITROS SARS-CoV-2 Ag Test | Ortho Clinical Diagnostics | **0.87 (0.33, 1.00)** | 0.90 (0.34, 1.00) |
| PanBio COVID-19 Ag Rapid test (Abbott) | Abbott | 0.67 (0.59, 0.75) | **0.99 (0.97, 0.99)** |
| QuickNavi-COVID19 Ag - FIND | Otsuka Pharmaceutical Co., Ltd. | 0.73 (0.21, 0.97) | 0.83 (0.34, 1.00) |
| Rapid Test Ag 2019-nCoV (Prognosis, Biotech) | Prognosis, Biotech | 0.73 (0.21, 0.97) | 0.93 (0.34, 1.00) |
| RIDA QUICK SARS-CoV-2 Antigen test | R-Biopharm AG | 0.65 (0.18, 0.95) | 0.86 (0.31, 1.00) |
| Romed COVID-19 Ag | Romed | 0.66 (0.2, 0.93) | 0.90 (0.35, 1.00) |
| SARS-CoV-2 Ag Test on the LumiraDx Platform | LumiraDx | 0.77 (0.51, 0.9) | 0.92 (0.70, 0.98) |
| SARS-CoV-2 Rapid Antigen Test (Roche) | Roche | 0.71 (0.56, 0.82) | 0.96 (0.78, 0.99) |
| Sofia SARS Antigen FIA | Quidel | **0.81 (0.46, 0.95)** | **0.97 (0.71, 0.99)** |
| Standard F COVID-19 Ag | SD-Biosensor | 0.67 (0.46, 0.80) | 0.94 (0.80, 0.98) |
| Standard Q COVID-19 Ag | SD-Biosensor | 0.66 (0.52, 0.76) | **0.98 (0.95, 0.99)** |
| **Rapid Molecular Tests** | | | |
| AQ-TOP COVID-19 Rapid Detection Kit Plus | Seasun Biomaterials | **0.89 (0.30, 1.00)** | 0.83 (0.29, 0.99) |
| Biomeme SARS-CoV-2 Real-Time RT-PCR Test | Biomeme | **0.92 (0.32, 1.00)** | 0.86 (0.30, 1.00) |
| Cobas Liat SARS-CoV-2 and influenza A/B nucleic acid test | Roche | **0.86 (0.39, 1.00)** | 0.94 (0.53, 0.99) |
| Cobas SARS-CoV-2 Nucleic Acid Test | Roche | **0.89 (0.31, 1.00)** | 0.88 (0.34, 1.00) |
| Direct RT-LAMP | Optigene Ltd. | 0.60 (0.15, 0.92) | 0.83 (0.26, 0.99) |
| Genechecker PCR system‐UF 300–RT PCR system | Biomedal Ivydal | **0.82 (0.25, 0.99)** | 0.91 (0.34, 1.00) |
| GeneSoC | Kyorin Pharmaceutical Co. Ltd | **0.89 (0.33, 1.00)** | 0.88 (0.33, 1.00) |
| iAMP COVID-19 Detection Kit | Atila BioSystems | 0.45 (0.10, 0.86) | 0.84 (0.30, 0.99) |
| ID Now COVID-19 | Abbott | **0.82 (0.54, 0.94)** | 0.96 (0.67, 0.99) |
| Idylla SARS-CoV-2 Test - Biocartis | Biocartis | **0.82 (0.26, 0.99)** | 0.87 (0.32, 1.00) |
| Isopollo COVID-19 detection kit | M Monitor | 0.57 (0.14, 0.92) | 0.89 (0.34, 1.00) |
| Loopamp SARS-CoV-2 Detection Kit | Eiken Chemical, Tokyo, Japan | 0.73 (0.30, 0.95) | 0.70 (0.26, 0.95) |
| MatMaCorp COVID-19 2SF | MatMaCorp | 0.73 (0.21, 0.97) | 0.90 (0.32, 1.00) |
| MicrosensDx RapiPrep® SARS-CoV-2 | MicrosensDx | 0.69 (0.18, 0.97) | 0.66 (0.16, 0.96) |
| Mobidiag Novodiag Covid-19 | Mobidiag | **0.80 (0.34, 0.97)** | **0.97 (0.47, 1.00)** |
| POCKIT Central SARS-CoV-2 (orf 1ab) | GeneReach Biotechnology Corp | 0.77 (0.22, 0.97) | 0.93 (0.34, 1.00) |
| QiaStat-Dx Respiratory SARS-CoV-2 Panel | Qiagen | **0.90 (0.31, 1.00)** | 0.79 (0.25, 0.99) |
| RNA RT-LAMP | Optigene | **0.86 (0.28, 0.99)** | 0.88 (0.30, 1.00) |
| SAMBA II SARS-CoV-2 Test | DRW: Diagnostics for the Real World | **0.87 (0.29, 1.00)** | 0.91 (0.30, 1.00) |
| SARS-CoV-2 Rapid Colorimetric LAMP Assay Kit | New England BioLabs | 0.67 (0.29, 0.89) | **0.98 (0.46, 1.00)** |
| Simplexa COVID-19 Direct Kit - DiaSorin | DiaSorin | **0.89 (0.33, 1.00)** | 0.82 (0.27, 0.99) |
| Truenat Beta CoV - Molbio Diagnostics | Molbio Diagnostics | **0.90 (0.31, 1.00)** | 0.86 (0.30, 1.00) |
| Xpert Xpress | Cepheid | **0.99 (0.83, 1.00)** | **0.97 (0.69, 1.00)** |

*** Sensitivity estimates above or equal to 80% and specificities above or equal to 97% are highlighted in bold

# Appendix 10. Within-test heterogeneity variance for rapid molecular tests in DTA-NMA

| **Rapid molecular test** | | **Within-test heterogeneity** | |
| --- | --- | --- | --- |
|  |  | **Sensitivity** | **Specificity** |
| AQ-TOP COVID-19 Rapid Detection Kit Plus | 0.01 (3.25, 195.57) | | 0.01 (4.39, 343.35) |
| Biomeme SARS-CoV-2 Real-Time RT-PCR Test | 0.01 (3.97, 290.08) | | 0.01 (3.98, 2205.76) |
| Cobas Liat SARS-CoV-2 and influenza A/B nucleic acid test | 0.13 (11.82, 605.26) | | 0.00 (1.05, 36.69) |
| Cobas SARS-CoV-2 Nucleic Acid Test | 0.03 (7.40, 794.77) | | 0.03 (8.91, 862.06) |
| Direct RT-LAMP | 0.01 (3.20, 194.98) | | 0.01 (3.76, 181.71) |
| Genechecker PCR system?UF 300–RT PCR system | 0.01 (3.62, 255.93) | | 0.01 (4.34, 250.22) |
| GeneSoC | 0.04 (15.46, 1359.57) | | 0.04 (19.87, 3964.29) |
| iAMP COVID-19 Detection Kit | 0.01 (3.23, 126.66) | | 0.01 (3.74, 220.94) |
| ID Now COVID-19 | 0.01 (1.69, 15.31) | | 0.01 (2.30, 45.72) |
| Idylla SARS-CoV-2 Test - Biocartis | 0.01 (3.37, 149.86) | | 0.02 (9.10, 5084.44) |
| Isopollo COVID-19 detection kit | 0.02 (3.83, 184.96) | | 0.03 (11.40, 1104.12) |
| Loopamp SARS-CoV-2 Detection Kit | 0.12 (6.11, 105.23) | | 0.03 (7.00, 125.85) |
| MatMaCorp COVID-19 2SF | 0.01 (3.33, 146.46) | | 0.02 (7.35, 1278.03) |
| MicrosensDx RapiPrep® SARS-CoV-2 | 0.01 (3.57, 174.73) | | 0.01 (2.80, 138.50) |
| Mobidiag Novodiag Covid-19 | 0.00 (1.64, 62.50) | | 0.02 (2.90, 288.85) |
| POCKIT Central SARS-CoV-2 (orf 1ab) | 0.01 (2.71, 93.20) | | 0.01 (4.50, 300.61) |
| QiaStat-Dx Respiratory SARS-CoV-2 Panel | 0.02 (9.86, 1054.30) | | 0.02 (3.19, 198.66) |
| RNA RT-LAMP | 0.01 (3.47, 140.76) | | 0.01 (4.59, 335.04) |
| SAMBA II SARS-CoV-2 Test | 0.01 (3.32, 160.84) | | 0.01 (3.74, 172.56) |
| SARS-CoV-2 Rapid Colorimetric LAMP Assay Kit | 0.00 (0.80, 33.62) | | 0.01 (5.30, 589.45) |
| Simplexa COVID-19 Direct Kit - DiaSorin | 0.03 (13.45, 1754.13) | | 0.01 (3.81, 212.62) |
| Truenat Beta CoV - Molbio Diagnostics | 0.02 (9.44, 1133.41) | | 0.04 (11.38, 2581.98) |
| Xpert Xpress | 0.00 (1.06, 31.73) | | 0.02 (4.06, 119.32) |

# Appendix 11. Within-test heterogeneity variance for rapid antigen tests in DTA-NMA

| **Rapid antigen test** | **Within-test heterogeneity** | |
| --- | --- | --- |
|  | **Sensitivity** | **Specificity** |
| Accucare COVID-19 Antigen Card Test | 2.99 (0.01, 304.8) | 5.44 (0.02, 344.68) |
| AMP Rapid Test SARS-CoV-2 Ag | 3.36 (0.01, 217.16) | 5.22 (0.01, 651.10) |
| Assut Europe COVID-19 Ag | 3.22 (0.01, 111.09) | 6.10 (0.01, 462.82) |
| BD Veritor Ag Test | 3.17 (0.00, 148.97) | 4.33 (0.00, 395.86) |
| Biocredit Covid-19 Ag | 1.49 (0.01, 54.88) | 6.04 (0.02, 826.58) |
| Bioperfectus SARS-CoV -2 Ag Rapid Test Kit | 3.39 (0.00, 159.47) | 8.72 (0.02, 9387.93) |
| Biotical SARS-CoV-2 Ag card test | 3.19 (0.01, 136.38) | 4.03 (0.01, 221.05) |
| Certest Biotec SARS-CoV-2 Ag | 0.25 (0.00, 9.00) | 2.52 (0.01, 102.04) |
| Clinitest Rapid COVID-19 Antigen Self-Test | 1.68 (0.00, 55.14) | 1.32 (0.00, 47.48) |
| Coris BioConcept COVID-19 Ag Respi-Strip | 0.28 (0.00, 6.37) | 2.51 (0.01, 123.27) |
| COVID-19 Rapid Antigen Test (Surescreen) | 3.32 (0.01, 137.05) | 5.02 (0.01, 3017.75) |
| COVID-VIRO | 1.04 (0.00, 41.96) | 6 (0.01, 605.43) |
| Drδger Antigen Test SARS-CoV-2 | 4.13 (0.02, 194.18) | 2.56 (0.01, 97.60) |
| Espline SARS-CoV-2 rapid antigen test | 2.19 (0.01, 125.91) | 2.15 (0.00, 127.30) |
| Flowflex COVID-19 Ag | 8.95 (0.02, 1123.14) | 5.64 (0.01, 603.12) |
| Fluorecare SARS-CoV--2 spike protein | 4.41 (0.02, 398.38) | 5.51 (0.01, 424.91) |
| GenBody COVID-19 Ag | 1.29 (0.00, 57.46) | 4.18 (0.01, 221.37) |
| Healgen COVID-19 Ag Test | 0.48 (0.00, 14.60) | 3.42 (0.01, 90.16) |
| Indicaid COVID-19 Rapid Antigen Test | 0.34 (0.00, 12.21) | 3.71 (0.04, 63.13) |
| Inflammacheck Device for rapid Ag detection | 3.62 (0.01, 131.12) | 4.36 (0.02, 232.82) |
| Innova Medical Group SARS-CoV-2 Ag Test | 3.18 (0.02, 129.54) | 5.86 (0.02, 268.78) |
| Lepu Medical SARS-CoV-2 antigen test | 3.23 (0.01, 397.32) | 4.38 (0.02, 464.46) |
| Liaison SARS-CoV-2 Ag | 0.46 (0.00, 18.90) | 2.45 (0.00, 113.90) |
| Lumipulse SARS-CoV-2 Ag | 0.32 (0.00, 7.74) | 0.55 (0.00, 11.72) |
| Orient Gene Rapid Covid-19 Ag | 3.25 (0.02, 184.41) | 6.93 (0.01, 5050.94) |
| Ortho’s VITROS SARS-CoV-2 Ag Test | 14.17 (0.03, 17399.37) | 10.36 (0.04, 763.65) |
| PanBio COVID-19 Ag Rapid test (Abbott) | 0.08 (0.00, 1.05) | 0.72 (0.00, 5.58) |
| QuickNavi-COVID19 Ag - FIND | 2.75 (0.01, 156.70) | 26.45 (0.10, 2618.37) |
| Rapid Test Ag 2019-nCoV (Prognosis, Biotech) | 2.47 (0.00, 110.19) | 5.08 (0.01, 433.39) |
| RIDA QUICK SARS-CoV-2 Antigen test | 3.34 (0.01, 1453.58) | 7.12 (0.02, 909.43) |
| Romed COVID-19 Ag | 3.50 (0.01, 245.14) | 12.01 (0.02, 2073.88) |
| SARS-CoV-2 Ag Test on the LumiraDx Platform | 0.33 (0.00, 7.14) | 0.77 (0.00, 12.93) |
| SARS-CoV-2 Rapid Antigen Test (Roche) | 0.38 (0.00, 5.48) | 3.26 (0.03, 26.70) |
| Sofia SARS Antigen FIA | 1.28 (0, 45.86) | 0.81 (0.00, 18.96) |
| Standard F COVID-19 Ag | 0.30 (0.00, 5.58) | 0.36 (0.00, 6.37) |
| Standard Q COVID-19 Ag | 0.78 (0.00, 4.59) | 0.51 (0.00, 5.47) |

# Appendix 12. Total between-study heterogeneity variance in DTA-NMA

|  | **Between-study heterogeneity** | |
| --- | --- | --- |
|  | **Sensitivity** | **Specificity** |
| Rapid molecular tests | 0.78 (0.00, 7.25) | 1.58 (0.00, 23.54) |
| Rapid antigen tests | 1.02 (0.42, 1.91) | 2.17 (0.80, 4.54) |

# Appendix 13. Subgroup analysis results using DTA meta-analysis model by participant type, test sample type, molecular test type, and symptoms

|  |  |  |  | **Summary Estimates** | | **Heterogeneity Standard Deviation** | |
| --- | --- | --- | --- | --- | --- | --- | --- |
| **Subgroup** | | **Test Type** | **# Studies  (# patients)** | **Sensitivity (95% CI)** | **Specificity (95% CI)** | **Sensitivity** | **Specificity** |
| ***Participant type*** | **General Public** | Rapid Molecular Test | 16 (2678) | 0.92  (0.83, 0.97) | 0.98  (0.97, 0.99) | 1.58 | 0.64 |
|  | **Healthcare Workers-Mixed** |  | 2 (315) | - | - | - | - |
| ***Test Sample Type*** | **Combined Sample*** |  | 12 (2324) | 0.94  (0.87, 0.98) | 0.98  (0.95, 0.99) | 1.39 | 1.04 |
|  | **Nasopharyngeal Sample** |  | 21 (7916) | 0.91  (0.84, 0.96) | 0.98  (0.96, 0.99) | 1.54 | 1.30 |
|  | **Saliva** |  | 1 (209) | 0.96  (0.92, 0.99) | 0.99  (0.95, 1.00) | - | - |
| ***Test Type*** | **rRT-PCR** |  | 17 (5404) | 0.97  (0.95, 0.99) | 0.98  (0.97, 0.99) | 0.80 | 0.66 |
|  | **RT-Isothermal (Other)** |  | 8 (3808) | 0.86  (0.71, 0.94) | 0.99  (0.97, 0.99) | 1.29 | 0.88 |
|  | **RT-LAMP** |  | 9 (1237) | 0.84  (0.67, 0.93) | 0.96  (0.87, 0.99) | 1.40 | 1.77 |
| ***Participant type*** | **General Public** | Rapid Antigen Test | 58 (58651) | 0.79  (0.73, 0.84) | 0.99  (0.98, 0.99) | 1.19 | 1.55 |
|  | **Healthcare Workers-Mixed** |  | 7 (25827) | 0.71  (0.54, 0.84) | 0.99  (0.98, 1.00) | 0.98 | 1.59 |
| ***Test Sample Type*** | **Combined Sample*** |  | 14 (34542) | 0.89  (0.75, 0.96) | 0.98  (0.94, 1.00) | 1.78 | 2.36 |
|  | **Nasal** |  | 8 (27937) | 0.82  (0.71, 0.90) | 0.99  (0.97, 1.00) | 0.81 | 1.57 |
|  | **Nasopharyngeal Sample** |  | 71 (34993) | 0.71 (0.66, 0.76) | 0.99 (0.98, 0.99) | 1.02 | 1.13 |
|  | **Oropharyngeal Sample^** |  | 3 (7384) | - | - | - | - |
|  | **Other (Breath)** |  | 1 (105) | 0.92 (0.67,0.99) | 0.99 (0.94, 0.99) | - | - |
|  |  |  |  |  |  |  |  |
| ***Symptoms*** | **Symptomatic** |  | 17 (27335) | 0.77  (0.58, 0.88) | 0.97  (0.93, 0.99) | 1.69 | 2.12 |
|  | **Asymptomatic** |  | 10 (8621) | 0.55  (0.32, 0.76) | 0.99  (0.97, 1.00) | 1.46 | 1.49 |

*** *Combined sample may include more than one of the following samples: nose, throat, mouth, saliva.*

*^ Sensitivities in the three studies: 0.59 (0.49, 0.69),* *0.64 (0.58, 0.70), 0.63 (0.54, 0.71);
 Specificities in the three studies: 0.99 [0.99, 0.99), 1.00 (0.99,1.00), 0.99 (0.99,1.00)*

# Appendix 14. Meta-regression analysis results accounting for participant age

| **Test Type** | **Covariate (interpretation)** | **# Studies  (# participants)** | **Summary Estimates** | | **Heterogeneity Standard Deviation** | |
| --- | --- | --- | --- | --- | --- | --- |
|  |  |  | **Sensitivity** | **False Positive Rate** | **Logit (Sensitivity)** | **Logit (False Positive Rate)** |
| ***Molecular*** | **Mean participant age**  **(*Relative average change in sensitivity or false positive rate with a unit increase in age)*** | 9 (1302) | - | - | - | - |
| ***Antigen*** |  | 55 (55668) | 0.50 (0.49,0.50) | 0.50  (0.50, 0.52) | 0.97 | 1.14 |

# Appendix 15. Study sensitivity and specificity per participant age category


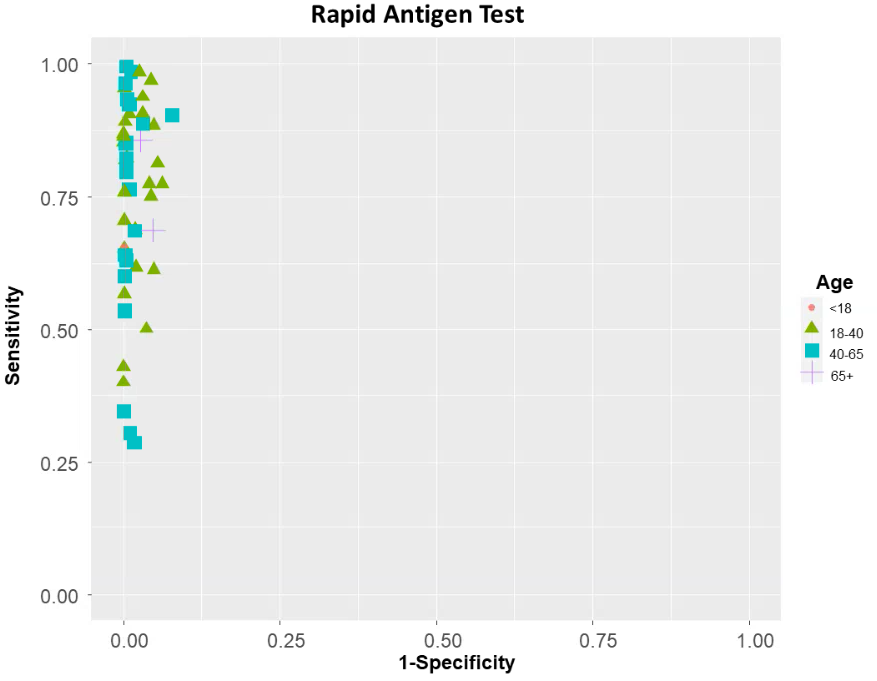


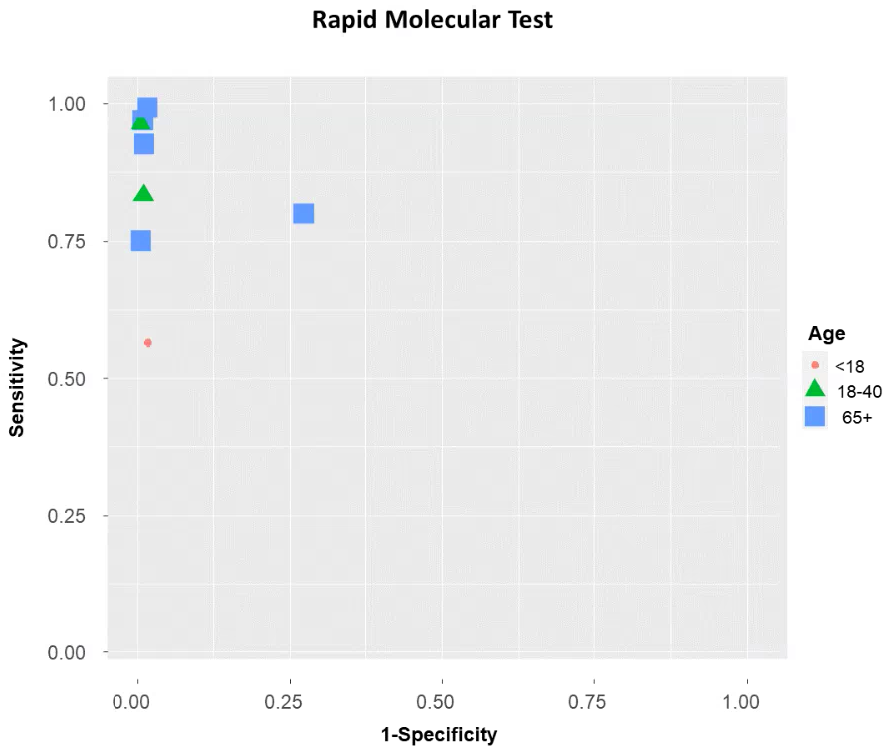


# Appendix 16. Study sensitivity and specificity per category of molecular tests


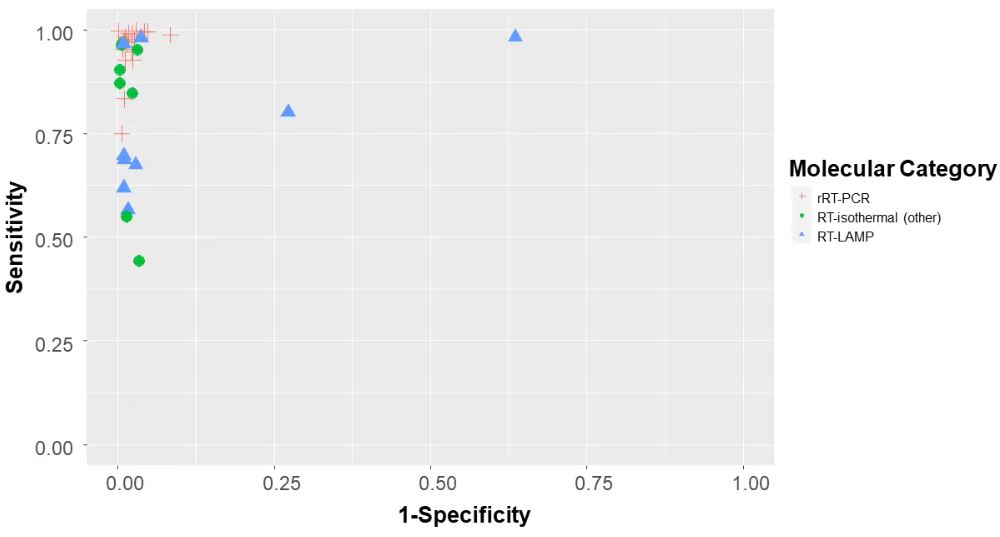

Supplement: Supplementary file 4 — Additional file 4: Appendices. This document contains supplementary information (Appendices 1–16). [file 12916_2023_2810_MOESM4_ESM.docx]
